# The cross‐sectional interplay between neurochemical profile and brain connectivity

**DOI:** 10.1002/hbm.25396

**Published:** 2021-04-09

**Authors:** George Zacharopoulos, Uzay Emir, Roi Cohen Kadosh

**Affiliations:** ^1^ Wellcome Centre for Integrative Neuroimaging, Department of Experimental Psychology University of Oxford Oxford UK; ^2^ School of Health Sciences, College of Health and Human Sciences Purdue University West Lafayette Indiana USA

**Keywords:** brain connectivity, development, glutamate, neurochemicals, parietal

## Abstract

Neurochemical profile and brain connectivity are both critical aspects of brain function. However, our knowledge of their interplay across development is currently poor. We combined single‐voxel magnetic resonance spectroscopy and resting functional magnetic resonance imaging in a cross‐sectional sample spanning from childhood to adulthood which was reassessed in ~1.5 years (*N* = 293). We revealed the developmental trajectories of 20 neurochemicals in two key developmental brain regions (the intraparietal sulcus, IPS, and the middle frontal gyrus, MFG). We found that certain neurochemicals exhibited similar developmental trajectories across the two regions, while other trajectories were region‐specific. Crucially, we mapped the connectivity of the brain regions IPS and MFG to the rest of the brain across development as a function of regional glutamate and GABA concentration. We demonstrated that glutamate concentration within the IPS is modulated by age in explaining IPS connectivity with frontal, temporal and parietal regions. In mature participants, higher glutamate within the IPS was related to more negative connectivity while the opposite pattern was found for younger participants. Our findings offer specific developmental insights on the interplay between the brain's resting activity and the glutamatergic system both of which are crucial for regulating normal functioning and are dysregulated in several clinical conditions.

## Graphical abstract

We revealed the developmental trajectories of 20 neurochemicals in two key developmental brain regions (the intraparietal sulcus, IPS, and the middle frontal gyrus, MFG). We demonstrated that glutamate concentration within the IPS is modulated by age in explaining IPS connectivity with frontal, temporal, and parietal regions. Our findings offer specific developmental insights on the interplay between the brain's resting activity and the glutamatergic system both of which are crucial for regulating normal functioning and are dysregulated in several clinical conditions.

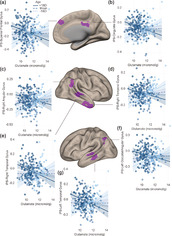

## 1 INTRODUCTION

Neurochemical profile and brain connectivity are both critical aspects of brain function. Specifically, various neurochemicals exert differential effects on brain function with excitatory (e.g., glutamate) and inhibitory (e.g., gamma‐aminobutyric acid, GABA) neurochemicals inducing neural excitation and inhibition respectively (Kandel et al., 2000). However, the impact of neurochemicals on the brain network level is constrained by brain connectivity (Dang, O'Neil, & Jagust, 2013), which is in turn influenced by regional GABA and glutamate levels (Duncan et al., 2013; Enzi et al., 2012; Kapogiannis, Reiter, Willette, & Mattson, 2013; Kwon et al., 2014; Stagg et al., 2014) suggesting a reciprocal relationship. Moreover, neurochemicals and brain connectivity are measures that span successive levels of cortical organization hierarchy, as neurochemicals affect the activity of neurons, which form micro‐scale circuits, and brain connectivity represents the state of macro‐scale networks (Kapogiannis et al., 2013). Consequently, an integrative and deeper understanding of brain function requires considering the interplay of both neurochemical concentration and brain connectivity. Moreover, alterations in neurochemicals, brain connectivity, and their relationship were documented in several clinical conditions including depression and multiple sclerosis (Gao et al., 2018; Horn et al., 2010; Kraguljac et al., 2017). Furthermore, given that both neurochemical concentration and brain connectivity change across the lifespan, it is surprising that there is a lack of understanding if and how development plays a crucial role in shaping this interplay.

Several studies examined the link between neurochemical concentration and brain connectivity to date. These studies typically assessed brain connectivity using resting functional magnetic resonance imaging (fMRI) and quantified neurochemical concentrations, such as glutamate and GABA, with single‐voxel ^1^H magnetic resonance spectroscopy (MRS; Barker, Bizzi, De Stefano, Lin, & Gullapalli, 2010). For example, GABA concentration within the primary motor cortex was associated with brain connectivity of the motor network, and regional glutamate concentration was associated with default mode network and cortical–subcortical connectivity, but most of these studies focused on adults and a single study examined infants (Duncan et al., 2013; Enzi et al., 2012; Kapogiannis et al., 2013; Kwon et al., 2014; Stagg et al., 2014). The present study, on the other hand, examines the interplay between neurochemical concentration and brain connectivity from early childhood to adulthood. The motivation for our study is rooted in the well‐established effect of development on neurochemical concentration. For example, levels of *N*‐acetylaspartate (a neuronal marker, NAA) and creatine were shown to increase while levels of glutamate were shown to decrease across development (Aoki, Inokuchi, Suwa, & Aoki, 2013; Burri et al., 1990; Gerard, Loiseau, Duchamp, & Seguin, 2002; Grachev & Apkarian, 2001; Gruber et al., 2008; Horská et al., 2002; Hüppi et al., 1991; Kreis et al., 2002; Kreis, Ernst, & Ross, 1993; Leary et al., 2000; Pfefferbaum, Adalsteinsson, Spielman, Sullivan, & Lim, 1999; Pouwels et al., 1999; Reyngoudt et al., 2012; Sailasuta, Ernst, & Chang, 2008; Saunders, Howe, van den Boogaart, Griffiths, & Brown, 1999; Yang et al., 2015). However, the evidence for these neurochemical changes in response to development is usually based on relatively modest sample sizes. Therefore, a better understanding of the changes in neurochemicals as a function of development and its interaction with brain connectivity is needed.

By combining MRS and resting fMRI technologies in a cross‐sectional design, our aim was two‐fold: to (a) delineate the developmental trajectories of a wide range of neurochemicals from early childhood to adulthood in two key developmental regions (the left intraparietal sulcus, IPS, and the left middle frontal gyrus, MFG), and to (b) investigate the interplay between neurochemicals and brain connectivity across development. We focus on the left frontal and parietal regions as their functional profile in various domains of functioning was shown to change from childhood to adulthood (Klingberg, Forssberg, & Westerberg, 2002; Rivera, Reiss, Eckert, & Menon, 2005), and thus make them suitable targets for examining the development of the neurochemical profile. Of note, the present study features secondary aims the rationale and results of which are described in Supporting Information, these include examining how the concentrations of neurochemicals are influenced by different quantification methods (Supporting Information 1, 4, and 5), by physiological parameters (Supporting Information 3), and by the biometric indices of sex, weight, and height (Supporting Information 1).

## 2 METHODS

### 2.1 Participants

We recruited 293 participants and the demographic information of our sample during the first and second assessment is depicted in Table 1. The completion of the structural, MRS, and resting fMRI session lasted (~60 min). All imaging data were acquired on a single scanning session. During the structural imaging and the MRS acquisition, participants were watching a movie (directed by Phil & Christopher, 2014), and during the resting fMRI participants fixated on a white cross on a black background. All participants self‐reported no current or past neurological, psychiatric or learning disability or any other condition that might affect brain functioning. Adult participants received £50 compensation for their time, and children participants, depending on their age, received £25 (6 years old) and £35 (10 years old, 14 years old, 16 years old) Amazon or iTunes vouchers, and additional compensation for their caregiver if below 16 years. Informed written consent was obtained from the primary caregiver and informed written assent was obtained from participants younger than 16 years according to approved institutional guidelines. The study was approved by the University of Oxford's Medical Sciences Interdivisional Research Ethics Committee (MS‐IDREC‐C2_2015_016). During the second assessment, the attrition rate was around 33% which is within the range reported previously ranging from 30 to 70% for longitudinal studies (Badawi, Eaton, Myllyluoma, Weimer, & Gallo, 1999; Bjerkeset, Nordahl, Larsson, Dahl, & Linaker, 2008; Fischer, Dornelas, & Goethe, 2001; Goodman & Blum, 1996; Miller & Wright, 1995; Tambs et al., 2009).

**TABLE 1:** Sex and mean age (standard deviation in parentheses) during the first (A1, top half) and the second (A2, bottom half) assessment

Group			
First assessment (A1)	Females	Males	Age (months)
6 years old	28	23	78.02 (3.51)
10 years old	27	24	125.35 (3.83)
14 years old	25	25	172.82 (3.96)
16 years old	56	31	202.70 (4.72)
18+ years old	21	33	226.65 (7.43)

Second assessment (A2)			
6 years old	22	22	97.66 (4.34)
10 years old	22	18	147.12 (5.74)
14 years old	16	19	193.29 (4.70)
16 years old	26	18	220.93 (6.26)
18+ years old	12	22	247.18 (7.86)

### 2.2 MRI data acquisition and preprocessing

All MRI data were acquired at the Oxford Centre for Functional MRI of the Brain (FMRIB) on a 3T Siemens MAGNETOM Prisma MRI System equipped with a 32 channel receive‐only head coil. Anatomical high‐resolution T1‐weighted scans were acquired (MPRAGE sequence: TR = 1,900 ms; TE = 3.97 ms; 192 slices; voxel size = 1 × 1 × 1 mm).

#### 2.2.1 Magnetic resonance spectroscopy

**FIGURE 1:**
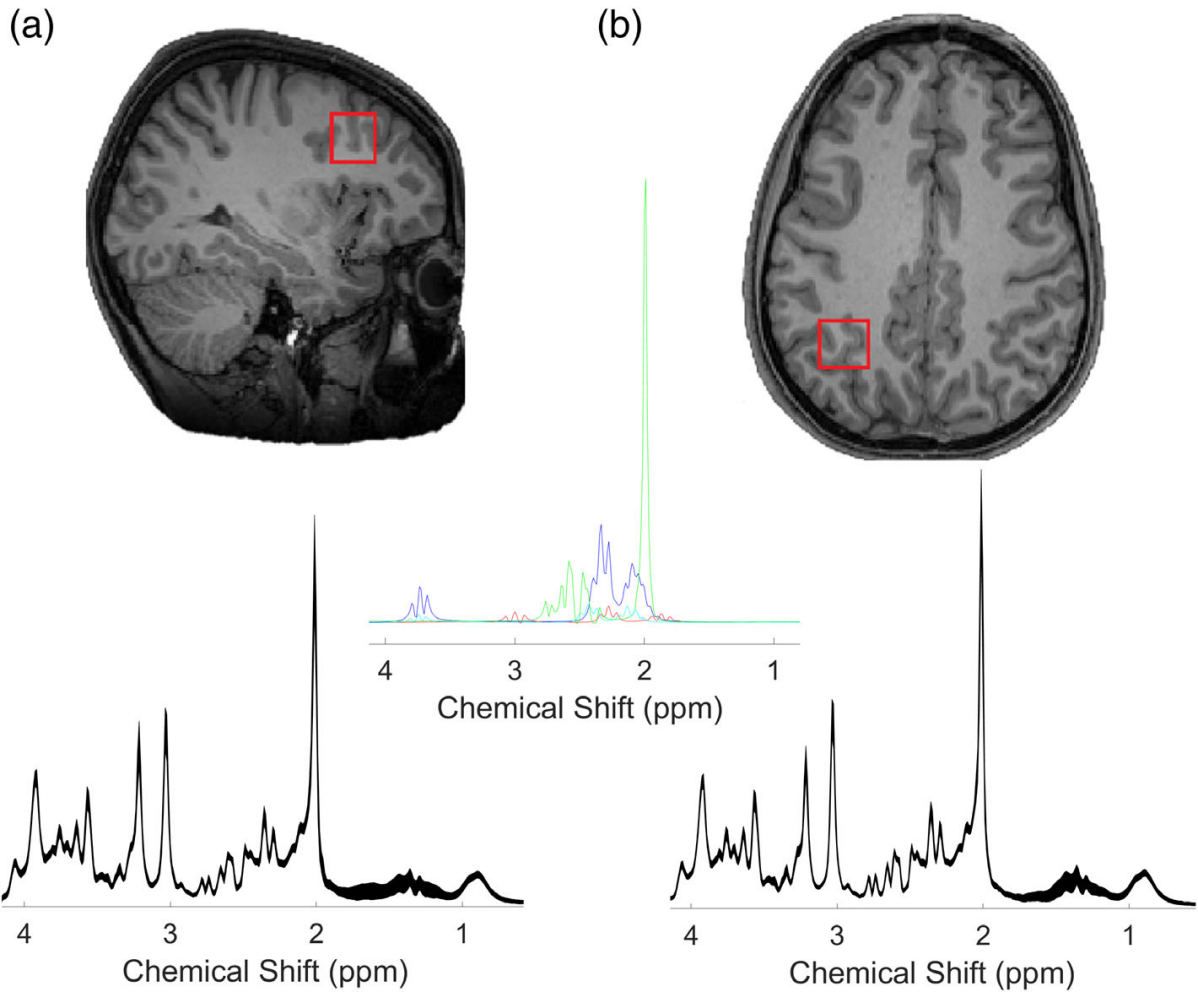
Positions of the two regions for the MRS displayed in a T1‐weighted image for (a) MFG, (b) IPS, are shown on sagittal and axial slices, respectively. Below each figure the mean spectrum form our sample at A1 of each region is shown (parts per million, ppm, in *x*‐axis), and the thickness corresponds to ±1 standard deviation from the mean. The middle panel shows the fit spectra of four neurochemicals, NAA (green), glutamate (black), GABA (red), and glutamine (cyan)

Spectra were measured by semi‐adiabatic localization using an adiabatic selective refocusing (semi‐LASER) sequence (TE = 32 ms; TR = 3.5 s; 32 averages; Deelchand et al., 2015; Öz & Tkáč, 2011) and variable power RF pulses with optimized relaxation delays (VAPOR), water suppression, and outer volume saturation. Unsuppressed water spectra acquired from the same volume of interest were used to remove residual eddy current effects and to reconstruct the phased array spectra with MRspa (https://www.cmrr.umn.edu/downloads/mrspa/ ). Two 20 × 20 × 20 mm^3^ voxels of interest were manually centered in the left intraparietal sulcus (IPS) and the left middle frontal gyrus (MFG) based on the individual's T1‐weighted image while the participants were lying down in the MR scanner. Acquisition time per voxel was 10–15 min including voxel placement and B0 shimming (Figure 1).

Neurochemicals were quantified with an LCmodel (S. W. Provencher, 2001) using a basis set of simulated spectra generated based on previously reported chemical shifts and coupling constants based on a VeSPA (versatile simulation, pulses, and analysis) simulation library (Soher, Semanchuk, Todd, Steinberg, & Young, 2011). Simulations were performed using the same RF pulses and sequence timings as in the 3T system described above. Absolute neurochemical concentrations were extracted from the spectra using a water signal as an internal reference. Apart from the detection of neurochemicals with low molecular weight, our sequence allowed the quantification of high‐molecular‐weight macromolecules. Eight LCModel‐simulated macromolecule resonances were included in the analysis at the following positions: 0.91, 1.21, 1.43, 1.67, 1.95, 2.08, 2.25, and 3 ppm (Schaller, Xin, Cudalbu, & Gruetter, 2013). Macromolecules (MM) were first characterized with ^1^H NMR in the rat (Behar & Ogino, 1993) and the human brain (Behar, Rothman, Spencer, & Petroff, 1994), and are usually denoted following their position in the spectrum in ppm: MM09 (at 0.9 ppm), MM12 (at 1.2 ppm), MM14 (at 1.4 ppm), MM17 (at 1.7 ppm), and MM20 (at 2 ppm).

The exclusion criteria for data were (a) Cramér–Rao bounds and (b) the signal‐to‐noise ratio (SNR; Emir, Tuite, & Öz, 2012). Neurochemicals quantified with Cramér–Rao lower bounds (CRLB, the estimated error of the neurochemical quantification) >50% were classified as not detected. Additionally, we excluded cases with an SNR beyond three standard deviations, and neurochemical concentration values beyond three standard deviations (per region, per neurochemical, and per age group). For the average spectrum in each of the five groups separately see Supporting Information 2.

The present study features four neurochemical concentration quantification methods termed: (a) absolute concentration referenced to water, (b) absolute concentration referenced to total creatine, (c) tissue‐corrected concentration, and (d) T2‐corrected concentration. The first neurochemical quantification method (i.e., absolute concentration referenced to water) is automatically obtained from the LCModel, and it is the water reference neurochemical concentration. The second neurochemical quantification method (i.e., absolute concentration referenced to total creatine, tCr) is also automatically obtained as part of the LCModel output and it is essentially the concentration obtained from the first quantification method but referenced to the concentration of total creatine. Please note that, no tissue and no relaxation corrections are featured in these two concentration quantification methods (i.e., absolute concentration referenced to water and absolute concentration referenced to total creatine). However, the third neurochemical quantification method (i.e., tissue‐corrected concentration), is the tissue‐corrected concentration and it is calculated based on Equation (1). Finally, apart from tissue‐correction, the forth quantification method (i.e., T2‐corrected concentration), also features T2 relaxation correction as can be seen in Equation (2).

Absolute neurochemical concentrations were then scaled based on the structural properties of the selected regions and based on the predefined values shown Equation (1) (see below; S. W. Provencher, 2001). To quantify the structural properties, we segmented the images into different tissue classes including gray matter (GM), white matter (WM), and cerebrospinal fluid (CSF) using the SPM12 segmentation. The structural templates used for the segmentation in the present study were the SPM ones and were derived from adults and thus may not be ideal in the case of segmenting pediatric brains due to differences between the adult and the children brain. Therefore our results might be affected by the adult templates but we also report the neurochemical concentration referenced by total creatine, which minimizes this bias. Next, we calculated the number of GM, WM, and CSF voxels within the two volumes of interests in native space. Subsequently, we divided these six numbers (GM, WM, and CSF for IPS and MFG, respectively) by the total number of GM, WM, and CSF voxels to obtain the corresponding GM, WM, and CSF fraction values per participant and region:

(1) $$\text{tissue}-\text{corrected concentration}=\left(\left(43300/55556*\mathrm{GM}\;\text{fraction}+35880/55556*\mathrm{WM}\;\text{fraction}+1*\mathrm{CSF}\;\text{fraction}\right)/\left(1-\mathrm{CSF}\;\text{fraction}\right)\right)*\text{absolute neurochemical concentration}$$

The values 43,300, 35,880, and 55,556 are the water concentrations in mmol/L for GM, WM, and CSF, respectively, and these are the default water concentrations values employed by LCModel (Stephen W Provencher, 2014). The numerator corrects for differing tissue water concentrations for the unsuppressed water reference, whereas the denominator corrects for the assumption that CSF is free of metabolites.

These concentration values were scaled, based on the T2 of tissue water values as can be seen in Equation (2). Fully relaxed unsuppressed water signals were acquired at TEs ranging from 32 to 4,040 ms (TR = 15 s) to water T2 values in each VOI (32, 42, 52, 85, 100, 115, 150, 250, 450, 850, 1,650, 3,250, and 4,040 ms). The transverse relaxation times (T2) of tissue water and percent CSF contribution to the VOI were obtained by fitting the integrals of the unsuppressed water spectra acquired in each VOI at different TE values with a biexponential fit (Piechnik et al., 2009), with the T2 of CSF fixed at 740 ms and three free parameters: T2 of tissue water, amplitude of tissue water, and amplitude of CSF water. The distribution of T2 values (T2 in Equation (2) below), as well as the association between T2 values and age, can be seen in Supporting Information 3.

(2) $$T2-\text{corrected concentration}=\text{tissue}-\text{corrected concentration}*\exp\left(-\mathrm{TE}/\text{T2}\right)$$

Some metabolites in our study were not detectable. We defined a neurochemical as nondetectable if it had >50% CRLB in >50% of the participants in both regions. The metabolites alanine, phosphocholine, scyllo, and *N*‐acetylaspartylglutamate (NAAG) met these criteria and any measures contained those were excluded from the analyses. Creatine and phosphocreatine had an absolute value of cross‐correlation >.5 and thus we report only their sum instead of reporting them separately (For the correlation matrix depicting the cross‐correlations see Supporting Information 7 and 8). The results reported in the main text are derived using the quantification method of Equation (1). We additionally report the neurochemical results of the other three quantification methods (T2‐corrected, absolute concentration and absolute concentrations referenced to tCr) in Supporting Information 1, 3, and 4. Images of the voxel placement during the first assessment were used during the second assessment voxel placement to ensure consistency across assessments. For additional information about conversion of units for neurochemicals, specifically from the water reference quantitation approach, see Supporting Information 10.

#### 2.2.2 Resting fMRI

Functional images were acquired with a multi‐band acquisition sequence (Multi‐band acceleration factor = 6, TR = 933 ms, TE = 33.40 ms, flip angle 64°, number of slices = 72, voxel dimension = 2 × 2 × 2, number of volumes = 380). Resting fMRI data were preprocessed and analyzed using the CONN toolbox (www.nitrc.org/projects/conn, RRID:SCR_009550) (S. Whitfield‐Gabrieli & Nieto‐Castanon, 2012) in SPM12 (Wellcome Department of Imaging Neuroscience, Institute of Neurology, London, UK) using the default preprocessing pipeline “MNI‐space direct normalization.” Functional volumes were motion‐corrected, slice‐timed corrected, segmented, normalized to a standardized (MNI) template, spatially smoothed with a Gaussian kernel (8‐mm FWHM) and bandpass filtered (0.01 Hz to Inf). Moreover, our preprocessing also involved outlier‐identification (>2 mm). We excluded cases where (a) the outlier identification step excluded more than 5% of scans, and/or (b) when the participant‐specific voxel‐to‐voxel mean correlation histogram was significantly nonzero (*r* >.15). The present study featured two seeds or regions of interest: the left MFG and the left IPS. The MFG region was defined based on the Harvard‐Oxford Atlas and the IPS which was defined based on the Dorsal Attention network atlas; both atlases are featured within the CONN toolbox. The reason we used a different atlas for the IPS is that this region is not featured in the other atlas. We run seed‐to‐voxel analyses which probe how a seed is connected to the (voxels in the) rest of the brain. We choose to focus on the seed‐to‐voxel approach than the seed‐to‐seed approaches as we aimed to examine the impact of local neurochemical concentration on the whole‐brain rather than to nodes of a particular predefined network. In other words, we choose to remain agnostic concerning the target regions and employed a more conservative seed‐to‐voxel method. The benefit of this approach is that it allowed us to empirically map a “neurochemically‐defined” network that is not based on any prior regional knowledge of the classical resting networks, as this was already been done previously (Kapogiannis et al., 2013). The connectivity between the seed and the rest of the brain was the dependent variable. The cluster‐forming *p*‐value threshold was (*p*‐FDR <.05) and the voxelwise *p*‐value threshold was (*p*‐uncorrected <.001).

### 2.3 Statistical analyses

To examine changes in neurochemical profiles across development, we employed Spearman's bivariate correlations between chronological age and neurochemical concentration and we corrected for multiple comparisons using FDR at an alpha level of .05. We employed Spearman's bivariate correlations as the variable chronological age was not normally distributed.

Our main aim was to investigate the relationship between brain connectivity and neurochemical profiles across development. In particular, we focus on glutamate and GABA levels as these neurochemicals have a known function as an excitatory and inhibitory neurotransmitter and thus are expected to affect synaptic transmission shaping brain connectivity (Kandel et al., 2000; Kapogiannis et al., 2013; Stagg et al., 2014). To examine the interaction between neurochemical concentration and age in explaining brain connectivity, we employed the following regression models. In Equation (3) below, the effect of interest was the neurochemical concentration * age interaction (highlighted in bold) after controlling for the main effects. This reveals the connections that are driven by the confluence of neurochemical concentration and age.

(3) connectivity~age+neurochemical concentration+age*neurochemical concentration

To establish the regional and neurochemical specificity of the results from Equation (3), we employed Equations (4) and (5), respectively. To establish the anatomical specificity (i.e., IPS vs. MFG), we run a variant of Equation (3) (Equation (4)) with additionally controlling the neurochemical concentration of the control region (i.e., IPS if MFG was the main region and vice versa). The effect of interest in Equation (4) is the age * neurochemical concentration of the main region interaction (highlighted in bold).

(4) connectivity~age+neurochemical concentration of the main region+age*neurochemical concentration of the main region+neurochemical concentration of the control region+neurochemical concentration of the control region*age

To establish the neurochemical specificity (i.e., glutamate vs. GABA), we run a variant of Equation (3) (Equation 5) with additionally controlling for the neurochemical concentration of a control neurochemical (i.e., GABA if glutamate was the main neurochemical and vice versa). The effect of interest in Equation (5) is the age * neurochemical concentration of the main neurochemical (highlighted in bold).

(5) connectivity~age+neurochemical concentrationof the main neurochemical+age*neurochemical concentration of the main neurochemical+neurochemical concentration of the control neurochemical+neurochemical concentration of the control neurochemical*age

Lastly, to define the cutoffs of the developmental findings we utilized the Johnson–Neyman technique (JNT; Johnson & Neyman, 1936). This technique defines the values of the moderator (age in this case) where the effect of the independent variable on the dependent variable becomes significant. In the main text, we report the upper and lower cutoff values of the moderator expressed in age in months. For the values of the moderator higher than the upper bound the relation between neurochemical concentration and brain connectivity was significantly negative, and for the values of the moderator lower than the lower bound the relation between neurochemical concentration and brain connectivity was significantly positive.

Concerning the brain connectivity regression models, the assumptions of homoscedasticity, normality of residuals and absence of multicollinearity were generally met, but we also provide *p*‐values derived from Bootstrapping using 5,000 samples at 95% confidence intervals (see Supporting Information 9).

## 3 RESULTS

### 3.1 Tracking the developmental trajectories of multiple neurochemicals

Our first aim was to delineate the developmental trajectories of neurochemical profiles in two brain regions, the left MFG and left IPS, from early childhood to adulthood. As hypothesized based on the prior work, age was a significant predictor for many neurochemical concentrations both within the MFG and within the IPS (Grachev & Apkarian, 2001). As can be seen in Table 2, in the case of MFG at the first assessment (A1), age was positively associated with the concentration of GABA, glutamine, glucose, NAA, total creatine (i.e., creatine + phosphocreatine; henceforth tCr), and the macromolecules groups MM09, MM20. Moreover, age was negatively associated with glutamate, ascorbate, glutathione, and taurine.

**TABLE 2:** Statistical results depicting the effect of age on neurochemical concentration

	A1	A2	A1	A2
	MFG	MFG	IPS	IPS
**GABA**	**0.27**	**0.35**	**0.35**	**0.37**
**Glutamate**	**−0.51**	**−0.5** **1**	**−0.5** **2**	**−0.5** **7**
**Glutamine**	**0.25**	**0.08**	**‐0.03**	**−0.18**
**Aspartate**	**−0.08**	**−0.07**	**−0.08**	**−0.20**
**Ascorbate**	**−0.45**	**−0.34**	**−0.4** **2**	**−0.4** **2**
**Glucose**	**0.19**	**0.11**	**0.08**	**0.13**
**Glycerophosphocholine**	**−0.09**	**‐0.12**	**0.26**	**0.31**
**Glutathione**	**−0.18**	**−0.20**	**−0.20**	**−0.28**
**Inositol**	**0.01**	**0.2**	**0.05**	**0.19**
**Scyllo‐Inositol**	**0.11**	**0.35**	**0.16**	**0.3** **0**
**Lactate**	**0.15**	**0.04**	**0.06**	**0.14**
**Phosphoethanolamine**	**0.02**	**0.02**	**‐0.34**	**−0.3**
***N*‐acetylaspartate**	**0.35**	**0.36**	**0.25**	**0.07**
**Taurine**	**−0.63**	**−0.43**	**−0.72**	**−0.5** **8**
**Creatine + phosphocreatine (tC r)**	**0.17**	**0.3** **5**	**0.3** **2**	**0.3** **4**
**Macromolecule 09**	**0.23**	**0.15**	**0.23**	**0.19**
**Macromolecule 20**	**0.46**	**0.36**	**0.42**	**0.36**
**Macromolecule 12**	**0.13**	**0.22**	**0.15**	**0.12**
**Macromolecule 14**	**−0.01**	**−0.15**	**−0.05**	**−0.17**
**Macromolecule 17**	**0.1**1	**0.31**	**0.09**	**−0.05**

*Note:* The Table values are the effect sizes (Spearman's correlation coefficient) between age and the neurochemical concentration (FDR‐corrected values are highlighted in bold). A1, first assessment; A2, second assessment.

In the case of IPS, age was positively associated with GABA, glycerophosphocholine, scyllo‐Inositol, NAA, tCr, MM09, MM20, and MM12. Age was negatively associated with glutamate, ascorbate, glutathione, phosphoethanolamine, and taurine (see Supporting Information 4 for the results of the other three quantification methods, see Supporting Information 5 for the scatterplots between age and the neurochemical profile with linear, quadratic, and cubic fits). The results were very similar (Table 1, and Supporting Information 4), during the reassessment even though A2 covered a less developmentally comprehensive age span.

### 3.2 Examining how development affects the interplay between neurochemical concentration and brain connectivity

**FIGURE 2:**
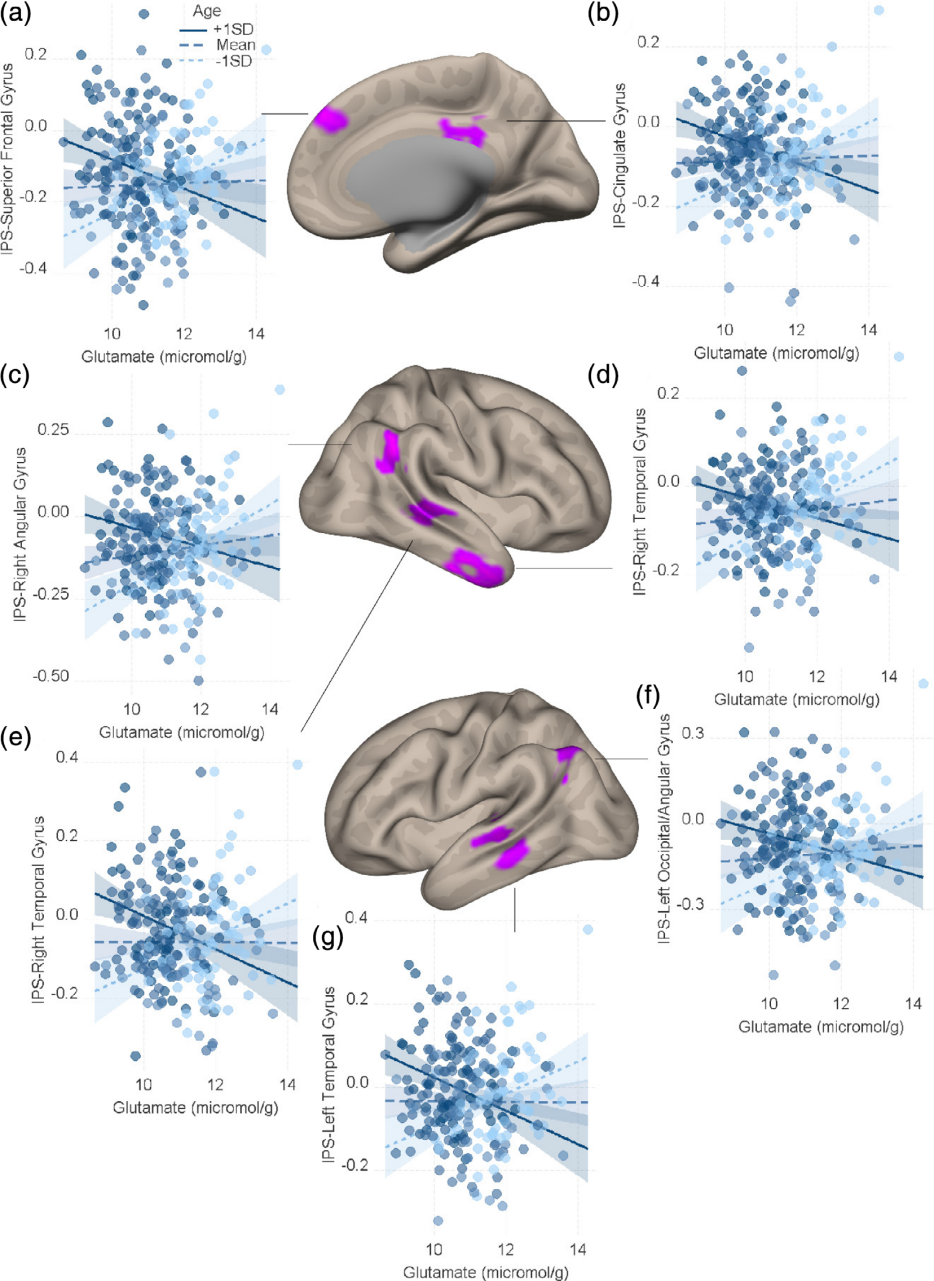
Glutamate concentration within the left intraparietal sulcus (IPS) was modulated by age in explaining the connectivity (Fisher‐*z*‐transformed correlation coefficient) between the IPS and seven regions. (a) superior frontal gyrus, (b) cingulate gyrus, (c) right angular gyrus, (d and e) right temporal gyrus, (f) left occipital/angular gyrus, and (g) left temporal gyrus. Dark blue regression lines correspond to +1 standard deviation (*SD*) above the mean age and light blue regression lines correspond to −1 *SD* from the mean age. To depict the interaction between the continuous variables (age and neurotransmitter concentration) we plotted the regression lines for ±1 *SD* from the mean age (Aiken, West, & Reno, 1991). Dark blue concerns +1 *SD* above the mean, while the light blue concerns −1 *SD* the mean and the shaded area represents 95% confidence intervals

We then examined the role of age in modulating the relationship of neurochemical concentration and brain connectivity. In particular, we focus on GABA and glutamate as these are the brain's major inhibitory and excitatory neurochemicals and thus the best neurochemical candidates for affecting brain connectivity (Kandel et al., 2000; Kapogiannis et al., 2013; Stagg et al., 2014). We mapped the connectivity between the region of interest (MFG or IPS) and the rest of the brain as a function of the interaction of neurochemical concentration and age (see Equations (3), (4), (5)) in Section 2). Glutamate in the IPS interacted with age in predicting brain connectivity between the IPS and the following regions: (1) the cingulate gyrus, (2) right inferior/middle temporal gyrus, (3) left middle temporal gyrus, (4) right angular gyrus, (5) right middle temporal gyrus, (6) superior frontal gyrus, and (7) left occipital/angular gyrus (see Table 3 and Figure 2). In all of these regions, high glutamate concentration was related to more negative connectivity in mature participants, while it was related to more positive connectivity in younger participants (see Table 3 for JNT values). We did not find any significant results for GABA in the IPS or GABA or glutamate in the case of MFG.

**TABLE 3:** The IPS‐connected target regions the connectivity of which was predicted based on the interaction between age and IPS glutamate concentrations

Region name	MNI coordinates	Number of voxels (*k*) and *p*‐FDR	JNT
Cingulate gyrus	*x* = −4, *y* = −16, *z* = 28	*k* = 922, *p*‐FDR <.0001	163, 203
Right inferior/middle temporal gyrus	*x* = 60, *y* = −16, *z* = −30	*k* = 790, *p*‐FDR = .0001	173, 215
Left middle temporal gyrus	*x* = −68, *y* = −34, *z* = −26	*k* = 774, *p*‐FDR = .0001	157, 198
Right angular gyrus	*x* = 58, *y* = −52, *z* = 38	*k* = 498, *p*‐FDR = .001	174, 220
Right middle temporal gyrus	*x* = 56, *y* = −34, *z* = −2	*k* = 489, *p*‐FDR = .001	156, 199
Superior frontal gyrus	*x* = 2, *y* = 38, *z* = 36	*k* = 485, *p*‐FDR = .001	160, 206
Left occipital/angular gyrus	*x* = −38, *y* = −56, *z* = 32	*k* = 408, *p*‐FDR = .003	165, 217

*Note:* (Left IPS in MNI coordinates = −39, −43,52) *p*‐FDR = FDR corrected value at the cluster‐level, *k* = number of voxels. JNT = Lower (left‐hand‐side) and upper (right‐hand‐side) boundaries in age in months derived from the Jonson–Neyman technique. For participants whose age was higher than the upper bound the relation between glutamate and brain connectivity was negative, for participants whose age was lower than the lower bound the relation between glutamate and brain connectivity was positive.

To assess the regional specificity of our findings we controlled for the concentration of glutamate within the MFG and its interaction with age. All the regions mentioned above were still significant, except for the right middle temporal gyrus (see Supporting Information 6). Lastly, to assess the neurochemical specificity of our findings, we controlled for the concentration of GABA within the IPS, and its interaction with age. All the regions mentioned above were still significant, except the left occipital/angular gyrus (see Supporting Information 6).

## 4 DISCUSSION

In the present study, we investigated neurochemical profile, brain connectivity, and their interplay from early childhood to adulthood by combining single‐voxel MRS and resting fMRI. Apart from replicating the results of prior work, we revealed two main findings: we (a) delineated the developmental trajectories of understudied neurochemicals in two key developmental regions, and we (b) delineated how development modulates the relationship between regional neurochemical concentration and brain connectivity.

The main aim of the present study was to investigate the neurochemical basis of brain connectivity fluctuations as a function of development. Importantly, our study was specifically designed to examine whether age modulates the impact of neurochemical concentration on brain connectivity. We found that glutamate concentration within the IPS is modulated by age in predicting connectivity between IPS and frontal, parietal, and temporal regions. What does this modulation of age on the relationship between glutamate concentration and brain connectivity tell us about how activity at a cellular level relates to long‐range network connectivity across development? The cortical organization is hierarchical in that neurons form micro‐scale circuits which in turn form nodes which then, in turn, form macro‐scale networks (Kapogiannis et al., 2013). Our study combined measures spanning these successive levels, as local glutamate concentration underlies activity of micro‐scale circuits and measures of resting fMRI assess macro‐scale networks. Glutamate elevates the Blood‐Oxygen‐Level‐Dependent (BOLD) response by increasing the metabolic rate of target neurons and surrounding astrocytes (DiNuzzo, Gili, Maraviglia, & Giove, 2011; Logothetis, 2008; Smith et al., 2002). Our study informs us about how the relationship between glutamate and BOLD‐based brain connectivity changes with development. We show that in mature participants higher glutamate within the IPS is related to more negative connectivity in temporal, frontal, and parietal target regions, and the opposite pattern was found for the younger participants. Why is high glutamate related to positive connectivity in younger participants but related to negative connectivity in the mature ones? Positive connectivity between cortical regions is thought to reflect local and distant communication (Cabral, Hugues, Sporns, & Deco, 2011; Sepulcre et al., 2010), while the interpretation of negative connectivity is more debatable. For example, it was argued that it may (a) represent a spurious effect of during data preprocessing (Murphy, Birn, Handwerker, Jones, & Bandettini, 2009), or it may (b) represent a biologically relevant signal important for understanding brain function and disease (Sporns & Betzel, 2016; Susan Whitfield‐Gabrieli & Ford, 2012), (c) or more recently that it is an epiphenomenon arising from the path‐length of positively connected regions (G. Chen, Chen, Xie, & Li, 2011; Qian et al., 2018). Therefore, we speculate that regional glutamate predicts stronger coupling of parietal‐based long‐range connections in younger participants while in the case of more complex and mature networks of the mature participants it may merely reflect the longer connectivity distances compared to the younger participants. Therefore, our findings uncover a developmental shift in the relationship between micro‐circuit‐level (glutamate measured with MRS) and a macro‐circuit level (brain connectivity measured with resting fMRI).

We additionally confirm that these effects are both regionally and neurochemically specific in that they are present even after controlling for glutamate within the MFG or GABA within the IPS. Why our finding was regionally and neurochemically specific? At least two contending theories have been proposed concerning neurochemical systems over the brain. The first proposes that neurochemical concentration in every region is shaped by genetic and environmental impacts, in this way neurochemicals in different regions may exert different influences on brain function, sensory and cognitive processing (Bachtiar, Near, Johansen‐Berg, & Stagg, 2015; Lunghi, Emir, Morrone, & Bridge, 2015; Marenco et al., 2010; Taniguchi et al., 2011). The alternative proposes that neurochemical concentration across regions might be fundamentally the same due to common embryonic origins or shared subcortical projections (Caputi, Melzer, Michael, & Monyer, 2013; J. Chen & Kriegstein, 2015; Dammerman, Flint, Noctor, & Kriegstein, 2000; Jinno et al., 2007; Picardo et al., 2011). Our results, therefore, are more in alignment with the first theory.

Another aim of the present study was to examine the developmental trajectories of multiple neurochemicals across two key developmental regions, the left MFG and IPS. Depending on the neurochemical, we observed positive, negative or no associations between concentration and age. Across both the MFG and the IPS, we showed an increase in NAA, tCr, and a decrease in glutamate, which is consistent with previous studies (Burri et al., 1990; Gerard et al., 2002; Grachev & Apkarian, 2001; Gruber et al., 2008; Horská et al., 2002; Hüppi et al., 1991; Kreis et al., 1993; Kreis et al., 2002; Leary et al., 2000; Pfefferbaum et al., 1999; Pouwels et al., 1999; Reyngoudt et al., 2012; Sailasuta et al., 2008; Saunders et al., 1999; Yang et al., 2015), although NAA and tCr relationship with age were affected by the quantification method (see Supporting Information 4). GABA, glucose and glutamine concentrations were shown to decrease with normal aging after young adulthood (Grachev & Apkarian, 2001), and the same group hypothesized an increase of these neurochemicals during normal development before young adulthood, a finding that we demonstrated here. Despite the consistency of the developmental trajectories across regions shown in the present study, previous studies stressed the importance of regional variation on neurochemical concentration a finding which we observed here for a small number of neurochemicals (Barker et al., 2010; Pouwels et al., 1999). These include the negative association between age and phosphoethanolamine concentration in IPS, and the positive associations between age and scyllo‐Inositol and glycerophosphocholine concentrations in the IPS, and between age and glucose and glutamine concentrations in the MFG. Concerning choline‐related compounds, they were previously found to increase throughout adulthood but more inconsistent findings exist during preadulthood life as some studies reported an increase, others reported a decrease and some reported no relationship (Chang, Ernst, Poland, & Jenden, 1996; Gruber et al., 2008; Kreis et al., 1993; Leary et al., 2000; Pfefferbaum et al., 1999; Pouwels et al., 1999; Raininko & Mattsson, 2010; van der Knaap et al., 1990). Indeed, these disparate findings may be accounted for differences in the regions, sample size, the voxel size, or the quantification methods (Cohen‐Gilbert, Jensen, & Silveri, 2014). In alignment with this suggestion, here we found regional differences in respect of the age to glycerophosphocholine concentration relationship. In particular, we detected an increase in the IPS but a decrease in the MFG of the choline compound glycerophosphocholine with age. Taken together, we overall replicated the associations between age and neurochemical concentration and we extend these in frontal and parietal regions and in a more comprehensive age range from early childhood to adulthood.

Apart from quantifying well‐studied neurochemicals, we revealed the developmental trajectories of under‐studied neurochemicals including antioxidants (ascorbate and glutathione), taurine and macromolecules. Indeed, our study quantified the two most abundant antioxidants in the central nervous system, glutathione, and ascorbate (vitamin C). Antioxidants counteract oxidative stress which can lead to compromised brain function in normal aging and neurodegenerative disease (Foster, 2006; Kamat et al., 2008). Glutathione helps in reducing oxidative stress and prevent potential cellular damage (Berk, Ng, Dean, Dodd, & Bush, 2008). Therefore, it was suggested that tracking the concentrations of antioxidants can allow the investigation of the mechanisms involved in normal development, aging, neurodegenerative disease, and therapy (Terpstra, Ugurbil, & Tkac, 2010). In particular, evidence on the glutathione‐induced actions came from animal work where glutathione pharmacological experiments demonstrated its potential benefit on neuronal hyperexcitability. For example, administration of glutathione inhibited pentylenetetrazol‐induced seizures in mice, and a specific inhibitor of glutathione biosynthesis had the opposite effect (Abe, Nakanishi, & Saito, 2000). One proposed mechanism involved glutathione's antagonistic action on *N*‐methyl‐d‐aspartate (NMDA) receptors (Levy, Sucher, & Lipton, 1991). Our evidence showed antioxidant concentration decrease across development perhaps reflecting reduced demands for controlling hyperexcitability within the central nervous system later in life, once the major synaptic pathways have been formed. In alignment with this, previous work from the authors showed that glutathione levels decrease over development when comparing young versus old adults (Emir et al., 2011).

Taurine is the second most abundant amino‐acid in the brain, and our study revealed a reduction in taurine levels over human development (Griffin & Bradshaw, 2017; Kim et al., 2014). Taurine was linked to plasticity as it was shown to increase hippocampal neurogenesis in aging mice (Gebara, Udry, Sultan, & Toni, 2015). Moreover, reduction in brain taurine concentrations was related to cognitive deficits, and taurine administration was shown to ameliorate these deficits and improve synaptic plasticity (Suárez, Muñoz, Del Río, & Solís, 2016) (Yu et al., 2007). Therefore, the documented decrease in taurine over development may reflect the progressively reduced need for plasticity as the organization of the nervous system is being matured and stabilized. Indeed taurine was shown to decrease in animals across development (Dawson, Eppler, Patterson, Shih, & Liu, 1996).

Apart from the detection of neurochemicals with low molecular weight, our sequence allowed the quantification of high‐molecular‐weight macromolecules (MM) their signal of which originate from cytosolic proteins and includes numerous resonances with a complex pattern (Koob et al., 2016). Specifically, MM09 and MM12 correspond both to the macromolecule resonances of methylene and methyl groups of mobile lipids, and the resonances of amino acids of mobile polypeptide chains (Koob et al., 2016). The resonances of MM20 are believed to be attributed to proteins and macromolecules present in brain tissue were primarily assigned to methyl and methylene resonances of protein amino acids such as leucine (Behar et al., 1994; Hofmann, Slotboom, Boesch, & Kreis, 2001). Very few studies investigated the effect of development on brain macromolecule levels with mixed results and prior work emphasized the need for accurately assessing macromolecules in normal development (Hofmann et al., 2001; Mader et al., 2002; Považan et al., 2015). The present study meets this objective by mapping the developmental trajectories of several macromolecules. We reveal an increase of parietal and frontal concentration of MM09 and MM20 throughout development suggesting reduced protein and mobile lipid synthesis early in development (Koob et al., 2016).

In sum, by combining MRS with resting fMRI we examined the interplay between neurochemicals and brain connectivity from early childhood to adulthood. First, we tracked the developmental trajectory of multiple neurochemicals in key developmental regions, revealing novel associations, and highlighted their developmental significance. Importantly, we delineated the role of age in shaping the relationship between parietal glutamate and parietal‐based brain connectivity. Our findings offer specific developmental insights on the interplay between the brain's resting activity and the glutamatergic system both of which are crucial for regulating normal functioning and are dysregulated in several clinical conditions.

## CONFLICT OF INTERESTS

The authors declare no potential conflict of interest.

## AUTHOR CONTRIBUTIONS

Roi Cohen Kadosh conceived the idea; Roi Cohen Kadosh, Uzay Emir designed the experiments; George Zacharopoulos performed the experiments; George Zacharopoulos, Uzay Emir analyzed the data, George Zacharopoulos, Uzay Emir, and Roi Cohen Kadosh wrote the article.

## Supporting information


**Appendix** S1: Supporting Information.

## Data Availability

Raw image files used in the figures and other measures that support the findings of this study are available from the corresponding authors upon reasonable request.
