# Supplementary material for: The cross‐sectional interplay between neurochemical profile and brain connectivity
Source: Hum Brain Mapp. 2021 Apr 9;42(9):2722–33. doi: 10.1002/hbm.25396 (PMC8127145; doi:10.1002/hbm.25396)
Supplement: Supplementary file 1 — Appendix S1: Supporting Information. [file HBM-42-2722-s001.docx]

**Supporting Information for**

**The cross-sectional interplay between neurochemical profile and brain connectivity**

George Zacharopoulos^1*^, Uzay Emir^1,2^, Roi Cohen Kadosh^1^

**Affiliations:**

^1^Wellcome Centre for Integrative Neuroimaging, Department of Experimental Psychology, University of Oxford, UK

^2^School of Health Sciences, College of Health and Human Sciences, Purdue University, USA, IN 47907-2051

*Correspondence:

George Zacharopoulos

Wellcome Centre for Integrative Neuroimaging, Department of Experimental Psychology, University of Oxford, UK

Psychology, University of Oxford, Oxford, OX2 6GG

E-mail: [george.zacharopoulos@psy.ox.ac.uk](mailto:george.zacharopoulos@psy.ox.ac.uk)

**Supporting Information**

**Supporting Information 1. Biometric predictors and neurochemical concentration.**

Apart from examining the influence of age in the main text, another aim of this study was to characterize the importance of the biometric indices of sex, height, and weight in determining neurochemical concentration. Biometric or physiological parameters are known to differ between men and women and to change with age and were shown to be related to plasma and urine metabolite patterns (Rist et al., 2017). In particular, prior work which studied the effect of sex yielded some conflicting results. Several studies reported no sex effects (Charles et al., 1994; Nagae‐Poetscher et al., 2004), while others reported higher GABA and Glx (glutamate+glutamine) and glutamate concentrations within the dorsolateral prefrontal cortex in males (O'Gorman, Michels, Edden, Murdoch, & Martin, 2011). Moreover, sex differences in neurochemicals were observed in schizophrenic patients, in that males (vs. females) exhibited a higher concentration of glutamine in the anterior cingulate cortex, and lower creatine and N-acetylaspartate in the left basal ganglia (Tayoshi et al., 2009). However, the above studies had a relatively small sample, making them more prone to type I error (Button et al., 2013). Therefore, the present study also aims at discerning the combination of biometric indices that best tracks the concentration of multiple neurochemicals in the MFG and IPS. As shown below, across both the MFG and the IPS, weight was positively related to glucose concertation. Intracerebral glucose concentration is transported over the blood-brain barrier by the glucose transporter GLUT1 via diffusion (Pardridge, Boado, & Farrell, 1990), and brain hypometabolism of glucose was observed in anorexia nervosa which normalized with weight gain (Delvenne et al., 1996). Weight was additionally uniquely positively contributed to most macromolecule concentrations (MM09, MM20, MM12 in the case of IPS). Apart from weight, macromolecular concentration was also predicted by sex, where male participants exhibited lower concentration than females (MM17 in MFG, MM09, MM20 MM14 in IPS). As discussed in the main text, glycerophosphocholine concentration increased over development in the IPS, agreeing with and extending previous studies. However, we additionally found that the unique contribution of sex in explaining glycerophosphocholine levels. In particular, male individuals have more concentration of glycerophosphocholine in the IPS than females. Apart from advancing our understating on the determinants of glycerophosphocholine, our finding may additionally account for the previous inconsistent results (Cohen-Gilbert, Jensen, & Silveri, 2014), emphasizing the role of equating people’s sex when studying the effects of age on choline-based compounds.

**The effect of sex on neurochemical concentration using four distinct neurochemical quantification methods 1.1.** (T=T-statistic, positive values suggest males have a higher concentration than females). MFG=left middle frontal gyrus, IPS=left intraparietal sulcus, DF=degrees of freedom, BF=Bayes Factor ratio of the model that contains sex as the predictor vs. the model merely containing the intercept). First assessment=A1, Second assessment=A2.

**Supporting Information 1.1.1. The effect of sex on neurochemical concentration using the quantification method of equation 1.**

|  | **MFG**  **MFG**  **MFG**  **MFG**  **MFG**  **MFG**  **MFG**  **MFG** | | | | | | | | **IPS**  **IPS**  **IPS**  **IPS**  **IPS**  **IPS**  **IPS**  **IPS** | | | | | | | | |
| --- | --- | --- | --- | --- | --- | --- | --- | --- | --- | --- | --- | --- | --- | --- | --- | --- | --- |
|  | **A1** | **A1** | **A1** | **A1** | **A2** | **A2** | **A2** | **A2** | **A1** | **A1** | **A1** | **A1** | **A2** | **A2** | **A2** | **A2** |  |
|  | **T** | **P** | **DF** | **BF** | **T** | **P** | **DF** | **BF** | **T** | **P** | **DF** | **BF** | **T** | **P** | **DF** | **BF** |  |
| GABA | 0.28 | 0.782 | 255 | 0.14 | -0.03 | 0.974 | 175 | 0.16 | -1.41 | 0.160 | 264 | 0.35 | -1.03 | 0.302 | 183 | 0.26 |  |
| Glutamate | -0.34 | 0.736 | 261 | 0.14 | -0.55 | 0.581 | 176 | 0.19 | 0.19 | 0.848 | 264 | 0.14 | -0.15 | 0.883 | 183 | 0.16 |  |
| Glutamine | 1.80 | 0.074 | 260 | 0.62 | 2.08 | 0.039 | 177 | 1.20 | 1.40 | 0.162 | 263 | 0.34 | 0.53 | 0.596 | 182 | 0.18 |  |
| Aspartate | 2.12 | 0.035 | 260 | 1.13 | 1.70 | 0.091 | 176 | 0.62 | 0.48 | 0.635 | 265 | 0.15 | 0.33 | 0.743 | 183 | 0.17 |  |
| Ascorbate | -0.49 | 0.623 | 251 | 0.15 | 0.38 | 0.706 | 172 | 0.18 | 0.85 | 0.394 | 263 | 0.19 | -0.93 | 0.353 | 179 | 0.24 |  |
| Glucose | -0.62 | 0.538 | 261 | 0.16 | -0.84 | 0.400 | 177 | 0.23 | 0.50 | 0.619 | 261 | 0.15 | -1.02 | 0.307 | 181 | 0.26 |  |
| Glycerophosphocholine | 0.14 | 0.889 | 261 | 0.14 | 1.19 | 0.234 | 177 | 0.31 | 3.37 | 0.001 | 263 | 27.32 | 2.27 | 0.024 | 183 | 1.72 |  |
| Glutathione | 2.36 | 0.019 | 260 | 1.87 | 1.80 | 0.073 | 177 | 0.73 | 1.37 | 0.171 | 264 | 0.33 | 1.77 | 0.078 | 183 | 0.68 |  |
| Inositol | 1.48 | 0.141 | 262 | 0.38 | 0.79 | 0.429 | 177 | 0.22 | 0.79 | 0.429 | 264 | 0.18 | 0.48 | 0.631 | 183 | 0.18 |  |
| Scyllo-Inositol | -0.24 | 0.814 | 254 | 0.14 | 1.03 | 0.302 | 171 | 0.27 | -0.32 | 0.753 | 255 | 0.14 | -0.34 | 0.736 | 180 | 0.17 |  |
| Lactate | -0.01 | 0.991 | 196 | 0.15 | 0.39 | 0.696 | 129 | 0.20 | -0.30 | 0.765 | 197 | 0.16 | 0.82 | 0.414 | 134 | 0.25 |  |
| Phosphoethanolamine | -1.72 | 0.086 | 260 | 0.55 | 0.04 | 0.969 | 175 | 0.16 | 0.51 | 0.610 | 265 | 0.15 | -0.54 | 0.591 | 181 | 0.18 |  |
| N-acetylaspartate | -0.70 | 0.483 | 260 | 0.17 | 0.05 | 0.961 | 176 | 0.16 | -0.26 | 0.793 | 265 | 0.14 | -1.26 | 0.209 | 183 | 0.33 |  |
| Taurine | -1.30 | 0.195 | 114 | 0.43 | -0.20 | 0.844 | 76 | 0.24 | -0.82 | 0.415 | 153 | 0.24 | -0.69 | 0.493 | 101 | 0.26 |  |
| Creatine+Phosphocreatine (tCr) | -0.56 | 0.579 | 260 | 0.16 | -0.10 | 0.917 | 176 | 0.16 | 0.27 | 0.785 | 264 | 0.14 | 0.15 | 0.884 | 182 | 0.16 |  |
| Macromolecule 09 | -0.86 | 0.393 | 259 | 0.19 | -1.91 | 0.057 | 178 | 0.88 | -2.98 | 0.003 | 262 | 8.61 | -2.66 | 0.008 | 180 | 4.20 |  |
| Macromolecule 20 | -0.22 | 0.825 | 259 | 0.14 | -1.20 | 0.233 | 177 | 0.31 | -2.13 | 0.034 | 263 | 1.15 | -1.27 | 0.205 | 183 | 0.34 |  |
| Macromolecule 12 | 0.20 | 0.841 | 252 | 0.14 | -1.15 | 0.252 | 168 | 0.31 | -2.03 | 0.043 | 260 | 0.95 | -0.11 | 0.914 | 176 | 0.16 |  |
| Macromolecule 14 | -0.75 | 0.456 | 237 | 0.18 | -1.99 | 0.049 | 170 | 1.02 | -4.93 | 0.000 | 249 | 9076.50 | -2.42 | 0.016 | 173 | 2.43 |  |
| Macromolecule 17 | -2.67 | 0.008 | 204 | 4.17 | -2.08 | 0.040 | 143 | 1.27 | -1.69 | 0.092 | 155 | 0.64 | -2.20 | 0.030 | 115 | 1.70 |  |

**Supporting Information 1.1.2. The effect of sex on neurochemical concentration using the absolute concentration.**

|  | **MFG**  **MFG**  **MFG**  **MFG**  **MFG**  **MFG**  **MFG**  **MFG** | | | | | | | | **IPS**  **IPS**  **IPS**  **IPS**  **IPS**  **IPS**  **IPS**  **IPS** | | | | | | | | |
| --- | --- | --- | --- | --- | --- | --- | --- | --- | --- | --- | --- | --- | --- | --- | --- | --- | --- |
|  | **A1** | **A1** | **A1** | **A1** | **A2** | **A2** | **A2** | **A2** | **A1** | **A1** | **A1** | **A1** | **A2** | **A2** | **A2** | **A2** |  |
|  | **T** | **P** | **DF** | **BF** | **T** | **P** | **DF** | **BF** | **T** | **P** | **DF** | **BF** | **T** | **P** | **DF** | **BF** |  |
| GABA | 0.36 | 0.719 | 255 | 0.15 | 0.10 | 0.923 | 175 | 0.16 | -1.80 | 0.073 | 264 | 0.63 | -1.12 | 0.264 | 183 | 0.29 |  |
| Glutamate | 0.25 | 0.802 | 261 | 0.14 | -0.27 | 0.788 | 176 | 0.17 | 0.19 | 0.848 | 264 | 0.14 | -0.19 | 0.850 | 183 | 0.16 |  |
| Glutamine | 1.91 | 0.058 | 260 | 0.76 | 2.18 | 0.030 | 177 | 1.45 | 1.43 | 0.155 | 262 | 0.35 | 0.71 | 0.481 | 181 | 0.20 |  |
| Aspartate | 2.37 | 0.019 | 260 | 1.91 | 1.92 | 0.056 | 176 | 0.90 | 0.06 | 0.953 | 264 | 0.14 | 0.35 | 0.723 | 183 | 0.17 |  |
| Ascorbate | -0.41 | 0.684 | 251 | 0.15 | 0.50 | 0.616 | 172 | 0.18 | 0.44 | 0.657 | 262 | 0.15 | -0.96 | 0.341 | 179 | 0.25 |  |
| Glucose | -0.53 | 0.596 | 261 | 0.16 | -0.71 | 0.480 | 177 | 0.20 | 0.84 | 0.402 | 263 | 0.19 | -0.98 | 0.328 | 181 | 0.25 |  |
| Glycerophosphocholine | 0.10 | 0.922 | 260 | 0.14 | 1.32 | 0.188 | 177 | 0.36 | 2.79 | 0.006 | 263 | 5.25 | 1.93 | 0.055 | 182 | 0.90 |  |
| Glutathione | 2.65 | 0.009 | 260 | 3.71 | 1.96 | 0.052 | 177 | 0.95 | 1.14 | 0.257 | 264 | 0.25 | 1.72 | 0.087 | 182 | 0.63 |  |
| Inositol | 1.76 | 0.079 | 262 | 0.59 | 1.52 | 0.130 | 177 | 0.47 | 0.67 | 0.502 | 263 | 0.17 | 0.49 | 0.624 | 183 | 0.18 |  |
| Scyllo-Inositol | 0.03 | 0.975 | 255 | 0.14 | 1.10 | 0.272 | 171 | 0.29 | -0.40 | 0.690 | 255 | 0.15 | -0.31 | 0.756 | 180 | 0.17 |  |
| Lactate | -0.30 | 0.761 | 196 | 0.16 | 0.45 | 0.656 | 129 | 0.20 | -0.15 | 0.883 | 196 | 0.16 | 0.86 | 0.392 | 134 | 0.26 |  |
| Phosphoethanolamine | -2.15 | 0.033 | 258 | 1.21 | 0.23 | 0.821 | 175 | 0.17 | 0.23 | 0.816 | 265 | 0.14 | -0.37 | 0.712 | 182 | 0.17 |  |
| N-acetylaspartate | -0.15 | 0.878 | 259 | 0.14 | 0.33 | 0.744 | 175 | 0.17 | -1.04 | 0.297 | 263 | 0.23 | -1.27 | 0.205 | 183 | 0.34 |  |
| Taurine | -1.32 | 0.191 | 114 | 0.43 | -0.24 | 0.815 | 76 | 0.24 | -0.91 | 0.365 | 153 | 0.25 | -0.75 | 0.457 | 101 | 0.27 |  |
| Creatine+Phosphocreatine (tCr) | -0.32 | 0.749 | 260 | 0.14 | 0.44 | 0.659 | 176 | 0.18 | -0.20 | 0.840 | 264 | 0.14 | -0.10 | 0.923 | 183 | 0.16 |  |
| Macromolecule 09 | -0.72 | 0.472 | 259 | 0.17 | -1.77 | 0.079 | 178 | 0.69 | -3.39 | 0.001 | 262 | 29.41 | -2.75 | 0.007 | 180 | 5.16 |  |
| Macromolecule 20 | -0.06 | 0.949 | 257 | 0.14 | -1.07 | 0.288 | 177 | 0.27 | -2.44 | 0.015 | 263 | 2.26 | -1.22 | 0.225 | 182 | 0.32 |  |
| Macromolecule 12 | 0.24 | 0.811 | 252 | 0.14 | -1.10 | 0.274 | 168 | 0.29 | -1.99 | 0.047 | 259 | 0.89 | -0.09 | 0.927 | 176 | 0.16 |  |
| Macromolecule 14 | -0.68 | 0.495 | 237 | 0.18 | -1.91 | 0.058 | 170 | 0.88 | -5.04 | 0.000 | 248 | 14798.97 | -2.52 | 0.013 | 173 | 3.01 |  |
| Macromolecule 17 | -2.63 | 0.009 | 204 | 3.76 | -1.95 | 0.053 | 143 | 1.01 | -1.92 | 0.057 | 155 | 0.93 | -2.42 | 0.017 | 115 | 2.68 |  |

**Supporting Information 1.1.3. The effect of sex on neurochemical concentration using the relative quantification method which divides the absolute quantification method to the concentration of creatine+phosphocreatine.** This explains why there are NA values in the creatine+phoshocreatine column below.

|  | **MFG**  **MFG**  **MFG**  **MFG**  **MFG**  **MFG**  **MFG**  **MFG** | | | | | | | | **IPS**  **IPS**  **IPS**  **IPS**  **IPS**  **IPS**  **IPS**  **IPS** | | | | | | | | |
| --- | --- | --- | --- | --- | --- | --- | --- | --- | --- | --- | --- | --- | --- | --- | --- | --- | --- |
|  | **A1** | **A1** | **A1** | **A1** | **A2** | **A2** | **A2** | **A2** | **A1** | **A1** | **A1** | **A1** | **A2** | **A2** | **A2** | **A2** |  |
|  | **T** | **P** | **DF** | **BF** | **T** | **P** | **DF** | **BF** | **T** | **P** | **DF** | **BF** | **T** | **P** | **DF** | **BF** |  |
| GABA | 0.06 | 0.956 | 253 | 0.14 | -0.04 | 0.972 | 175 | 0.16 | -1.74 | 0.083 | 263 | 0.57 | -1.13 | 0.261 | 183 | 0.29 |  |
| Glutamate | 0.04 | 0.970 | 261 | 0.14 | -0.62 | 0.538 | 177 | 0.19 | -0.02 | 0.981 | 265 | 0.13 | 0.05 | 0.960 | 182 | 0.16 |  |
| Glutamine | 1.81 | 0.072 | 258 | 0.64 | 1.64 | 0.102 | 176 | 0.57 | 1.07 | 0.287 | 263 | 0.23 | 0.37 | 0.715 | 183 | 0.17 |  |
| Aspartate | 2.21 | 0.028 | 260 | 1.35 | 1.51 | 0.132 | 175 | 0.47 | 0.17 | 0.867 | 265 | 0.14 | 0.38 | 0.702 | 183 | 0.17 |  |
| Ascorbate | -0.24 | 0.810 | 251 | 0.14 | 0.49 | 0.621 | 172 | 0.18 | 0.33 | 0.740 | 263 | 0.14 | -1.05 | 0.295 | 180 | 0.27 |  |
| Glucose | -0.72 | 0.472 | 260 | 0.17 | -0.70 | 0.487 | 177 | 0.20 | 0.92 | 0.360 | 263 | 0.20 | -0.92 | 0.359 | 181 | 0.24 |  |
| Glycerophosphocholine | -0.18 | 0.857 | 260 | 0.14 | 1.38 | 0.171 | 178 | 0.39 | 2.74 | 0.007 | 264 | 4.60 | 1.91 | 0.058 | 182 | 0.86 |  |
| Glutathione | 2.28 | 0.023 | 260 | 1.59 | 1.40 | 0.165 | 176 | 0.40 | 1.11 | 0.266 | 265 | 0.24 | 1.89 | 0.061 | 183 | 0.83 |  |
| Inositol | 2.10 | 0.037 | 262 | 1.08 | 0.77 | 0.444 | 177 | 0.21 | 0.88 | 0.379 | 264 | 0.19 | 0.60 | 0.547 | 183 | 0.19 |  |
| Scyllo-Inositol | 0.08 | 0.938 | 255 | 0.14 | 1.05 | 0.296 | 171 | 0.27 | -0.43 | 0.670 | 255 | 0.15 | -0.64 | 0.525 | 179 | 0.19 |  |
| Lactate | -0.41 | 0.679 | 195 | 0.17 | 0.37 | 0.709 | 129 | 0.20 | -0.20 | 0.842 | 196 | 0.16 | 0.86 | 0.394 | 134 | 0.26 |  |
| Phosphoethanolamine | -2.45 | 0.015 | 256 | 2.33 | -0.12 | 0.908 | 176 | 0.16 | 0.12 | 0.907 | 264 | 0.14 | -0.24 | 0.808 | 182 | 0.16 |  |
| N-acetylaspartate | -0.10 | 0.919 | 262 | 0.14 | -0.39 | 0.701 | 176 | 0.17 | -0.93 | 0.354 | 264 | 0.20 | -1.02 | 0.307 | 183 | 0.26 |  |
| Taurine | -1.10 | 0.274 | 114 | 0.34 | -0.24 | 0.810 | 76 | 0.24 | -0.93 | 0.353 | 153 | 0.26 | -0.55 | 0.582 | 101 | 0.24 |  |
| Creatine+Phosphocreatine (tCr) | ΝΑ | ΝΑ | ΝΑ | ΝΑ | ΝΑ | ΝΑ | ΝΑ | ΝΑ | ΝΑ | ΝΑ | ΝΑ | ΝΑ | ΝΑ | ΝΑ | ΝΑ | ΝΑ |  |
| Macromolecule 09 | -0.70 | 0.486 | 258 | 0.17 | -1.81 | 0.072 | 178 | 0.73 | -3.18 | 0.002 | 262 | 15.34 | -2.14 | 0.034 | 181 | 1.32 |  |
| Macromolecule 20 | 0.02 | 0.985 | 257 | 0.14 | -1.15 | 0.250 | 177 | 0.30 | -2.45 | 0.015 | 263 | 2.30 | -1.17 | 0.243 | 182 | 0.30 |  |
| Macromolecule 12 | 0.27 | 0.786 | 252 | 0.14 | -1.51 | 0.132 | 167 | 0.48 | -2.01 | 0.046 | 259 | 0.91 | -0.06 | 0.952 | 176 | 0.16 |  |
| Macromolecule 14 | -0.67 | 0.501 | 237 | 0.18 | -2.00 | 0.047 | 170 | 1.05 | -4.94 | 0.000 | 248 | 9785.24 | -2.41 | 0.017 | 173 | 2.36 |  |
| Macromolecule 17 | -2.55 | 0.012 | 204 | 3.10 | -2.50 | 0.014 | 142 | 3.03 | -1.91 | 0.058 | 155 | 0.92 | -2.23 | 0.028 | 115 | 1.81 |  |

**Supporting Information 1.1.4. The effect of sex on neurochemical concentration using the quantification method of equation 2.**

|  | **MFG**  **MFG**  **MFG**  **MFG**  **MFG**  **MFG**  **MFG**  **MFG** | | | | | | | | | **IPS**  **IPS**  **IPS**  **IPS**  **IPS**  **IPS**  **IPS**  **IPS** | | | | | | | | |
| --- | --- | --- | --- | --- | --- | --- | --- | --- | --- | --- | --- | --- | --- | --- | --- | --- | --- | --- |
|  | **A1** | **A1** | **A1** | **A1** | **A2** | **A2** | **A2** | **A2** | **A1** | | **A1** | **A1** | **A1** | **A2** | **A2** | **A2** | **A2** |  |
|  | **T** | **P** | **DF** | **BF** | **T** | **P** | **DF** | **BF** | **T** | | **P** | **DF** | **BF** | **T** | **P** | **DF** | **BF** |  |
| GABA | 0.18 | 0.859 | 253 | 0.14 | 0.12 | 0.905 | 174 | 0.16 | -1.39 | | 0.166 | 264 | 0.34 | -0.95 | 0.343 | 183 | 0.24 |  |
| Glutamate | -0.26 | 0.798 | 258 | 0.14 | -0.57 | 0.567 | 175 | 0.19 | 0.28 | | 0.780 | 264 | 0.14 | 0.00 | 0.999 | 183 | 0.16 |  |
| Glutamine | 1.58 | 0.115 | 260 | 0.44 | 2.17 | 0.032 | 176 | 1.42 | 1.43 | | 0.155 | 263 | 0.35 | 0.54 | 0.592 | 182 | 0.18 |  |
| Aspartate | 2.05 | 0.041 | 259 | 1.00 | 1.64 | 0.102 | 175 | 0.57 | 0.57 | | 0.570 | 265 | 0.16 | 0.41 | 0.683 | 183 | 0.17 |  |
| Ascorbate | -0.67 | 0.500 | 251 | 0.17 | -0.02 | 0.983 | 170 | 0.17 | 0.87 | | 0.383 | 263 | 0.19 | -0.80 | 0.426 | 179 | 0.22 |  |
| Glucose | -0.67 | 0.504 | 260 | 0.17 | -0.68 | 0.500 | 175 | 0.20 | 0.56 | | 0.577 | 261 | 0.16 | -0.93 | 0.352 | 181 | 0.24 |  |
| Glycerophosphocholine | 0.13 | 0.894 | 260 | 0.14 | 1.00 | 0.318 | 176 | 0.26 | 3.47 | | 0.001 | 263 | 37.90 | 2.49 | 0.014 | 183 | 2.79 |  |
| Glutathione | 2.47 | 0.014 | 260 | 2.41 | 1.65 | 0.101 | 176 | 0.57 | 1.21 | | 0.226 | 263 | 0.27 | 1.74 | 0.083 | 183 | 0.65 |  |
| Inositol | 1.42 | 0.156 | 261 | 0.35 | 0.61 | 0.542 | 176 | 0.19 | 0.88 | | 0.381 | 264 | 0.19 | 0.62 | 0.537 | 183 | 0.19 |  |
| Scyllo-Inositol | -0.30 | 0.765 | 255 | 0.14 | 1.01 | 0.314 | 170 | 0.26 | -0.25 | | 0.800 | 255 | 0.14 | -0.27 | 0.790 | 180 | 0.17 |  |
| Lactate | -0.23 | 0.821 | 194 | 0.16 | 0.45 | 0.651 | 128 | 0.21 | -0.18 | | 0.855 | 197 | 0.16 | 0.88 | 0.381 | 134 | 0.26 |  |
| Phosphoethanolamine | -1.68 | 0.094 | 259 | 0.52 | -0.06 | 0.951 | 175 | 0.16 | 0.44 | | 0.664 | 264 | 0.15 | -0.37 | 0.710 | 181 | 0.17 |  |
| N-acetylaspartate | -0.48 | 0.630 | 258 | 0.15 | 0.10 | 0.917 | 175 | 0.16 | -0.26 | | 0.794 | 264 | 0.14 | -0.93 | 0.353 | 183 | 0.24 |  |
| Taurine | -0.91 | 0.364 | 114 | 0.29 | -0.26 | 0.797 | 75 | 0.24 | -0.66 | | 0.511 | 153 | 0.21 | -0.57 | 0.568 | 101 | 0.24 |  |
| Creatine+Phosphocreatine (tCr) | -0.51 | 0.610 | 259 | 0.15 | -0.06 | 0.950 | 174 | 0.16 | 0.41 | | 0.685 | 264 | 0.15 | 0.37 | 0.709 | 182 | 0.17 |  |
| Macromolecule 09 | -0.72 | 0.470 | 259 | 0.17 | -1.87 | 0.063 | 177 | 0.82 | -2.89 | | 0.004 | 262 | 6.82 | -2.54 | 0.012 | 180 | 3.14 |  |
| Macromolecule 20 | -0.23 | 0.820 | 257 | 0.14 | -1.30 | 0.195 | 177 | 0.36 | -2.09 | | 0.037 | 263 | 1.07 | -1.05 | 0.294 | 182 | 0.27 |  |
| Macromolecule 12 | 0.35 | 0.730 | 252 | 0.15 | -1.11 | 0.270 | 167 | 0.29 | -1.95 | | 0.052 | 260 | 0.82 | -0.04 | 0.969 | 176 | 0.16 |  |
| Macromolecule 14 | -0.69 | 0.489 | 237 | 0.18 | -1.97 | 0.050 | 169 | 0.99 | -4.84 | | 0.000 | 249 | 6160.32 | -2.30 | 0.023 | 173 | 1.87 |  |
| Macromolecule 17 | -2.49 | 0.013 | 204 | 2.72 | -2.19 | 0.030 | 143 | 1.59 | -1.65 | | 0.101 | 155 | 0.60 | -2.18 | 0.031 | 115 | 1.64 |  |

**1.2. The effect of the biometric predictors of age, sex, weight and height on neurochemical concentration**

Apart from looking at the effect of sex separately, we also assessed the impact of four biometric predictors (age, sex, height and weight) in explaining neurochemical concentration by employing Bayesian model comparison.

The Bayes Factors we present represent how many more likely a given model is compared to a null model that merely contains the intercept. The benefit of the Bayesian method is that it provides strong evidence for or against the null hypotheses. In particular, we compared 15 possible models containing all combinations of the biometric indices: age, sex, weight, height. As we aimed to examine the biometric determinants of each neurochemical separately, we compared all 15 models of a given neurochemical contained the same number of participants. As can be seen below, for some neurochemicals a model with a single predictor was the best (see single predictor models below, in, 1.2.1), whereas for other neurochemicals a model that featured more than one prediction was the best (see multi-predictor models below, 1.2.2). We defined a given biometric index as significant in predicting a particular neurochemical concentration only if the following criteria were met: (1) the best model was at least three times more likely than the null intercept-only model (BF_0_>3), (2) the best model was at least three times more likely than the second-best model if the second-best model was a more simple one (i.e., featuring at least one predictor less), (3) the biometric index was featured in the best model as determined by Bayesian model comparison, and (4) the biometric index uniquely predicted neurochemical concentration even after controlling from the other biometric indices (see below, equation S1).

Neurochemical concentration ~ age + sex + weight + height

(equation S1)

**Supporting information 1.2.1. List of the best models obtained from the Bayesian model comparison for every neurochemical sorted by the type of biometric predictor.** In the cases presented in this table, the best model featured a single predictor. The biometric predictors that meet the criteria for significance are highlighted in bold and a sign is displayed next by which denotes whether the relationship between the given biometric predictor and neurochemical concentration was positive (+) or negative (-). tCr=total creatine (i.e., creatine+phosphocreatine)

| **Single Predictor Models** | | | | | | | |
| --- | --- | --- | --- | --- | --- | --- | --- |
| **Age** | | **Sex** | | **Weight** | | **Height** | |
| **Region Neurochemical** | **BF10** | **Region - Neurochemical** | **BF10** | **Region - Neurochemical** | **BF10** | **Region - Neurochemical** | **BF10** |
| MFG Ascorbate | **1.5E+11 (-)** | MFG Aspartate | 1.1E+00 | MFG Glucose | **1.6E+01 (+)** | MFG Glutamine | 6.9E+02 |
| MFG Creatine+Phosphocreatine (tCr) | 2.3E+01 | MFG Inositol | 3.8E-01 | MFG Glycerophosphocholine | 2.0E+00 |  |  |
| MFG GABA | 2.0E+04 | MFG Macromolecule 17 | **4.2E+00 (-)** | MFG Lactate | 9.5E+00 |  |  |
| MFG Glutamate | **1.2E+15 (-)** | MFG Phosphoethanolamine | 5.5E-01 | MFG Macromolecule 12 | 1.2E+01 |  |  |
| MFG Macromolecule 09 | 6.7E+01 | IPS Glutamine | 3.4E-01 | MFG Macromolecule 14 | 2.1E-01 |  |  |
| MFG Macromolecule 20 | **4.7E+11 (+)** | **IPS Macromolecule 14** | **9.1E+03 (-)** | IPS Aspartate | 1.0E+00 |  |  |
| MFG N-acetylaspartate | **1.4E+10 (+)** | IPS Macromolecule 17 | 6.4E-01 | IPS Glucose | **1.7E+01 (+)** |  |  |
| MFG Taurine | **2.0E+10 (-)** |  |  | IPS Inositol | 3.7E-01 |  |  |
| IPS Ascorbate | **2.6E+12 (-)** |  |  | IPS Lactate | 3.9E-01 |  |  |
| IPS Creatine+Phosphocreatine (tCr) | **7.2E+05 (+)** |  |  |  |  |  |  |
| IPS GABA | **9.8E+07 (+)** |  |  |  |  |  |  |
| IPS Glutamate | **7.1E+18 (-)** |  |  |  |  |  |  |
| IPS Glutathione | 1.7E+02 |  |  |  |  |  |  |
| IPS N-acetylaspartate | 2.9E+05 |  |  |  |  |  |  |
| IPS Phosphoethanolamine | 3.8E+06 |  |  |  |  |  |  |
| IPS Taurine | **8.6E+22 (-)** |  |  |  |  |  |  |

**Supporting information 1.2.2. List of the best models obtained from the Bayesian model comparison for every neurochemical when the best model featured more than a single predictor**. The biometric predictors that meet the criteria for significance are highlighted in bold and a sign is displayed next by which denotes whether the relationship between the given biometric predictor and neurochemical concentration was positive (+) or negative (-).

| **Multi-predictor models** | | | |
| --- | --- | --- | --- |
| Region | Neurochemical | BF10 | Biometric predictors |
| MFG | Glutathione | 3.2E+01 | Sex, Weight |
| MFG | Scyllo-Inositol | 8.6E+00 | **Age (+), Height (-)** |
| IPS | Glycerophosphocholine | 2.5E+04 | **Sex (+)**, **Age (+)** |
| IPS | Scyllo-Inositol | 1.6E+01 | **Age (+)**, Height |
| IPS | MM09 | 2.8E+05 | **Sex (-)**, **Weight (+)** |
| IPS | MM20 | 4.3E+12 | **Sex (-)**, **Age (+), Weight (+)** |
| IPS | MM12 | 7.9E+01 | Sex, **Weight (+)** |

**Supporting Information 2. The average spectrum from each of the five groups separately.** The spectrum thickness corresponds to ±1 standard deviation from the mean, and parts per million, ppm, are plotted in the x-axis. A) MFG 6 year-olds, B) IPS 6 year-olds, C) MFG 10 year-olds, D) IPS 10 year-olds, E) MFG 14 year-olds, F) IPS 14 year-olds, G) MFG 16 year-olds, H) IPS 16 year-olds, I) MFG 18+ year-olds, J) IPS 18+ year-olds.


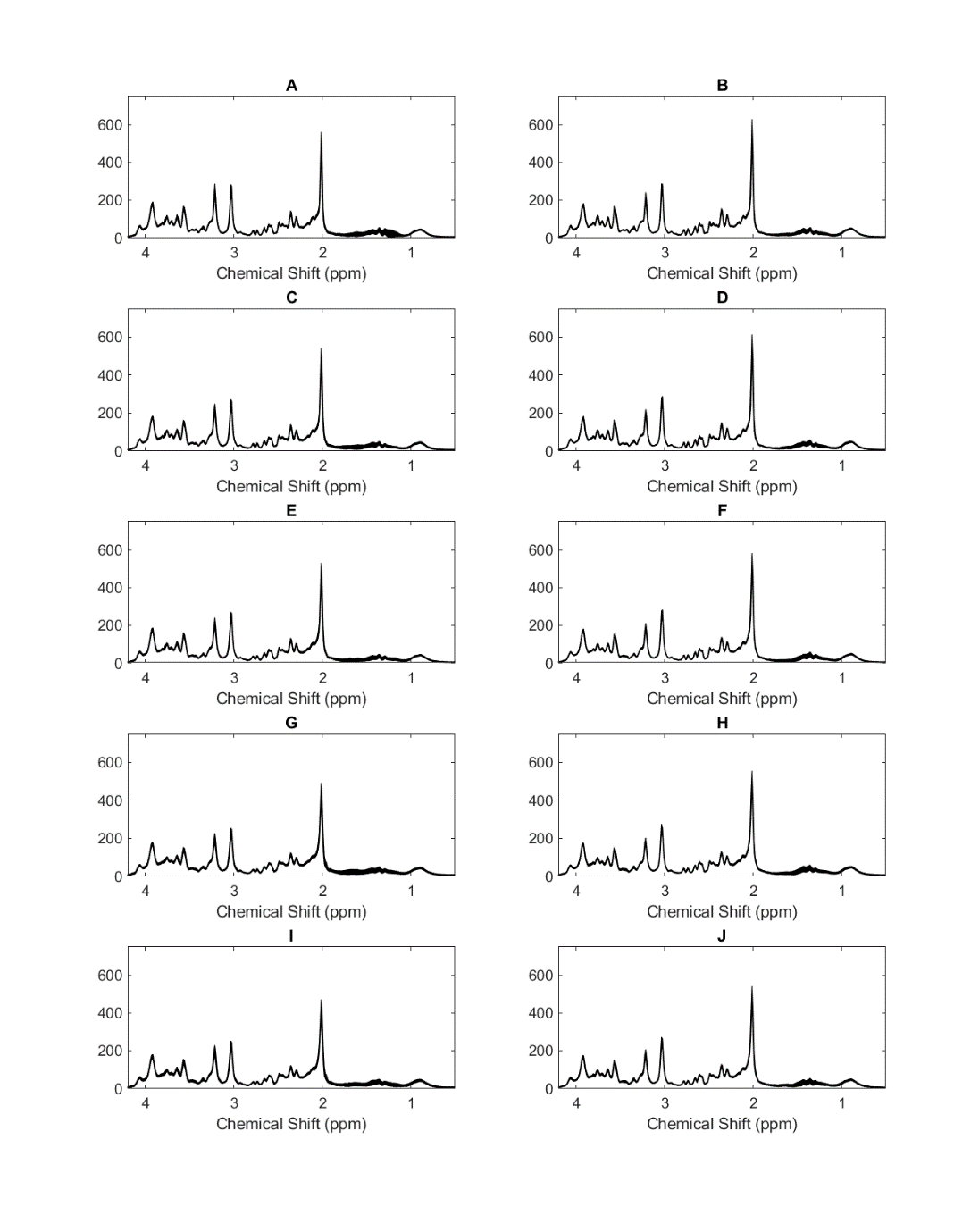


**Supporting Information 3. T2 across development.** A) Histograms of the T2 value distribution of the MFG (A, M=69.28, SD, 3.60, N=268) and the IPS (B, M=70.70, SD=3.32, N=272). Scatterplots depicting the association between age (in years, x-axis) and T2 values (ms, y-axis) for the MFG (C, r_S_=-.731, p<.001) and the IPS (D, r_S_=-.760, p<.001).


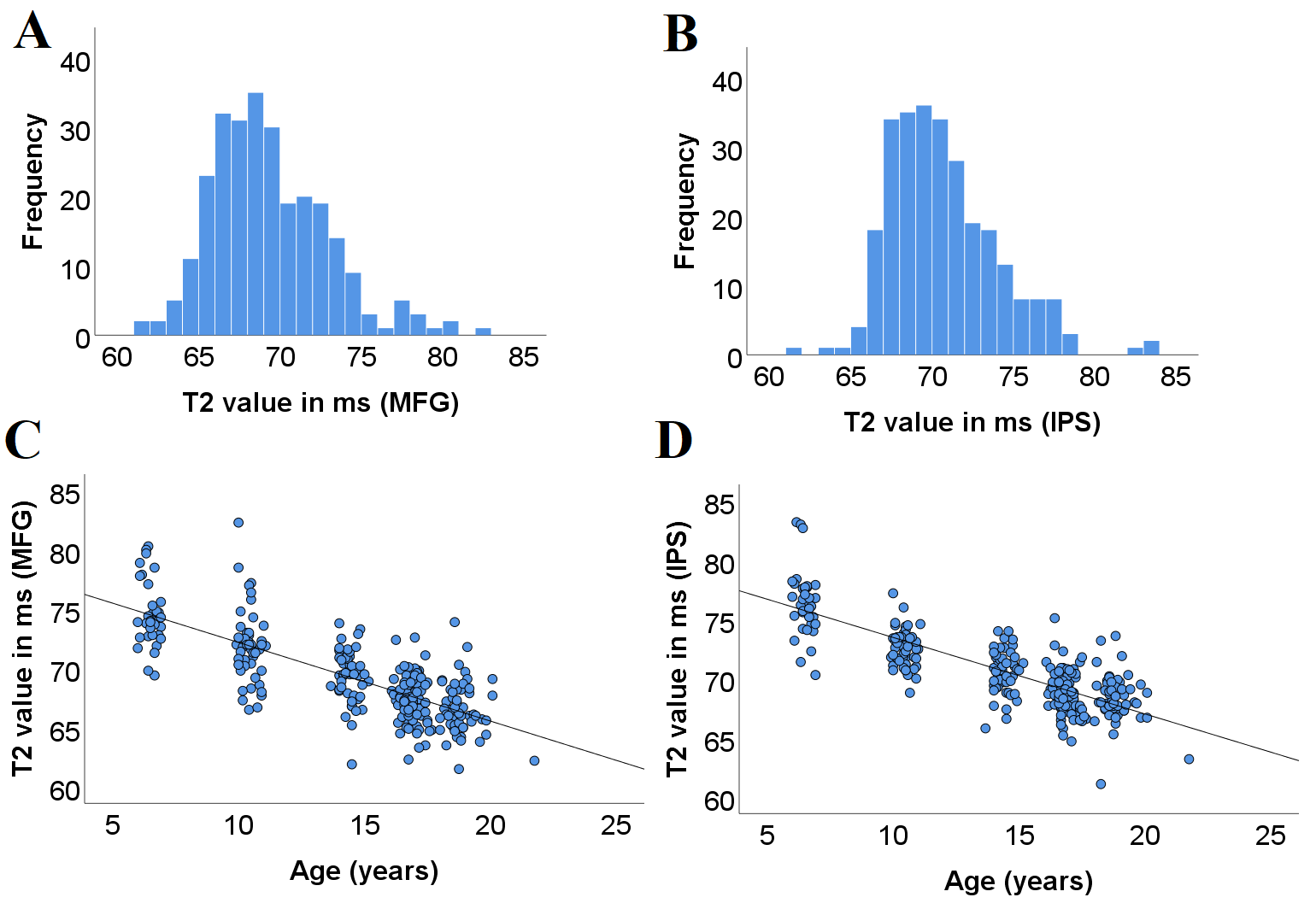


**Supporting Information 4. Spearman’s results from the association between neurochemical concentration and age in each of the four quantification methods.** A1=first assessment, A2= second assessment, r_S_=Spearman’s correlation coefficient, p=uncorrected p-value. tCr=total creatine (i.e., creatine+phosphocreatine)

**Supporting Information 4.1:** quantification method (tissue-corrected concentration, equation 1)

|  | **MFG** | | | | | | **IPS** | | | | | | |
| --- | --- | --- | --- | --- | --- | --- | --- | --- | --- | --- | --- | --- | --- |
|  | **A1** | **A1** | **A2** | **A2** | **A1** | **A2** | | **A1** | **A1** | **A2** | **A2** | **A1** | **A2** |
|  | **r_S_** | **p** | **r_S_** | **p** | **N** | **N** | | **r_S_** | **p** | **r_S_** | **p** | **N** | **N** |
| GABA | 0.27 | 1.0E-05 | 0.35 | 1.8E-06 | 257 | 177 | | 0.35 | 4.4E-09 | 0.37 | 1.8E-07 | 266 | 185 |
| Glutamate | -0.51 | 6.9E-19 | -0.51 | 4.5E-13 | 263 | 178 | | -0.52 | 4.9E-20 | -0.57 | 4.2E-17 | 266 | 185 |
| Glutamine | 0.25 | 3.4E-05 | 0.08 | 3.0E-01 | 262 | 179 | | -0.03 | 5.8E-01 | -0.18 | 1.5E-02 | 265 | 184 |
| Aspartate | -0.08 | 2.1E-01 | -0.07 | 3.8E-01 | 262 | 178 | | -0.08 | 1.9E-01 | -0.20 | 6.9E-03 | 267 | 185 |
| Ascorbate | -0.45 | 4.2E-14 | -0.34 | 4.0E-06 | 253 | 174 | | -0.42 | 1.8E-12 | -0.42 | 4.4E-09 | 265 | 181 |
| Glucose | 0.19 | 1.9E-03 | 0.11 | 1.6E-01 | 263 | 179 | | 0.08 | 1.9E-01 | 0.13 | 8.5E-02 | 263 | 183 |
| Glycerophosphocholine | -0.09 | 1.5E-01 | -0.12 | 1.2E-01 | 263 | 179 | | 0.26 | 2.6E-05 | 0.31 | 2.4E-05 | 265 | 185 |
| Glutathione | -0.18 | 3.0E-03 | -0.20 | 8.0E-03 | 262 | 179 | | -0.20 | 1.1E-03 | -0.28 | 1.3E-04 | 266 | 185 |
| Inositol | 0.01 | 8.6E-01 | 0.20 | 8.2E-03 | 264 | 179 | | 0.05 | 3.9E-01 | 0.19 | 7.9E-03 | 266 | 185 |
| Scyllo-Inositol | 0.11 | 7.6E-02 | 0.35 | 1.8E-06 | 256 | 173 | | 0.16 | 1.2E-02 | 0.30 | 4.4E-05 | 257 | 182 |
| Lactate | 0.15 | 3.5E-02 | 0.04 | 6.6E-01 | 198 | 131 | | 0.06 | 3.7E-01 | 0.14 | 9.4E-02 | 199 | 136 |
| Phosphoethanolamine | 0.02 | 7.5E-01 | 0.02 | 8.3E-01 | 262 | 177 | | -0.34 | 1.5E-08 | -0.30 | 4.6E-05 | 267 | 183 |
| N-acetylaspartate | 0.35 | 4.0E-09 | 0.36 | 5.9E-07 | 262 | 178 | | 0.25 | 2.8E-05 | 0.07 | 3.6E-01 | 267 | 185 |
| Taurine | -0.63 | 2.6E-14 | -0.43 | 8.1E-05 | 116 | 78 | | -0.72 | 2.6E-26 | -0.58 | 1.5E-10 | 155 | 103 |
| tCr | 0.17 | 4.9E-03 | 0.35 | 2.1E-06 | 262 | 178 | | 0.32 | 1.2E-07 | 0.34 | 3.1E-06 | 266 | 184 |
| Macromolecule 09 | 0.23 | 2.3E-04 | 0.15 | 5.1E-02 | 261 | 180 | | 0.23 | 2.0E-04 | 0.19 | 1.2E-02 | 264 | 182 |
| Macromolecule 20 | 0.46 | 3.4E-15 | 0.36 | 9.8E-07 | 261 | 179 | | 0.42 | 1.8E-12 | 0.36 | 3.9E-07 | 265 | 185 |
| Macromolecule 12 | 0.13 | 4.4E-02 | 0.22 | 3.6E-03 | 254 | 170 | | 0.15 | 1.2E-02 | 0.12 | 1.2E-01 | 262 | 178 |
| Macromolecule 14 | -0.01 | 8.4E-01 | -0.15 | 4.4E-02 | 239 | 172 | | -0.05 | 4.5E-01 | -0.17 | 2.3E-02 | 251 | 175 |
| Macromolecule 17 | 0.11 | 1.3E-01 | 0.31 | 1.9E-04 | 206 | 145 | | 0.09 | 2.4E-01 | -0.05 | 5.8E-01 | 157 | 117 |

**Supporting Information 4.2:** quantification method (absolute concentration)

|  | **MFG** | | | | | | **IPS** | | | | | | | |
| --- | --- | --- | --- | --- | --- | --- | --- | --- | --- | --- | --- | --- | --- | --- |
|  | **A1** | **A1** | **A2** | **A2** | **A1** | **A2** | | **A1** | **A1** | **A2** | **A2** | **A1** | **A2** |  |
|  | **r_S_** | **p** | **r_S_** | **p** | **N** | **N** | | **r_S_** | **p** | **r_S_** | **p** | **N** | **N** |  |
| GABA | 0.29 | 3.4E-06 | 0.34 | 3.7E-06 | 257 | 177 | | 0.30 | 4.6E-07 | 0.35 | 9.8E-07 | 266 | 185 |  |
| Glutamate | -0.56 | 2.6E-23 | -0.60 | 1.3E-18 | 263 | 178 | | -0.72 | 1.8E-43 | -0.73 | 1.2E-31 | 266 | 185 |  |
| Glutamine | 0.26 | 1.6E-05 | 0.04 | 5.8E-01 | 262 | 179 | | -0.14 | 2.1E-02 | -0.28 | 1.6E-04 | 264 | 183 |  |
| Aspartate | -0.07 | 2.3E-01 | -0.10 | 1.7E-01 | 262 | 178 | | -0.21 | 5.8E-04 | -0.28 | 9.3E-05 | 266 | 185 |  |
| Ascorbate | -0.45 | 7.4E-14 | -0.35 | 1.6E-06 | 253 | 174 | | -0.49 | 1.9E-17 | -0.46 | 4.7E-11 | 264 | 181 |  |
| Glucose | 0.19 | 2.1E-03 | 0.08 | 2.6E-01 | 263 | 179 | | 0.02 | 7.3E-01 | 0.09 | 2.1E-01 | 265 | 183 |  |
| Glycerophosphocholine | -0.07 | 2.3E-01 | -0.14 | 5.8E-02 | 262 | 179 | | 0.13 | 2.9E-02 | 0.24 | 1.1E-03 | 265 | 184 |  |
| Glutathione | -0.18 | 3.7E-03 | -0.24 | 1.3E-03 | 262 | 179 | | -0.37 | 2.9E-10 | -0.41 | 5.0E-09 | 266 | 184 |  |
| Inositol | 0.01 | 9.2E-01 | 0.12 | 1.1E-01 | 264 | 179 | | -0.08 | 1.9E-01 | 0.10 | 1.9E-01 | 265 | 185 |  |
| Scyllo-Inositol | 0.12 | 5.9E-02 | 0.34 | 4.9E-06 | 257 | 173 | | 0.12 | 5.9E-02 | 0.28 | 1.6E-04 | 257 | 182 |  |
| Lactate | 0.15 | 2.9E-02 | 0.01 | 9.5E-01 | 198 | 131 | | 0.00 | 9.8E-01 | 0.11 | 2.1E-01 | 198 | 136 |  |
| Phosphoethanolamine | 0.04 | 5.2E-01 | -0.03 | 6.8E-01 | 260 | 177 | | -0.45 | 5.1E-15 | -0.39 | 3.7E-08 | 267 | 184 |  |
| N-acetylaspartate | 0.41 | 4.4E-12 | 0.32 | 1.8E-05 | 261 | 177 | | 0.08 | 1.8E-01 | -0.08 | 2.8E-01 | 265 | 185 |  |
| Taurine | -0.65 | 2.2E-15 | -0.48 | 9.6E-06 | 116 | 78 | | -0.78 | 1.1E-32 | -0.64 | 3.9E-13 | 155 | 103 |  |
| tCr | 0.22 | 3.6E-04 | 0.31 | 2.6E-05 | 262 | 178 | | 0.21 | 4.8E-04 | 0.25 | 6.2E-04 | 266 | 185 |  |
| Macromolecule 09 | 0.23 | 1.3E-04 | 0.12 | 1.1E-01 | 261 | 180 | | 0.14 | 2.4E-02 | 0.15 | 4.6E-02 | 264 | 182 |  |
| Macromolecule 20 | 0.48 | 1.7E-16 | 0.34 | 2.3E-06 | 259 | 179 | | 0.37 | 6.2E-10 | 0.34 | 2.4E-06 | 265 | 184 |  |
| Macromolecule 12 | 0.13 | 3.6E-02 | 0.21 | 6.6E-03 | 254 | 170 | | 0.10 | 1.0E-01 | 0.10 | 1.9E-01 | 261 | 178 |  |
| Macromolecule 14 | -0.01 | 8.6E-01 | -0.17 | 2.7E-02 | 239 | 172 | | -0.08 | 2.3E-01 | -0.18 | 1.7E-02 | 250 | 175 |  |
| Macromolecule 17 | 0.10 | 1.4E-01 | 0.30 | 2.5E-04 | 206 | 145 | | 0.06 | 4.6E-01 | -0.08 | 4.1E-01 | 157 | 117 |  |

**Supporting Information 4.3:** quantification method (absolute concentration divided by total creation, creatine+phosphocreatine)

|  | **MFG** | | | | | | **IPS** | | | | | | |  |
| --- | --- | --- | --- | --- | --- | --- | --- | --- | --- | --- | --- | --- | --- | --- |
|  | **A1** | **A1** | **A2** | **A2** | **A1** | **A2** | | **A1** | **A1** | **A2** | **A2** | **A1** | **A2** | |
|  | **r_S_** | **p** | **r_S_** | **p** | **N** | **N** | | **r_S_** | **p** | **r_S_** | **p** | **N** | **N** | |
| GABA | 0.26 | 2.8E-05 | 0.29 | 7.6E-05 | 255 | 177 | | 0.25 | 3.5E-05 | 0.27 | 1.6E-04 | 265 | 185 | |
| Glutamate | -0.60 | 3.1E-27 | -0.65 | 1.9E-22 | 263 | 179 | | -0.72 | 1.4E-43 | -0.76 | 2.0E-35 | 267 | 184 | |
| Glutamine | 0.17 | 5.6E-03 | -0.03 | 7.4E-01 | 260 | 178 | | -0.20 | 1.1E-03 | -0.32 | 7.5E-06 | 265 | 185 | |
| Aspartate | -0.15 | 1.5E-02 | -0.19 | 1.2E-02 | 262 | 177 | | -0.28 | 5.0E-06 | -0.36 | 4.0E-07 | 267 | 185 | |
| Ascorbate | -0.49 | 2.3E-16 | -0.39 | 1.5E-07 | 253 | 174 | | -0.51 | 1.3E-18 | -0.49 | 3.2E-12 | 265 | 182 | |
| Glucose | 0.15 | 1.9E-02 | 0.04 | 6.1E-01 | 262 | 179 | | -0.01 | 8.8E-01 | 0.04 | 5.6E-01 | 265 | 183 | |
| Glycerophosphocholine | -0.16 | 1.1E-02 | -0.21 | 5.2E-03 | 262 | 180 | | 0.04 | 4.7E-01 | 0.13 | 8.7E-02 | 266 | 184 | |
| Glutathione | -0.29 | 2.4E-06 | -0.33 | 6.1E-06 | 262 | 178 | | -0.42 | 9.7E-13 | -0.52 | 3.1E-14 | 267 | 185 | |
| Inositol | -0.13 | 3.3E-02 | 0.00 | 9.7E-01 | 264 | 179 | | -0.23 | 2.1E-04 | -0.06 | 4.3E-01 | 266 | 185 | |
| Scyllo-Inositol | 0.09 | 1.5E-01 | 0.31 | 3.5E-05 | 257 | 173 | | 0.08 | 1.8E-01 | 0.26 | 3.8E-04 | 257 | 181 | |
| Lactate | 0.13 | 7.8E-02 | -0.03 | 7.1E-01 | 197 | 131 | | -0.05 | 4.7E-01 | 0.07 | 4.0E-01 | 198 | 136 | |
| Phosphoethanolamine | -0.04 | 5.6E-01 | -0.13 | 9.4E-02 | 258 | 178 | | -0.49 | 9.0E-18 | -0.47 | 2.3E-11 | 266 | 184 | |
| N-acetylaspartate | 0.22 | 4.0E-04 | 0.07 | 3.3E-01 | 264 | 178 | | -0.12 | 6.0E-02 | -0.30 | 3.2E-05 | 266 | 185 | |
| Taurine | -0.70 | 2.2E-18 | -0.49 | 5.1E-06 | 116 | 78 | | -0.80 | 2.6E-36 | -0.67 | 1.2E-14 | 155 | 103 | |
| tCr | ΝΑ | ΝΑ | ΝΑ | ΝΑ | ΝΑ | ΝΑ | | ΝΑ | ΝΑ | ΝΑ | ΝΑ | ΝΑ | ΝΑ | |
| Macromolecule 09 | 0.18 | 3.7E-03 | 0.03 | 6.5E-01 | 260 | 180 | | 0.07 | 2.3E-01 | 0.07 | 3.2E-01 | 264 | 183 | |
| Macromolecule 20 | 0.43 | 7.5E-13 | 0.29 | 8.5E-05 | 259 | 179 | | 0.31 | 1.8E-07 | 0.25 | 7.1E-04 | 265 | 184 | |
| Macromolecule 12 | 0.10 | 1.1E-01 | 0.18 | 1.9E-02 | 254 | 169 | | 0.07 | 2.6E-01 | 0.06 | 3.9E-01 | 261 | 178 | |
| Macromolecule 14 | -0.04 | 5.7E-01 | -0.20 | 7.4E-03 | 239 | 172 | | -0.10 | 1.0E-01 | -0.22 | 4.0E-03 | 250 | 175 | |
| Macromolecule 17 | 0.09 | 2.2E-01 | 0.26 | 1.8E-03 | 206 | 144 | | 0.03 | 6.7E-01 | -0.10 | 2.8E-01 | 157 | 117 | |

**Supporting Information 4.4:** quantification method (T2-corrected concentration, equation 2)

|  | **MFG** | | | | | **IPS** | | | | | | |  |
| --- | --- | --- | --- | --- | --- | --- | --- | --- | --- | --- | --- | --- | --- |
|  | **A1** | **A1** | **A2** | **A2** | **A1** | **A2** | **A1** | **A1** | **A2** | **A2** | **A1** | **A2** | |
|  | **r_S_** | **p** | **r_S_** | **p** | **N** | **N** | **r_S_** | **p** | **r_S_** | **p** | **N** | **N** | |
| GABA | 0.22 | 4.6E-04 | 0.27 | 3.2E-04 | 255 | 176 | 0.26 | 1.4E-05 | 0.28 | 1.4E-04 | 266 | 185 | |
| Glutamate | -0.60 | 1.4E-26 | -0.59 | 7.5E-18 | 260 | 177 | -0.59 | 5.3E-26 | -0.64 | 6.8E-23 | 266 | 185 | |
| Glutamine | 0.13 | 4.3E-02 | -0.03 | 6.4E-01 | 262 | 178 | -0.15 | 1.4E-02 | -0.28 | 1.5E-04 | 265 | 184 | |
| Aspartate | -0.16 | 9.0E-03 | -0.15 | 4.2E-02 | 261 | 177 | -0.20 | 1.2E-03 | -0.32 | 7.6E-06 | 267 | 185 | |
| Ascorbate | -0.51 | 4.8E-18 | -0.38 | 2.6E-07 | 253 | 172 | -0.48 | 2.5E-16 | -0.49 | 2.0E-12 | 265 | 181 | |
| Glucose | 0.12 | 5.2E-02 | 0.05 | 5.2E-01 | 262 | 177 | 0.00 | 9.5E-01 | 0.04 | 5.6E-01 | 263 | 183 | |
| Glycerophosphocholine | -0.24 | 1.0E-04 | -0.23 | 1.8E-03 | 262 | 178 | 0.09 | 1.5E-01 | 0.14 | 5.0E-02 | 265 | 185 | |
| Glutathione | -0.32 | 1.4E-07 | -0.33 | 6.2E-06 | 262 | 178 | -0.32 | 7.2E-08 | -0.42 | 2.1E-09 | 265 | 185 | |
| Inositol | -0.15 | 1.4E-02 | 0.04 | 5.8E-01 | 263 | 178 | -0.12 | 6.0E-02 | 0.01 | 8.6E-01 | 266 | 185 | |
| Scyllo-Inositol | 0.06 | 3.3E-01 | 0.33 | 8.5E-06 | 257 | 172 | 0.11 | 8.0E-02 | 0.26 | 4.8E-04 | 257 | 182 | |
| Lactate | 0.11 | 1.2E-01 | -0.01 | 8.8E-01 | 196 | 130 | -0.01 | 9.0E-01 | 0.08 | 3.8E-01 | 199 | 136 | |
| Phosphoethanolamine | -0.09 | 1.3E-01 | -0.12 | 1.0E-01 | 261 | 177 | -0.44 | 3.6E-14 | -0.42 | 2.0E-09 | 266 | 183 | |
| N-acetylaspartate | 0.07 | 2.6E-01 | 0.06 | 4.4E-01 | 260 | 177 | -0.02 | 7.2E-01 | -0.24 | 9.7E-04 | 266 | 185 | |
| Taurine | -0.64 | 6.6E-15 | -0.46 | 2.8E-05 | 116 | 77 | -0.74 | 2.1E-28 | -0.60 | 1.6E-11 | 155 | 103 | |
| tCr | -0.11 | 6.8E-02 | 0.03 | 6.8E-01 | 261 | 176 | 0.08 | 2.0E-01 | 0.05 | 5.2E-01 | 266 | 184 | |
| Macromolecule 09 | 0.12 | 4.9E-02 | 0.03 | 7.1E-01 | 261 | 179 | 0.12 | 5.7E-02 | 0.06 | 3.9E-01 | 264 | 182 | |
| Macromolecule 20 | 0.39 | 1.3E-10 | 0.26 | 5.2E-04 | 259 | 179 | 0.33 | 3.2E-08 | 0.27 | 2.4E-04 | 265 | 184 | |
| Macromolecule 12 | 0.08 | 2.0E-01 | 0.17 | 3.0E-02 | 254 | 169 | 0.10 | 9.5E-02 | 0.07 | 3.4E-01 | 262 | 178 | |
| Macromolecule 14 | -0.06 | 3.7E-01 | -0.20 | 1.0E-02 | 239 | 171 | -0.09 | 1.4E-01 | -0.22 | 3.9E-03 | 251 | 175 | |
| Macromolecule 17 | 0.08 | 2.3E-01 | 0.27 | 1.1E-03 | 206 | 145 | 0.05 | 5.7E-01 | -0.09 | 3.4E-01 | 157 | 117 | |

**Supporting Information 5**

Scatterplots depicting the association between chronological age (x-axis, years) and neurochemical concentration (y-axis, micromol/g) at the first assessment (A1) or second assessment (A2), with linear (blue), quadratic (red) and cubic (green) fits. The title of each figure denotes the region (MFG, middle frontal gyrus, or IPS: intraparietal sulcus) as well as the quantification method. (Tissue corrected concentration (equation 1), T2 corrected concentration (equation 2), Absolute concentration, tCr Revative concentration=absolute concentration divided by creatine+phosphocreatine.


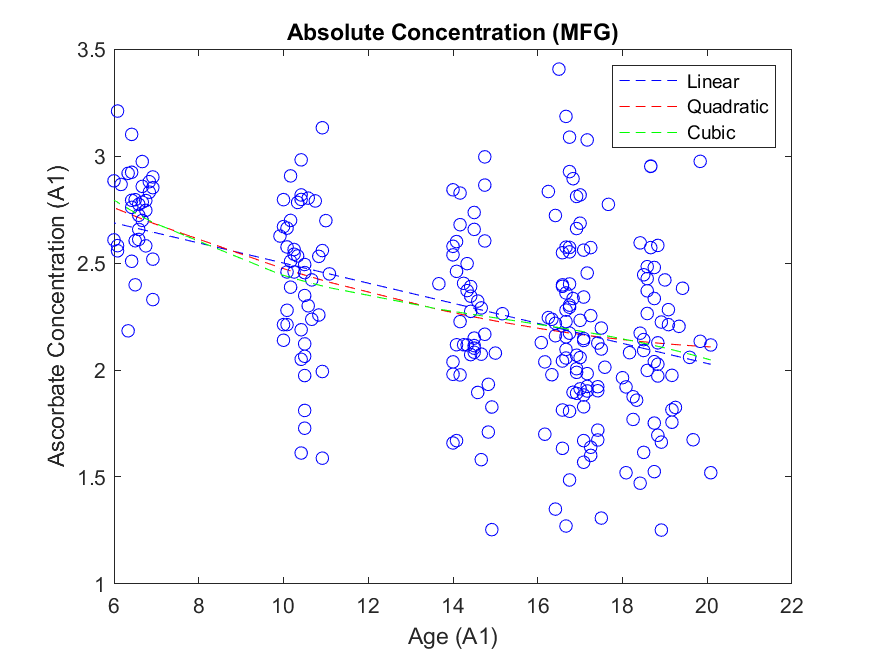
**
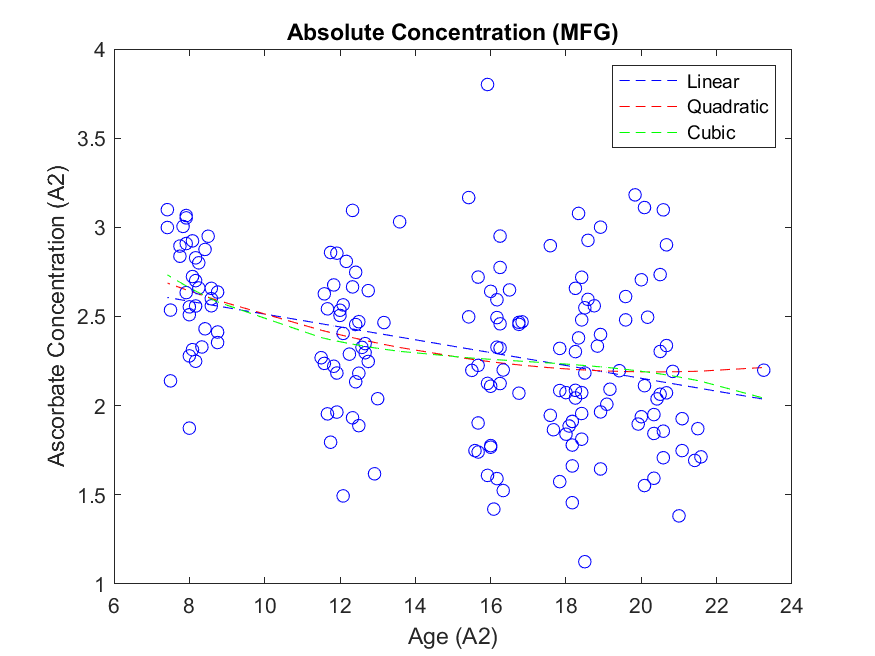

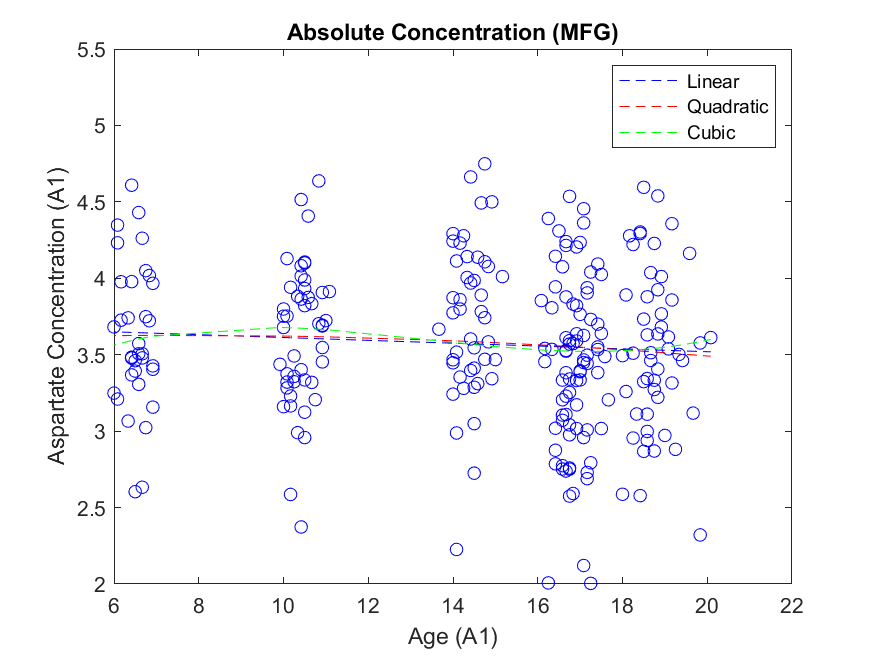

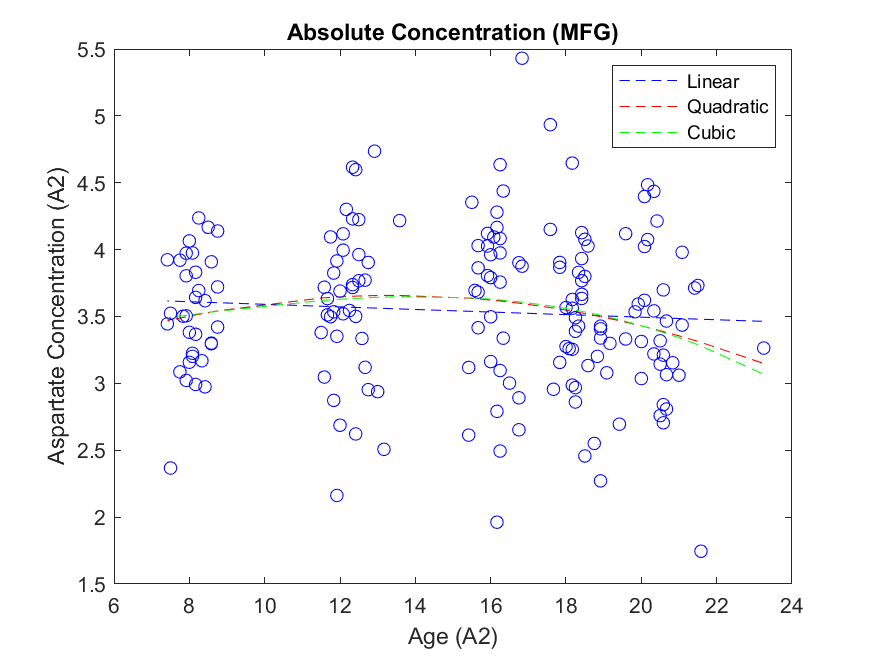

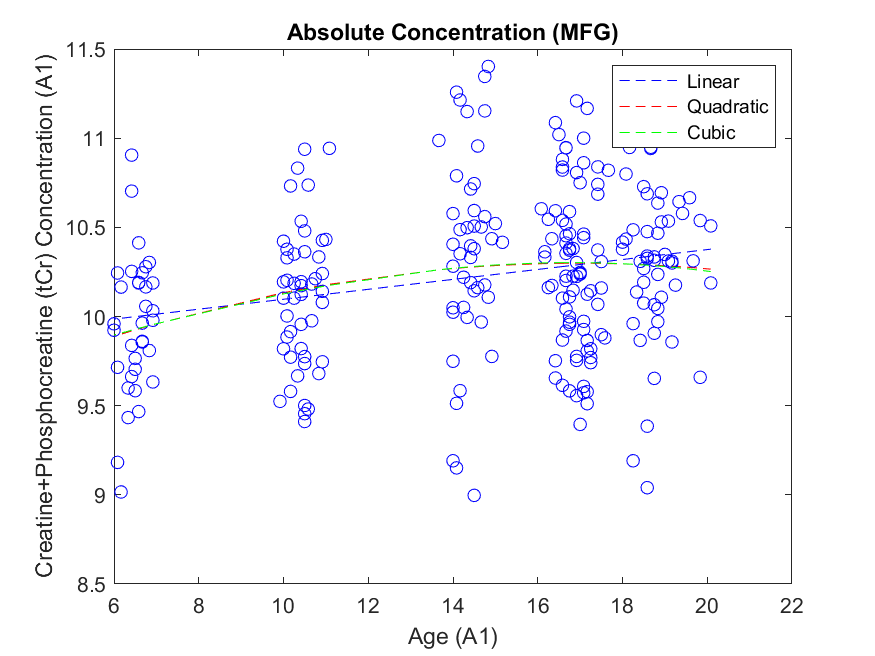

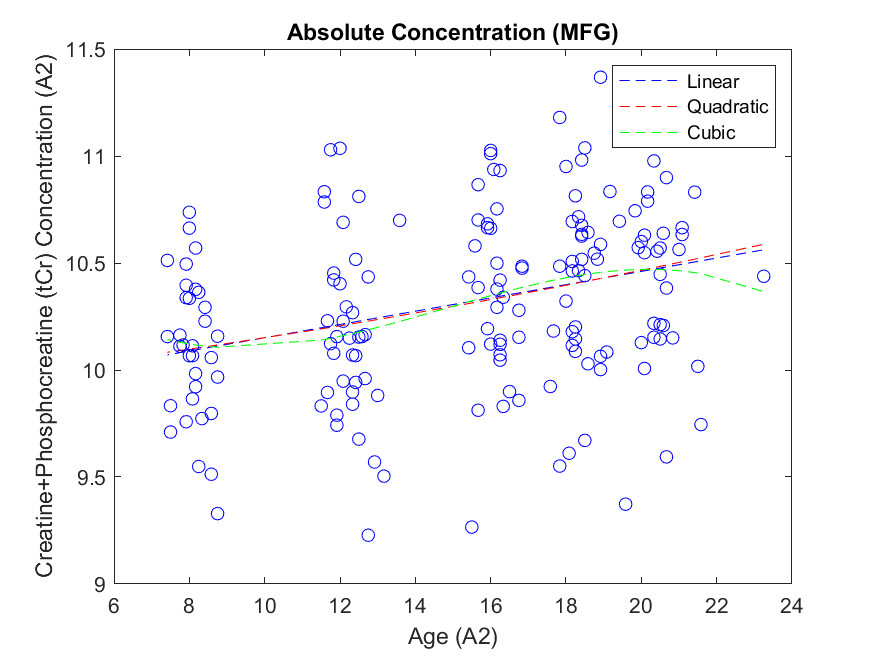

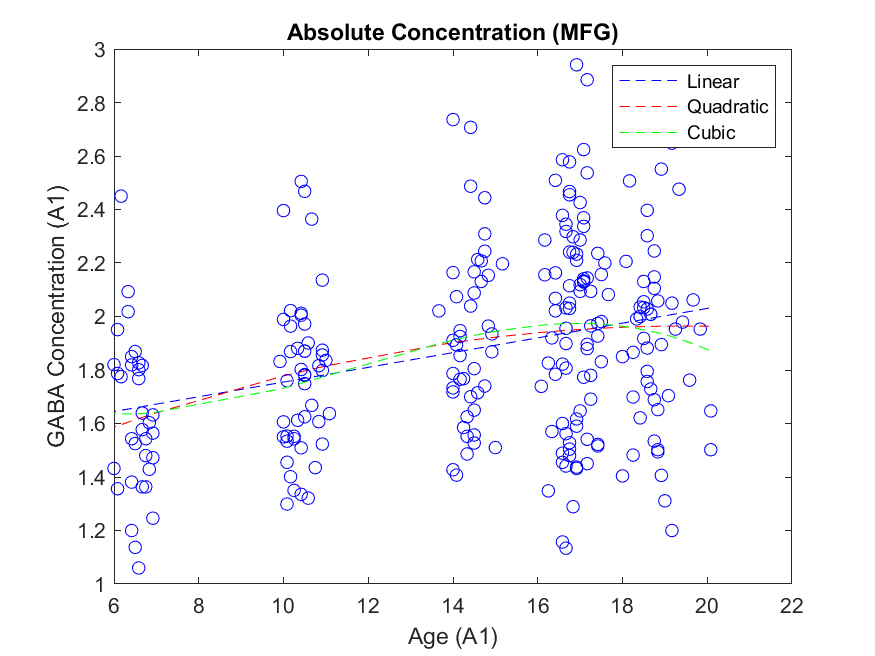

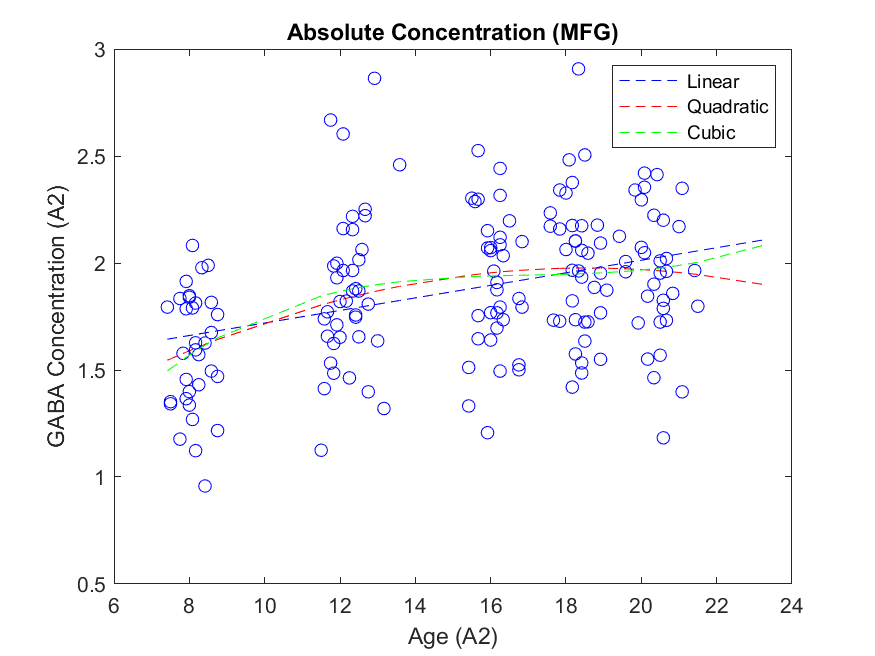

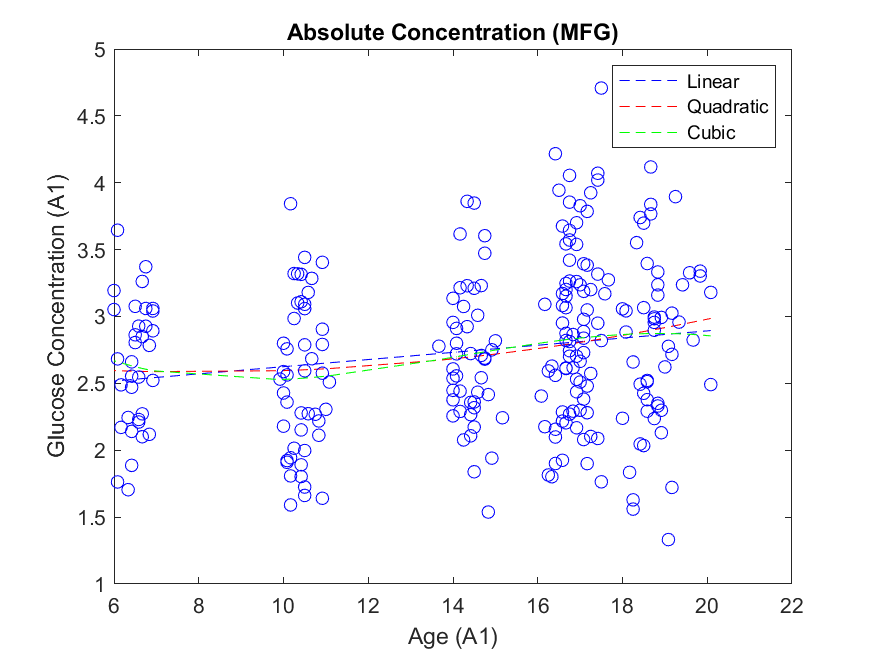

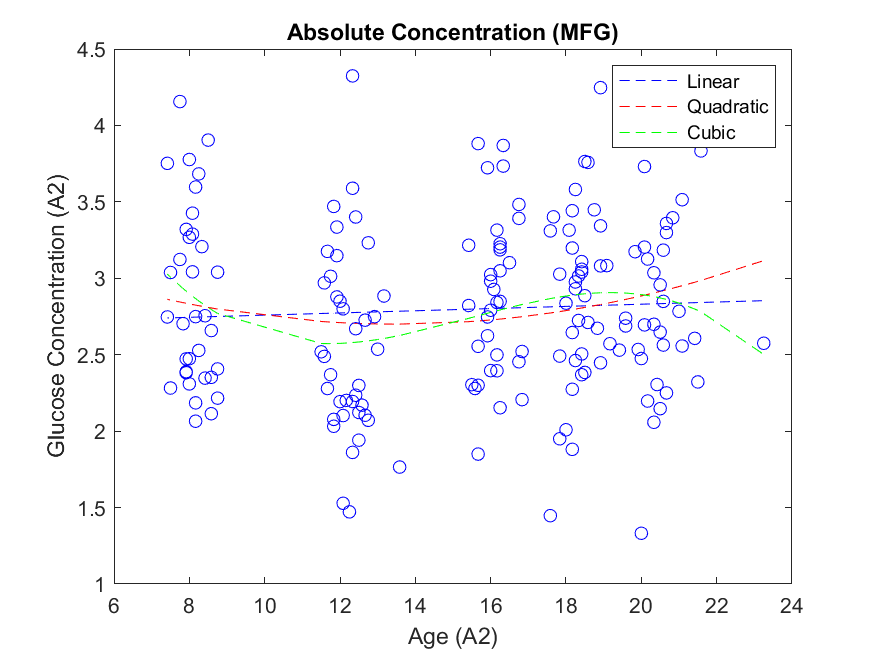

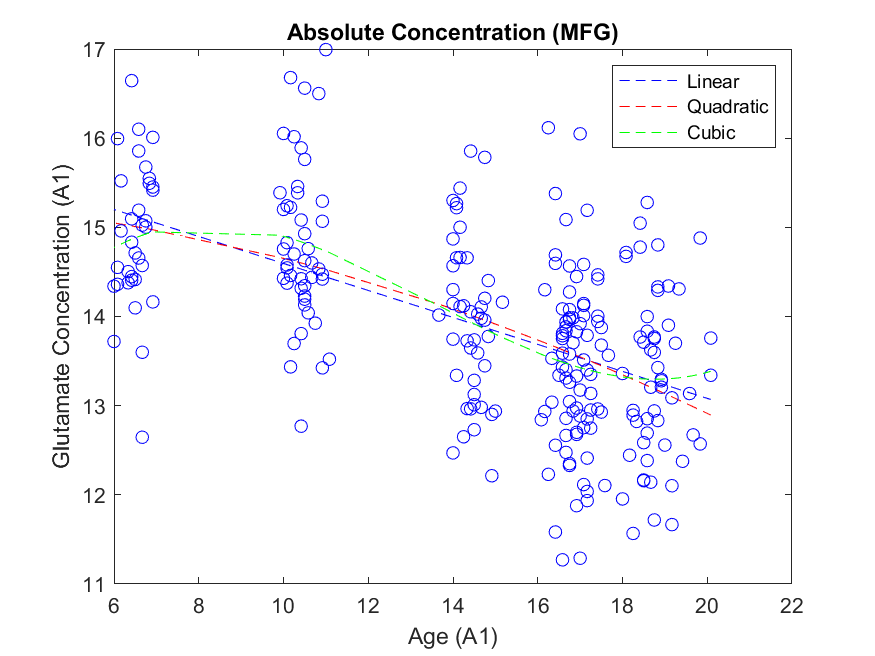

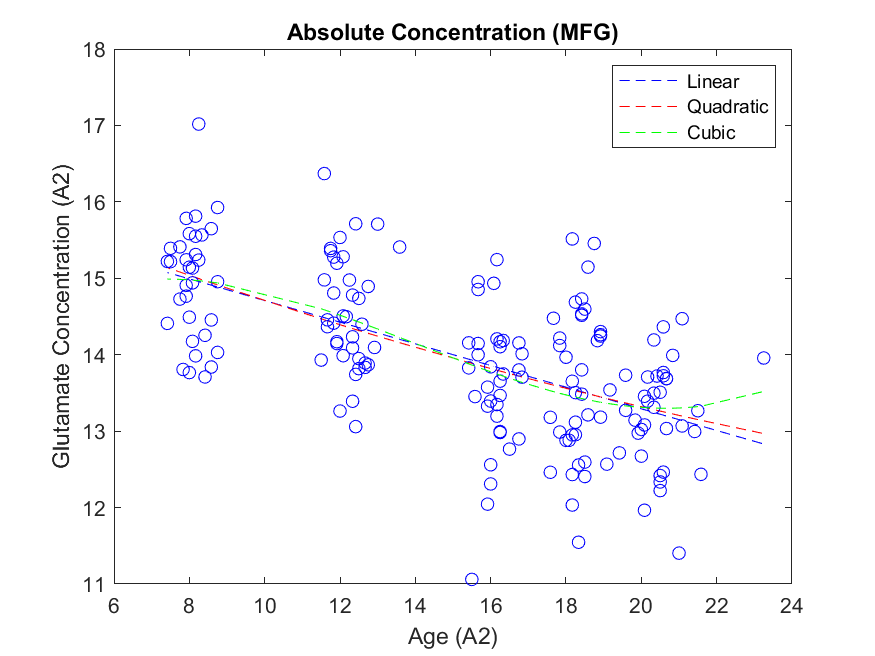

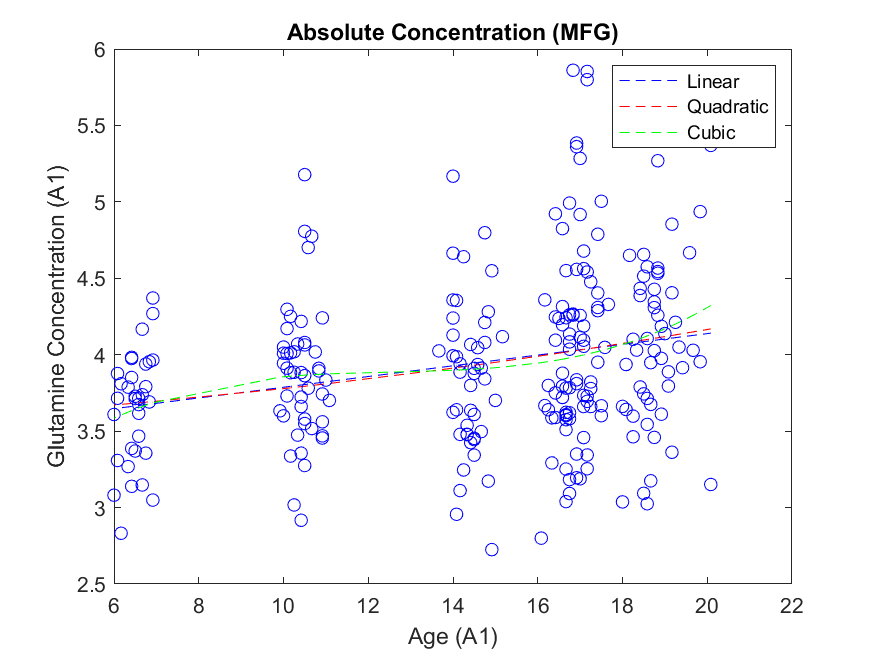

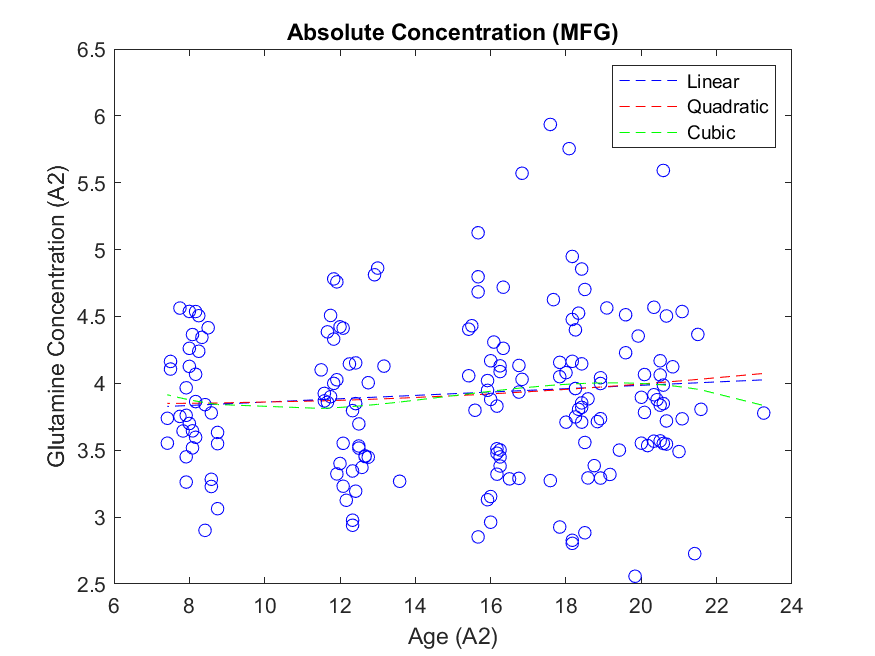

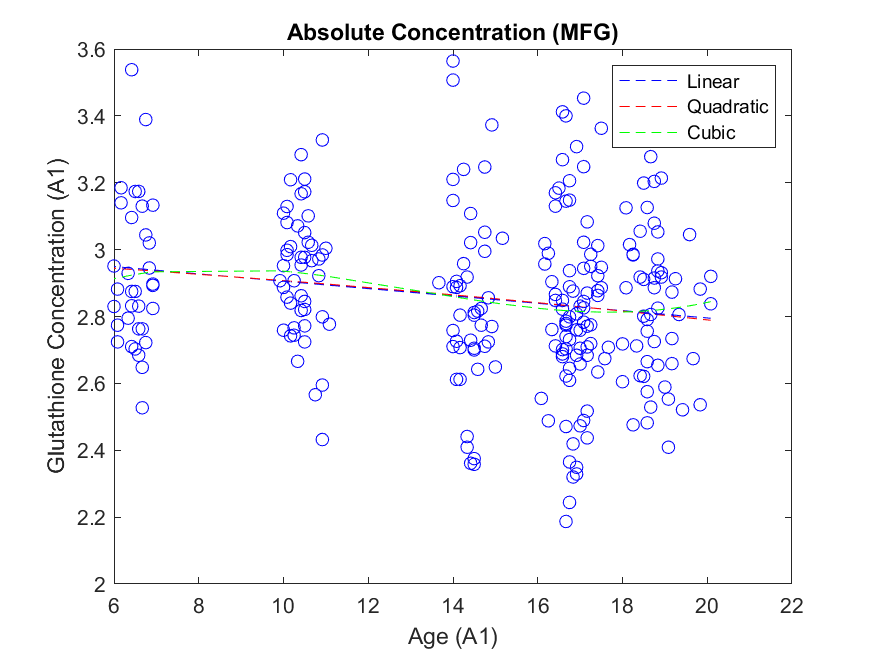

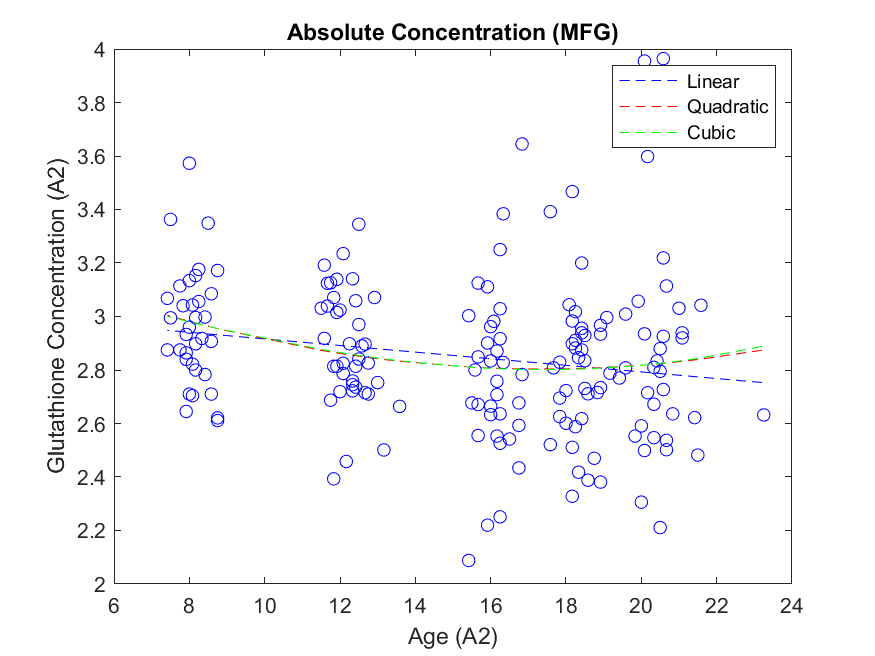

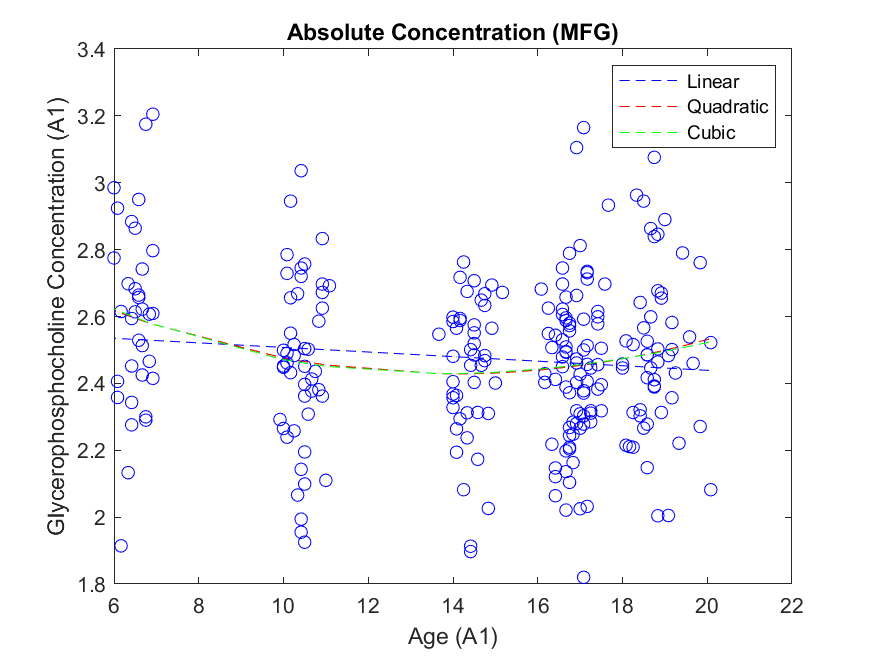

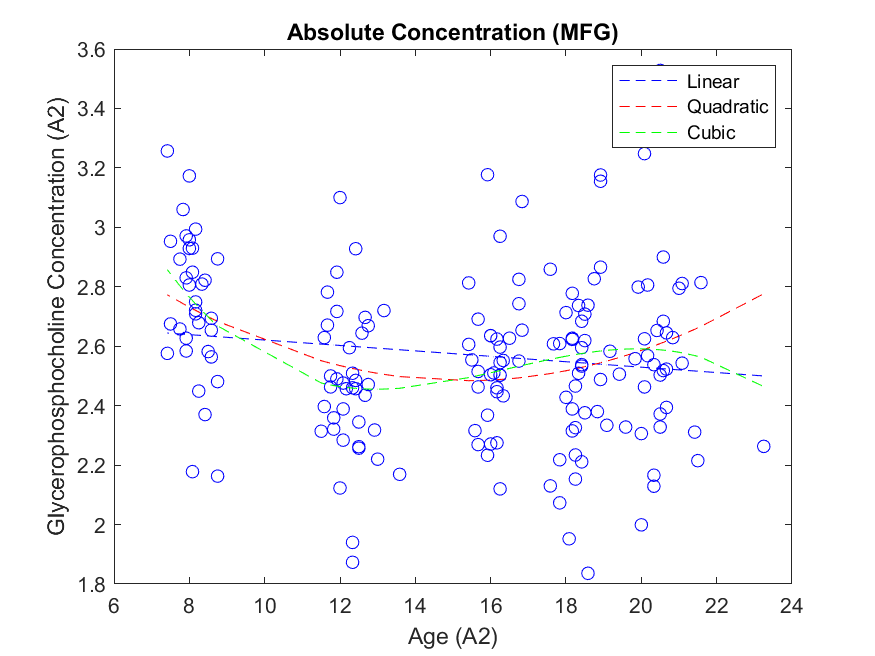

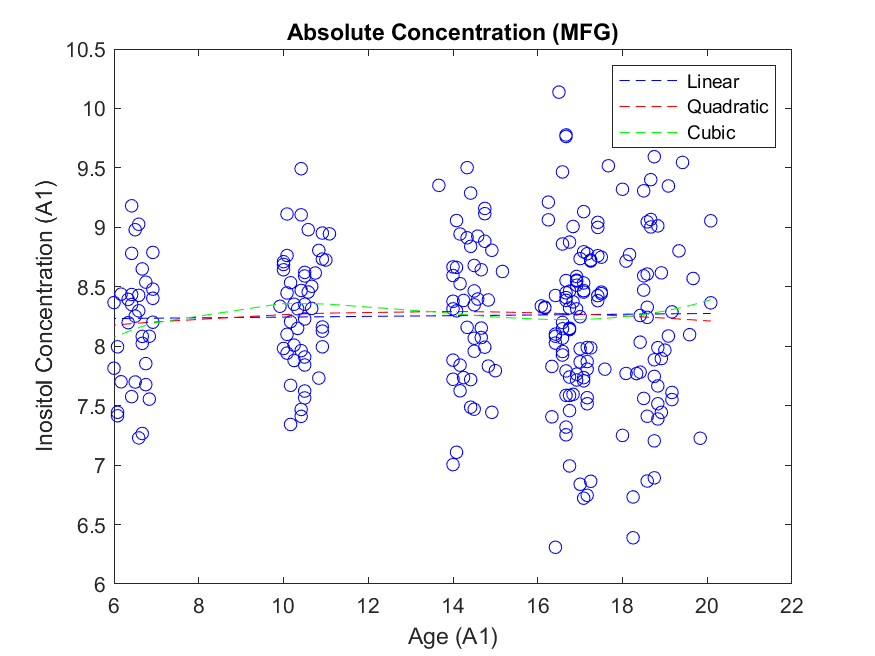

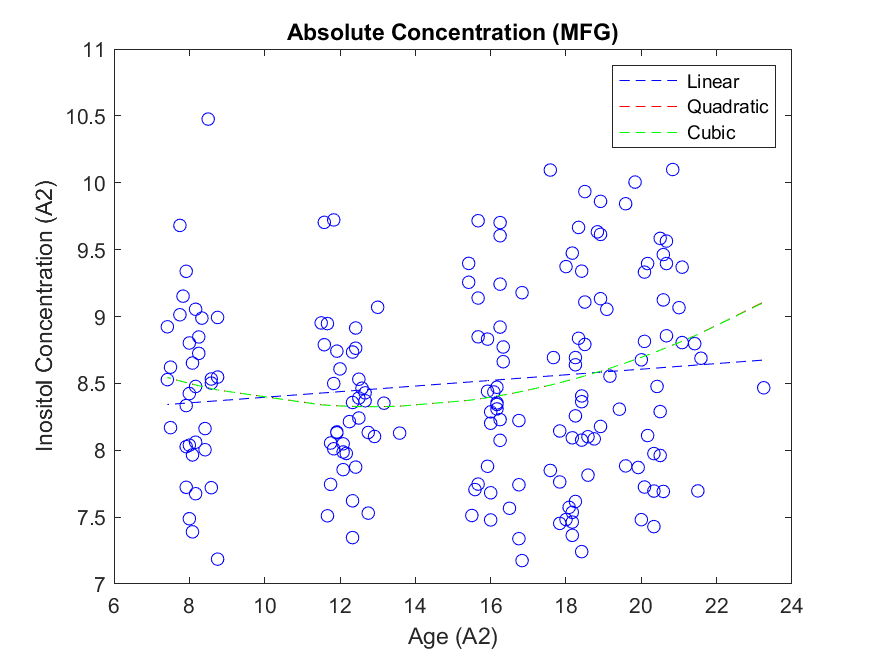

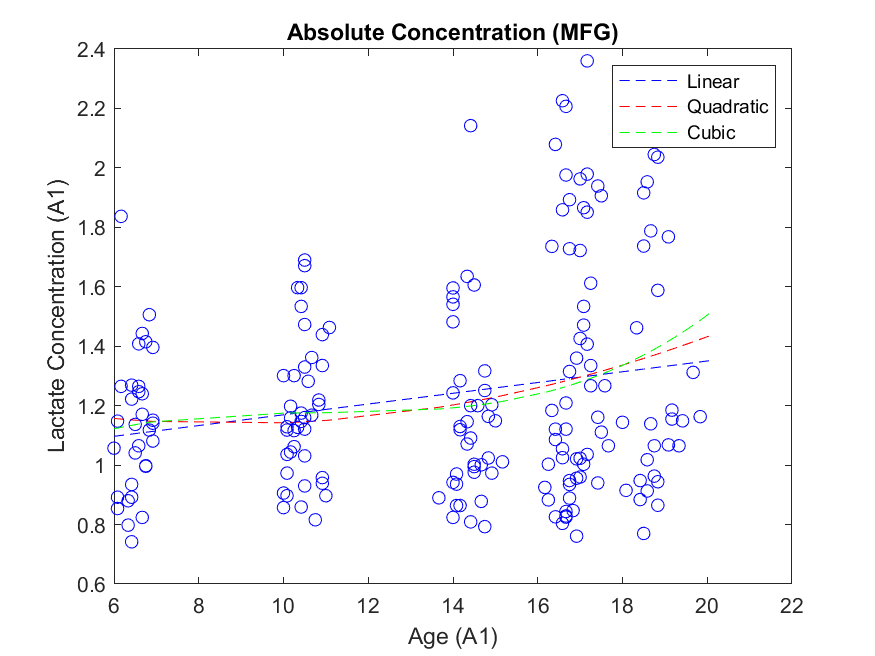

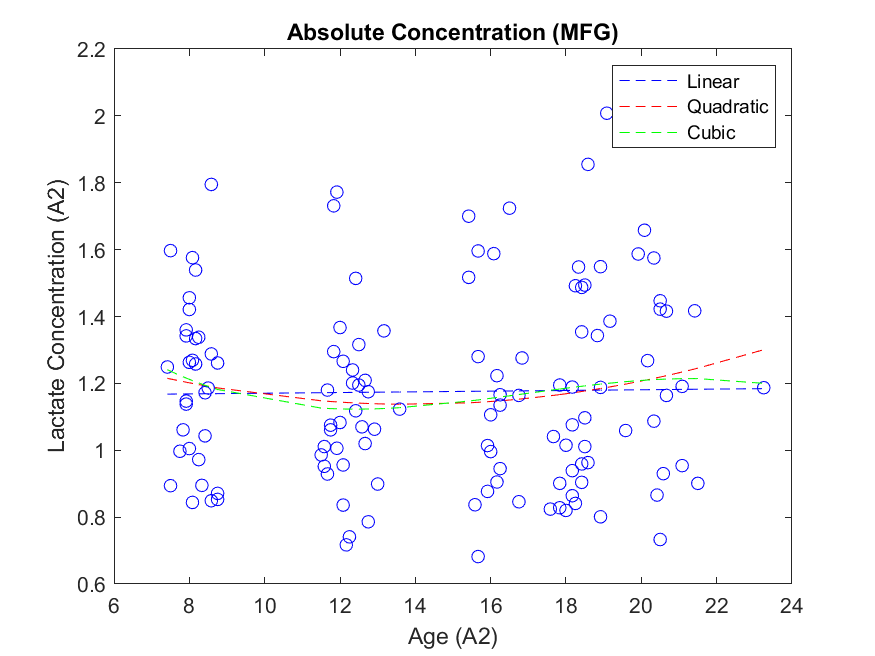

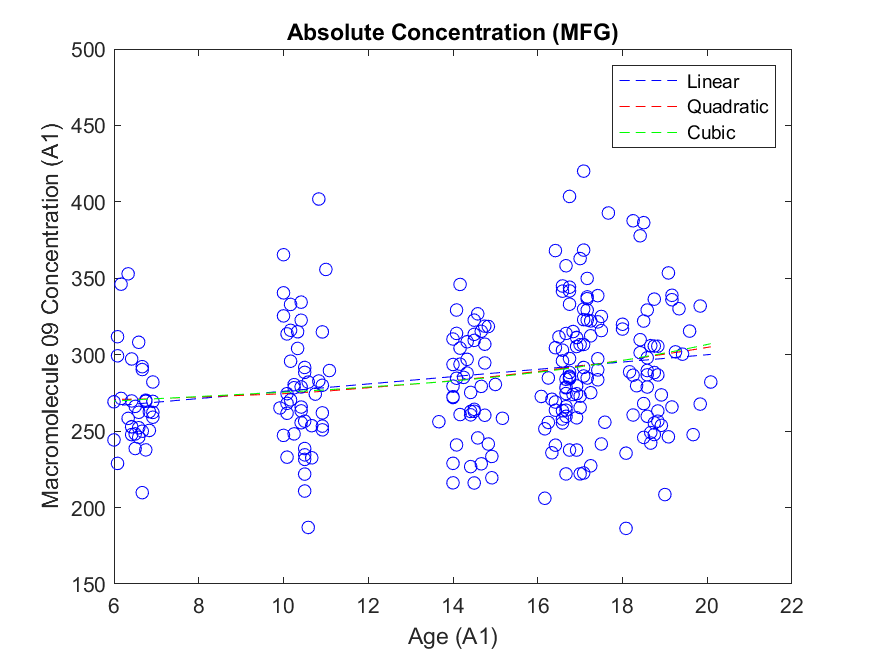

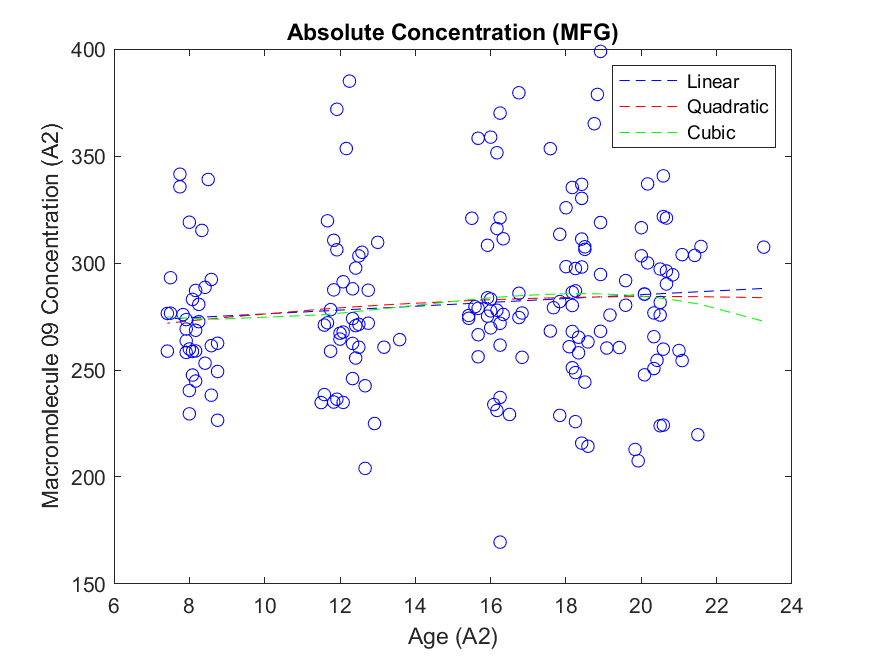

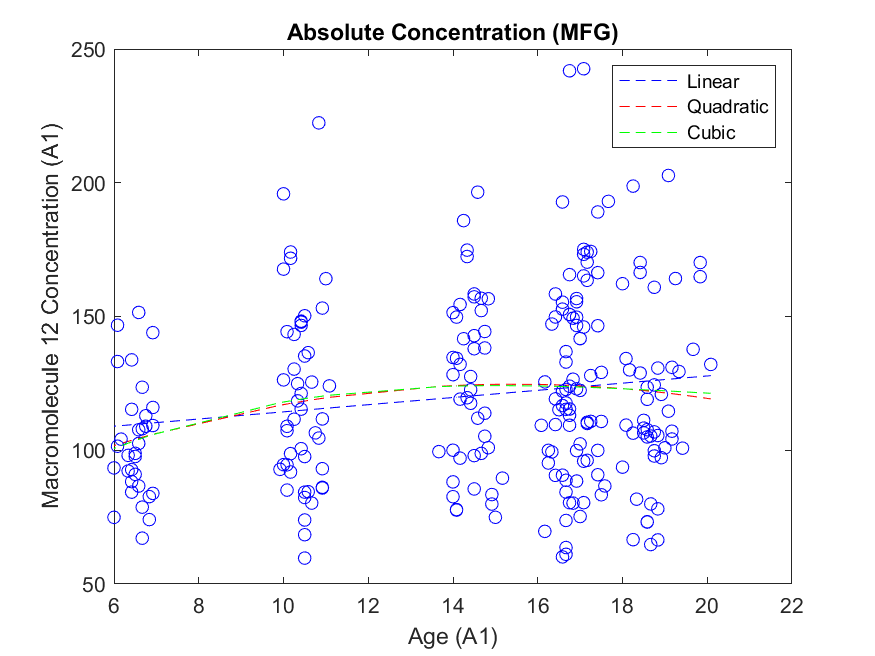

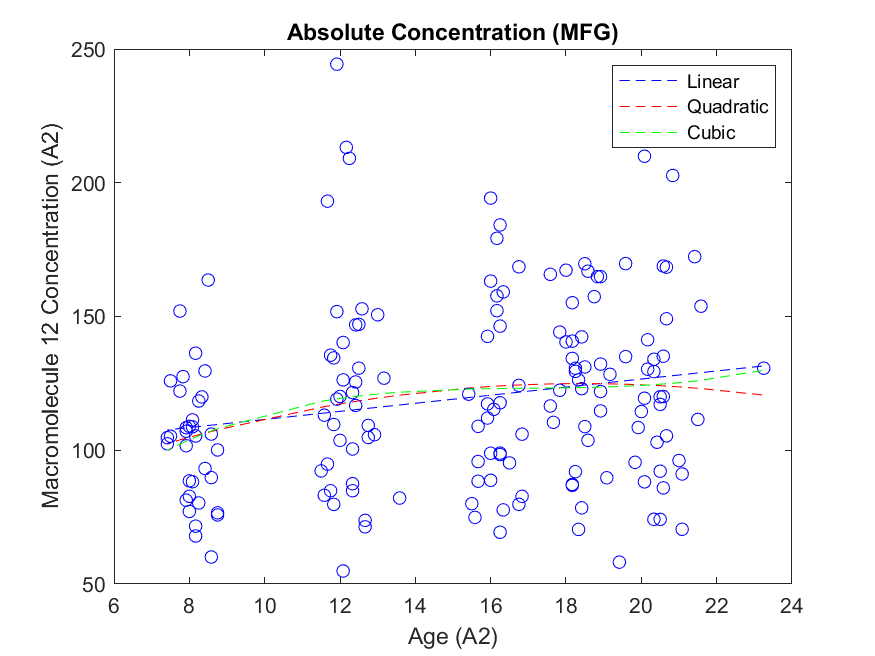

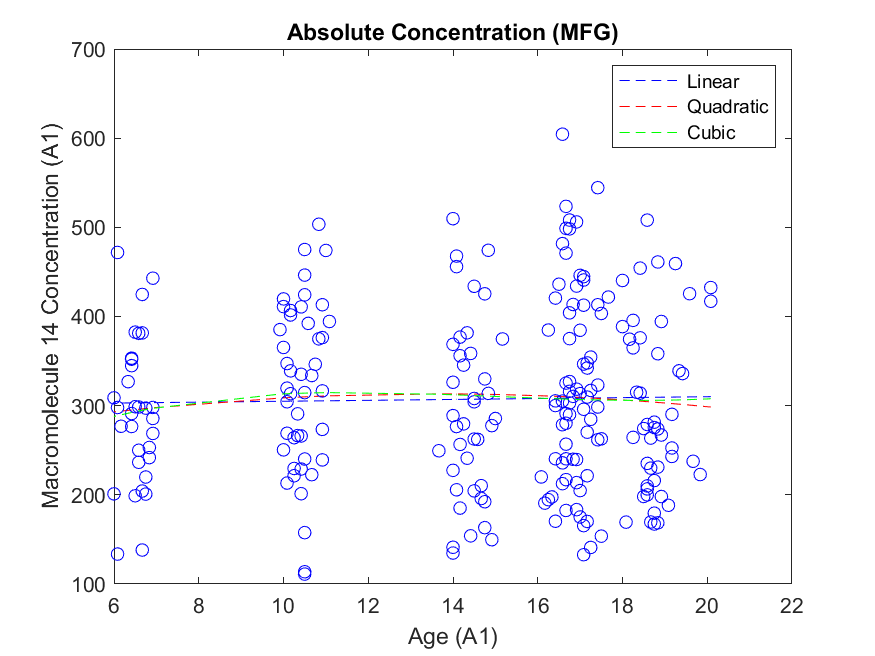

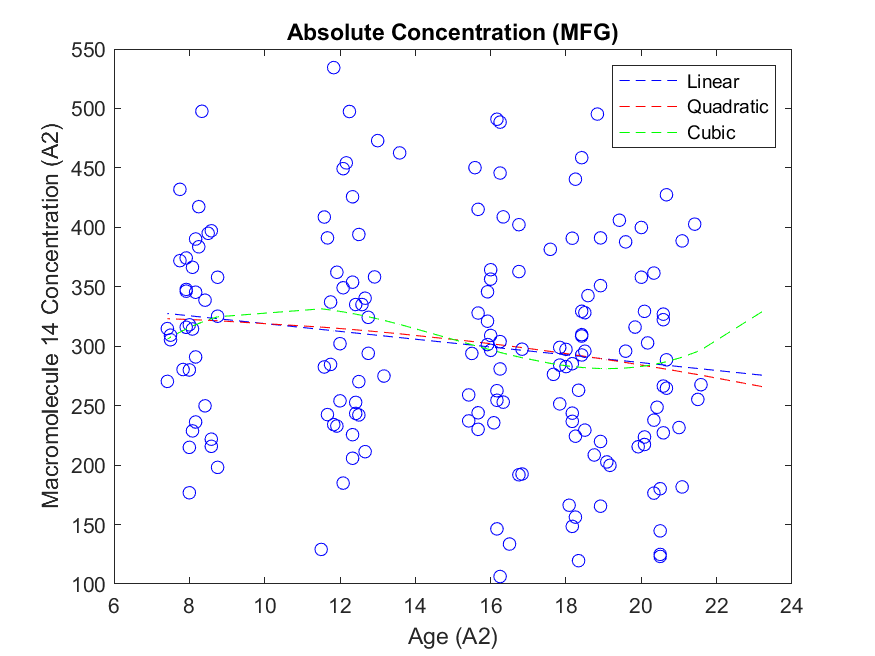

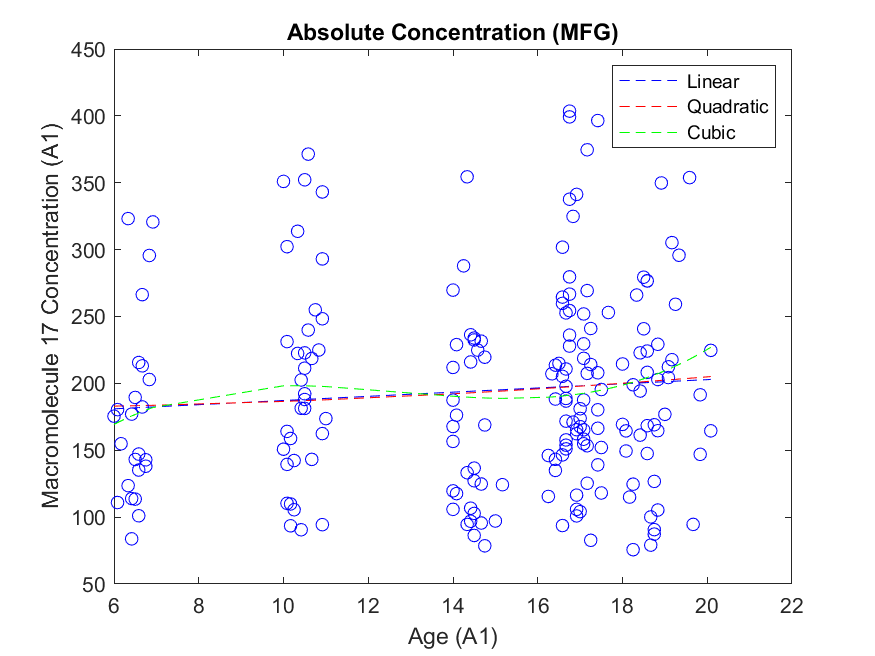

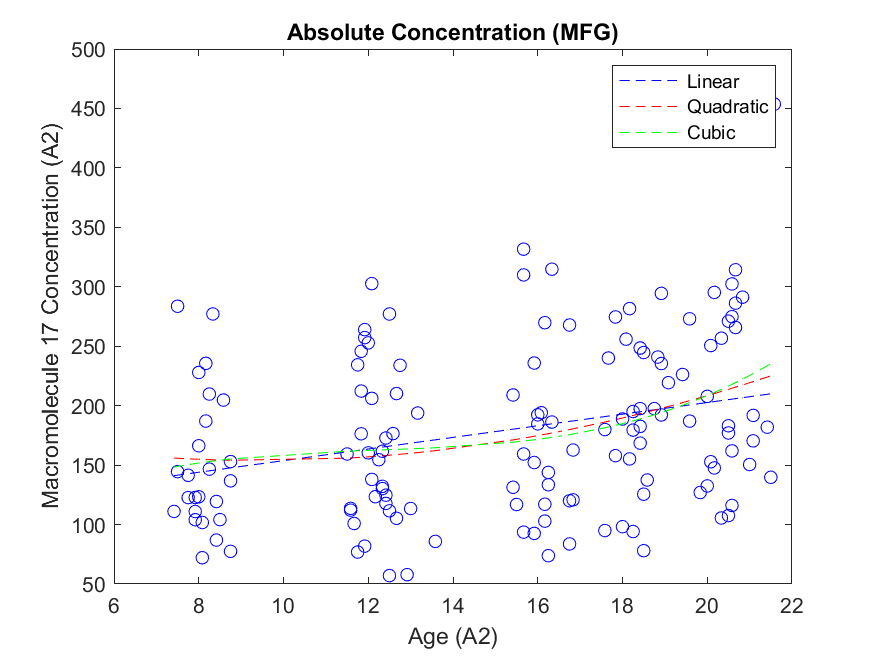

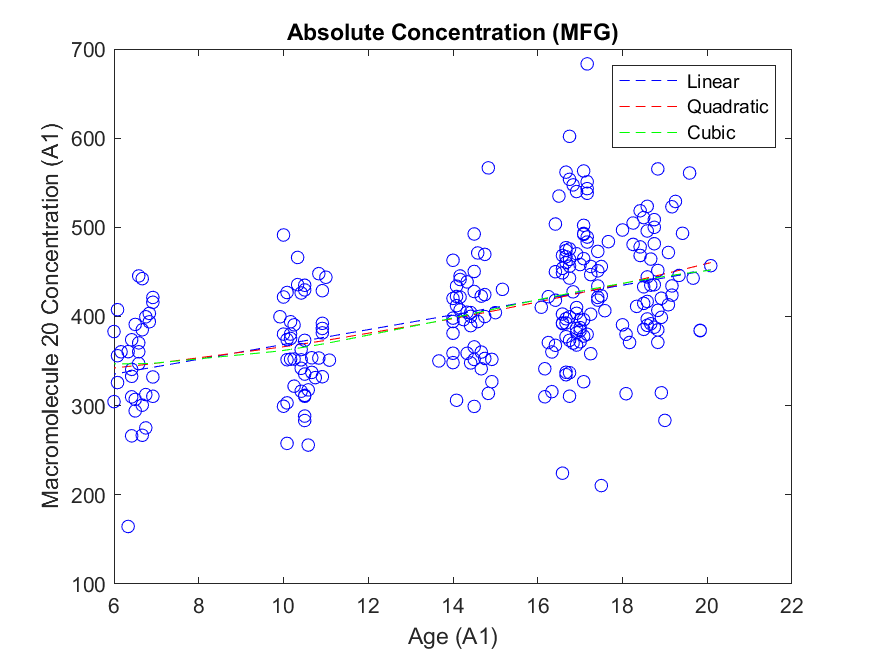

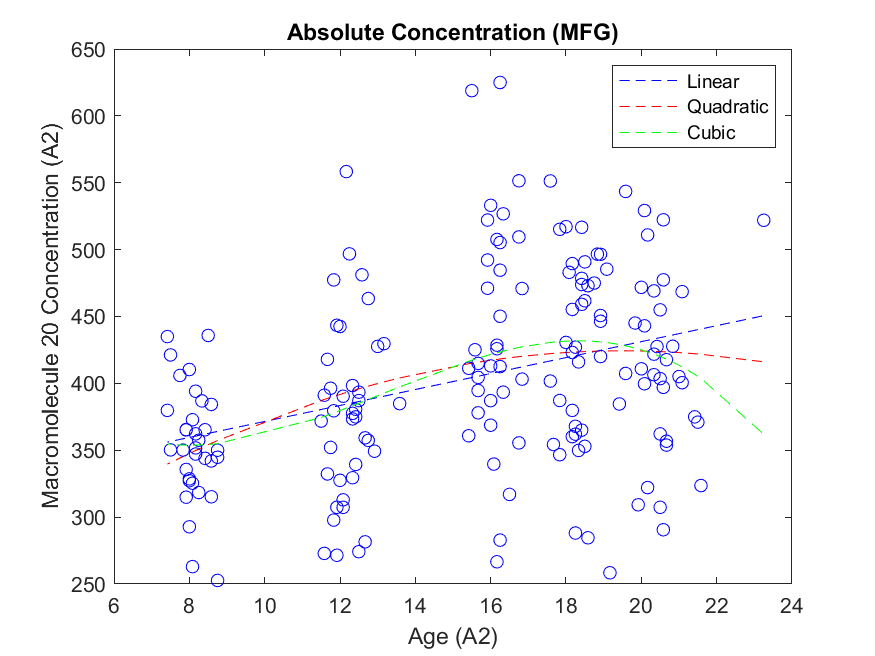

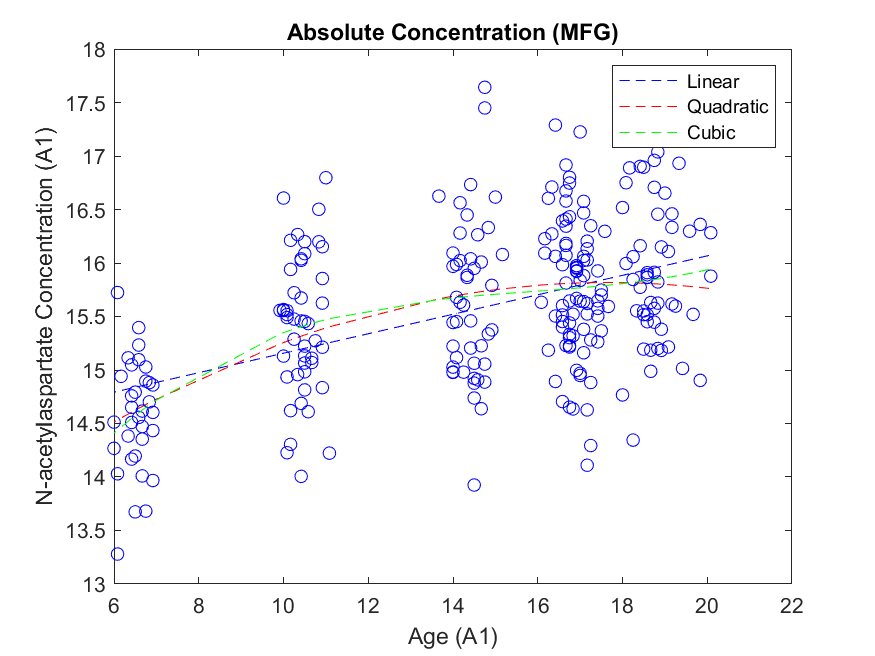

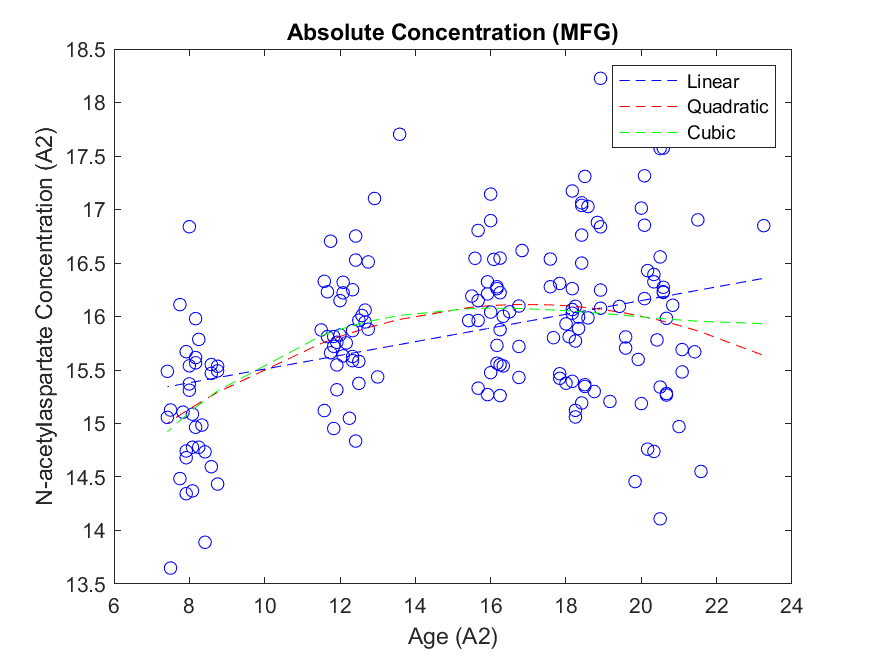

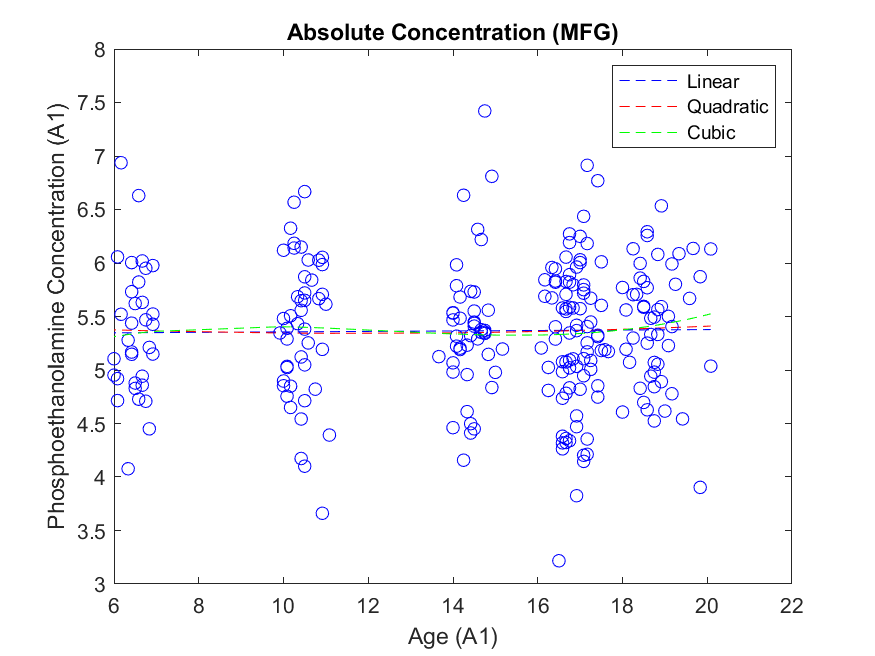

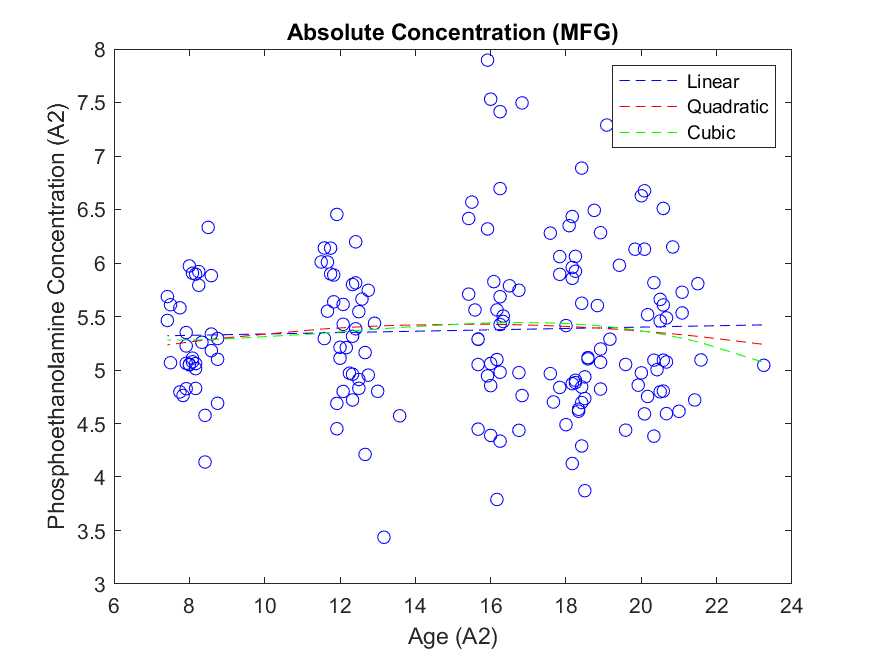

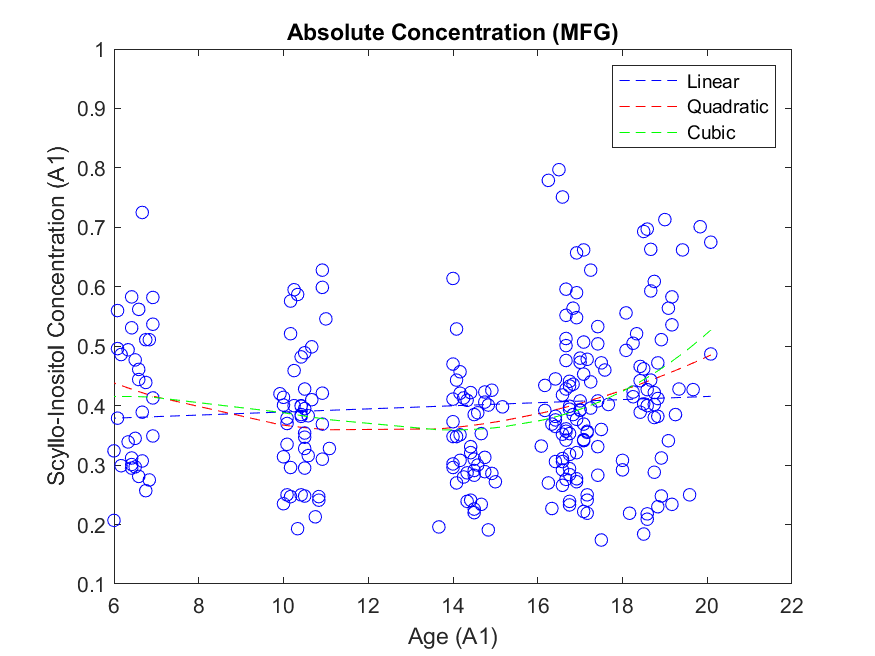

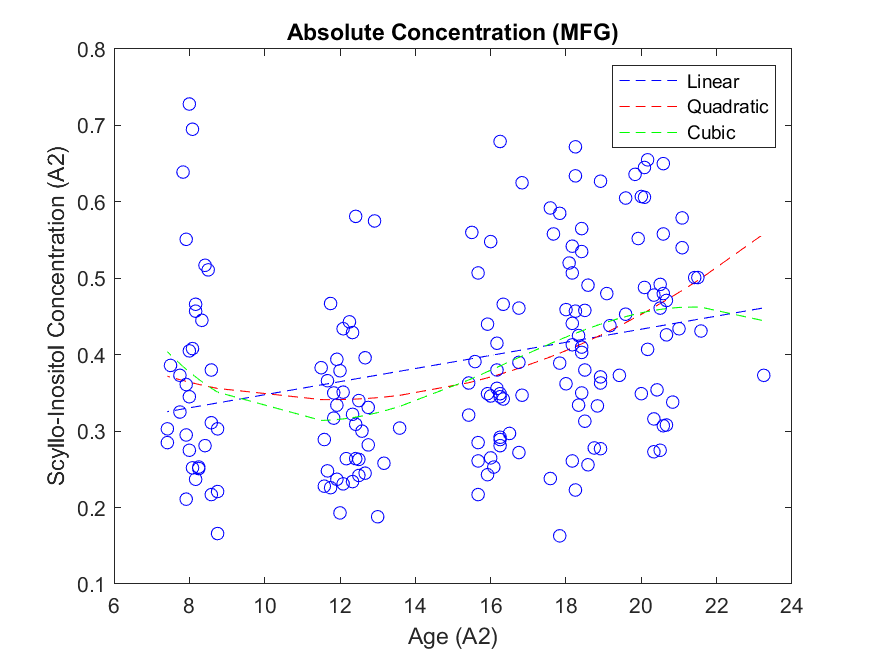

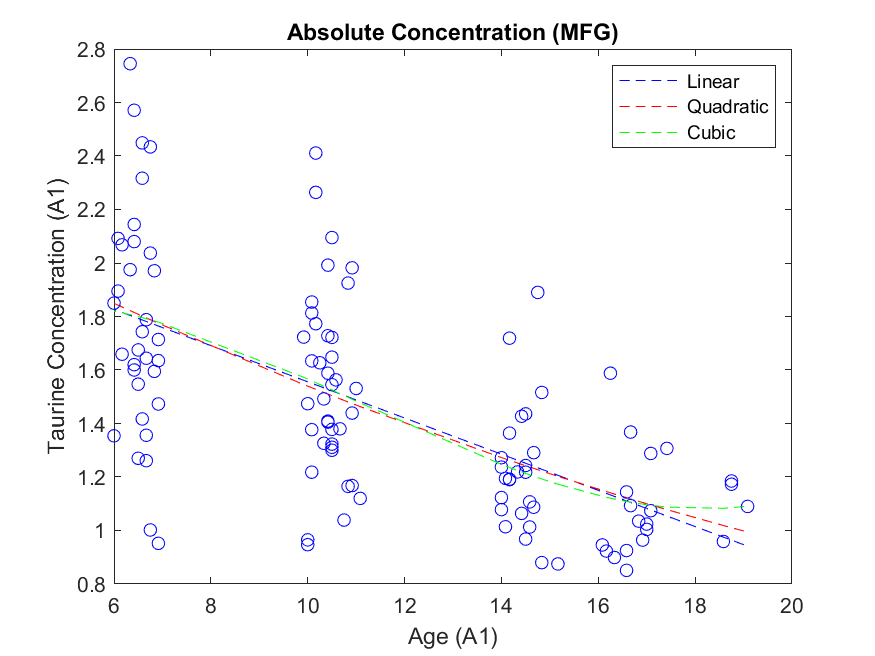

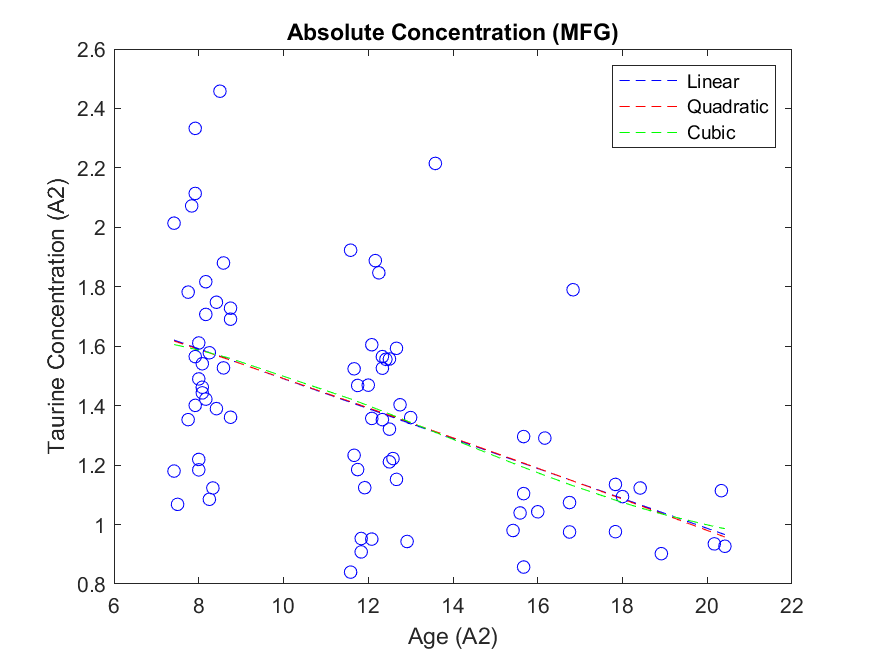

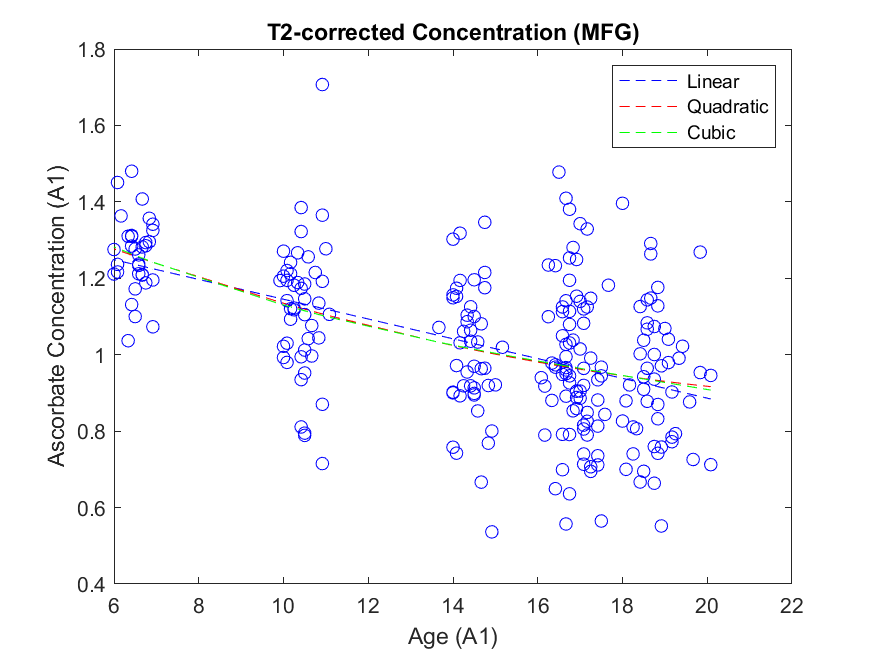

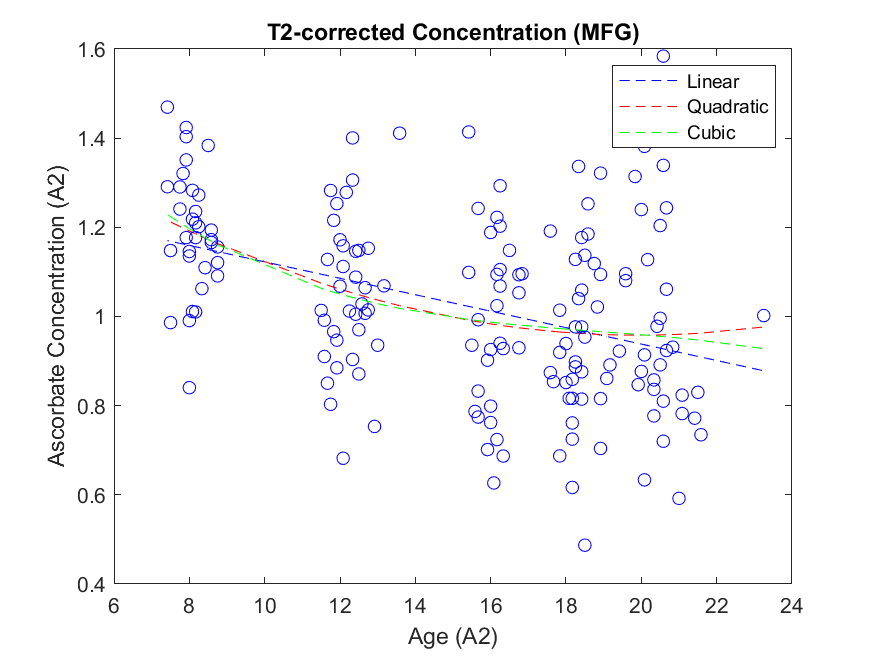

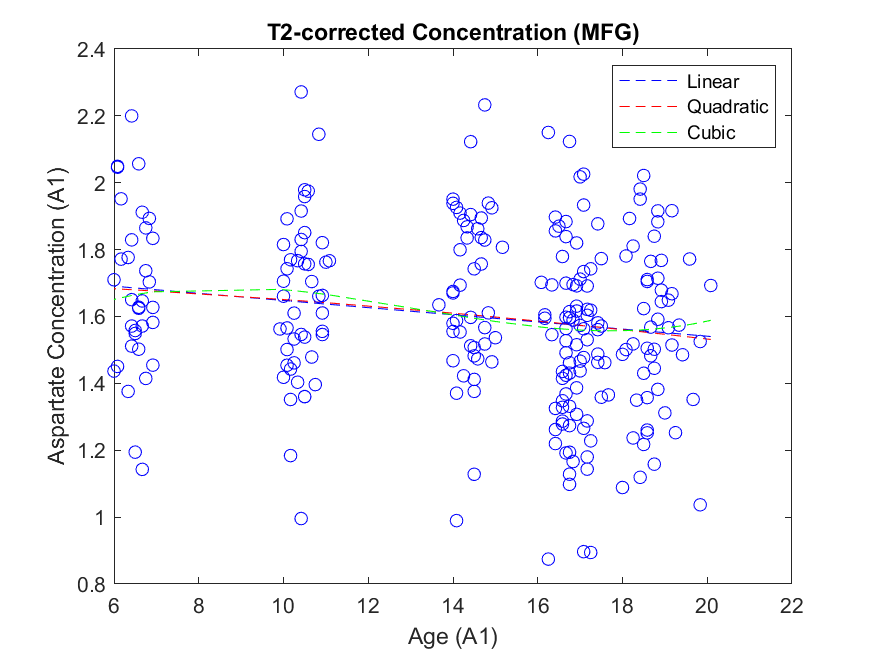

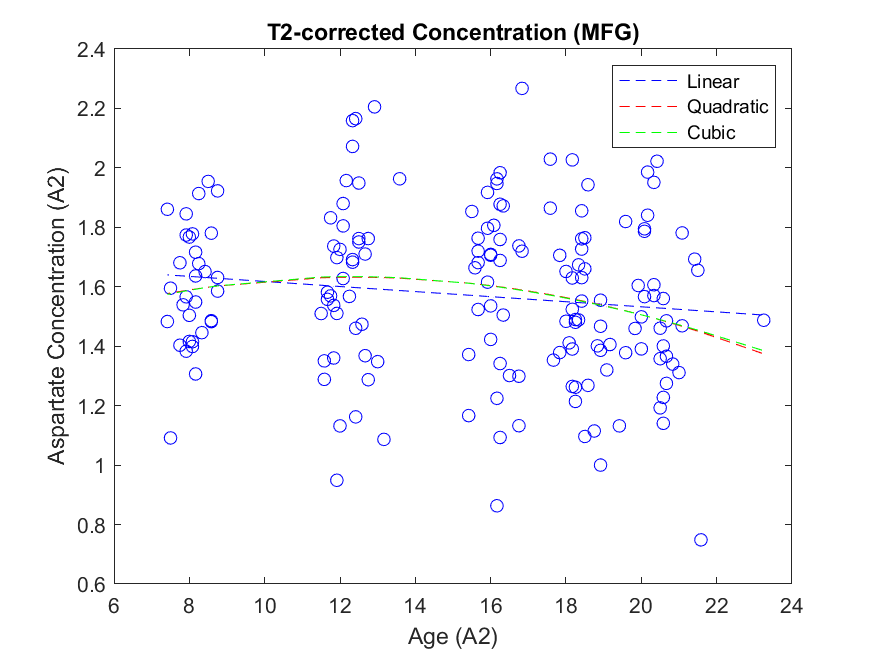

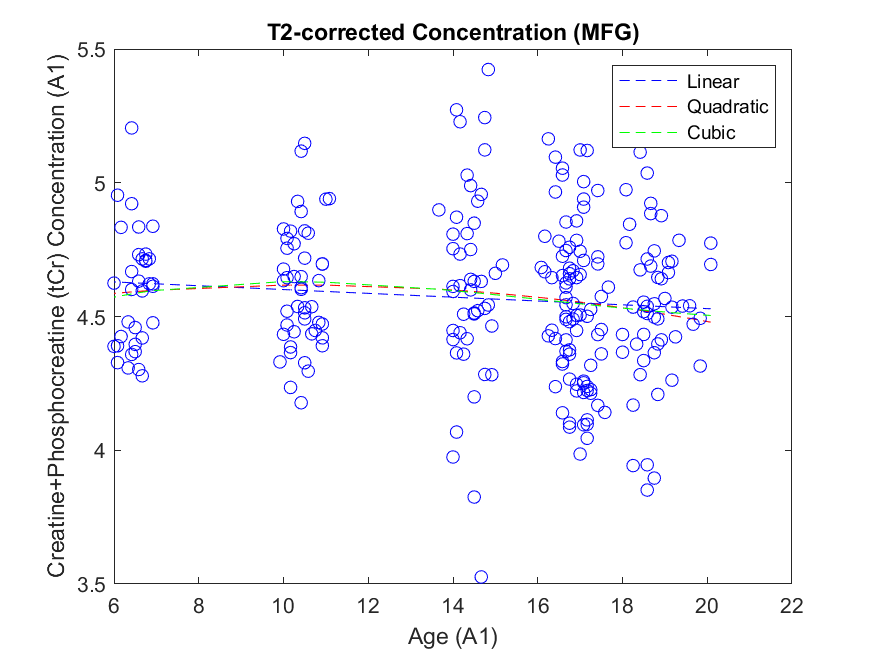

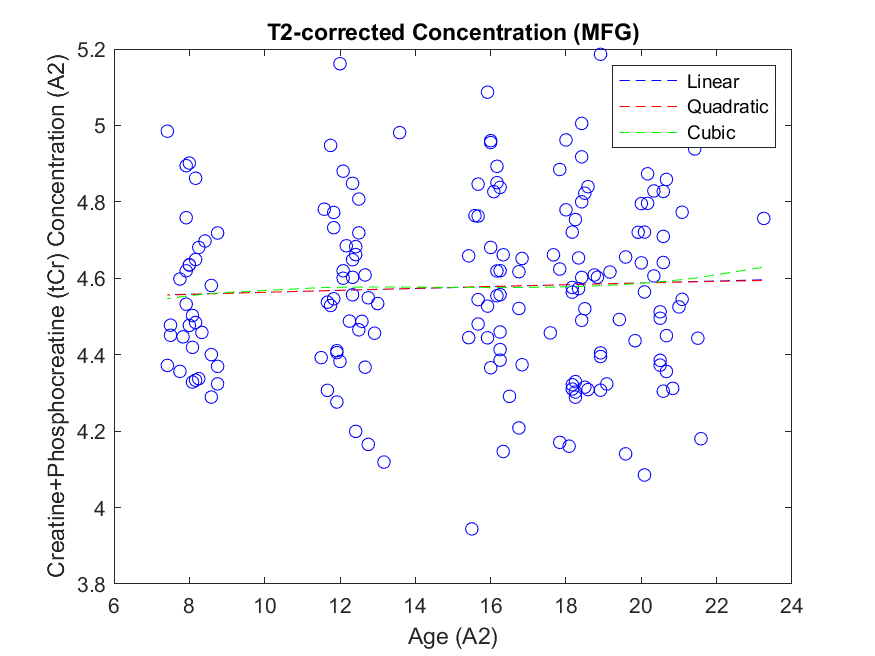

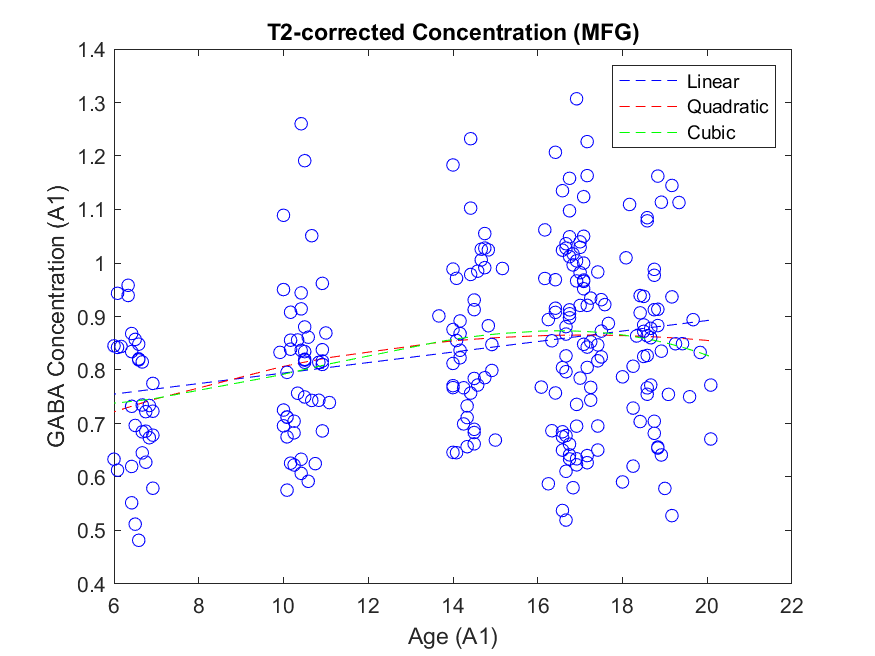

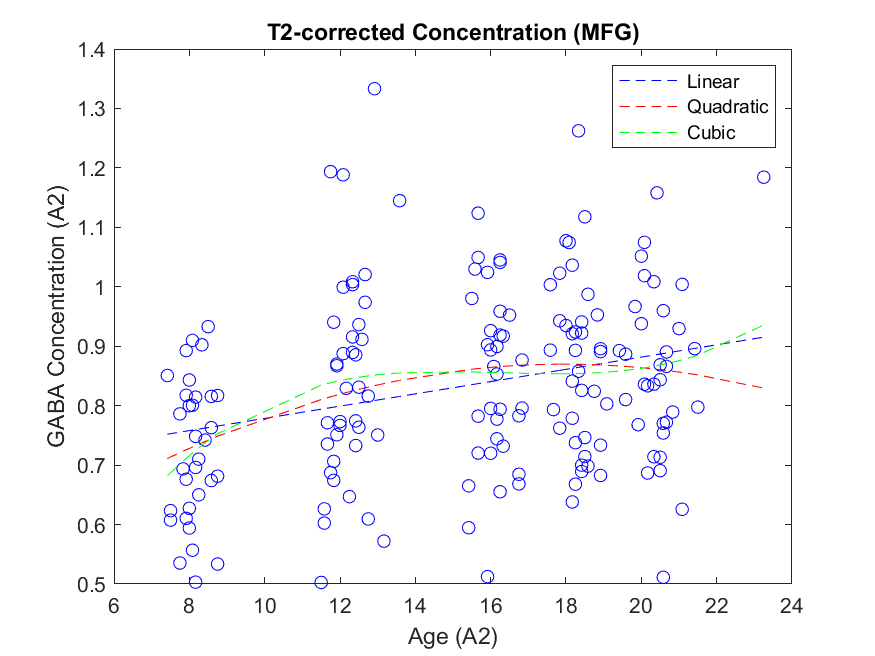

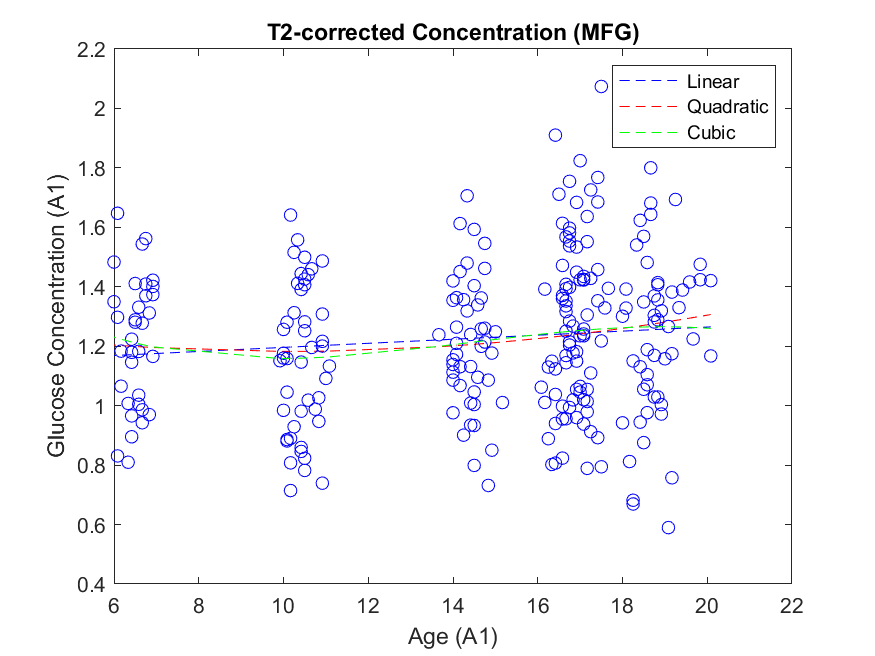

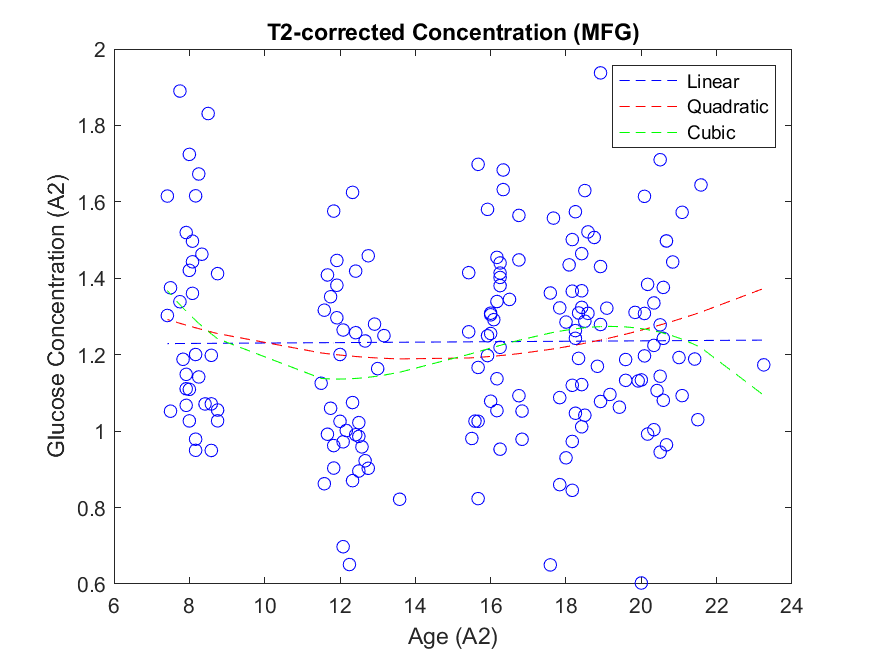

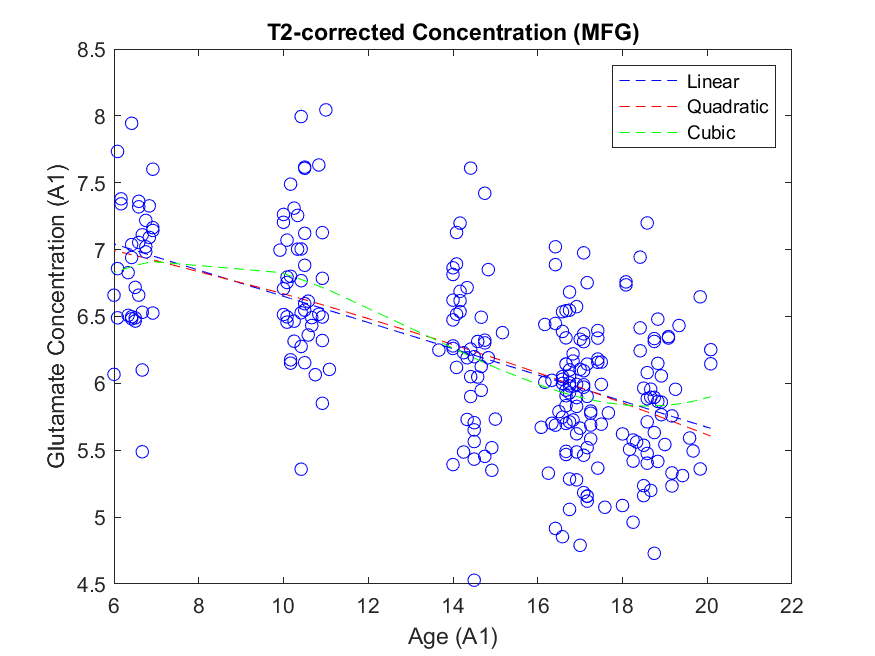

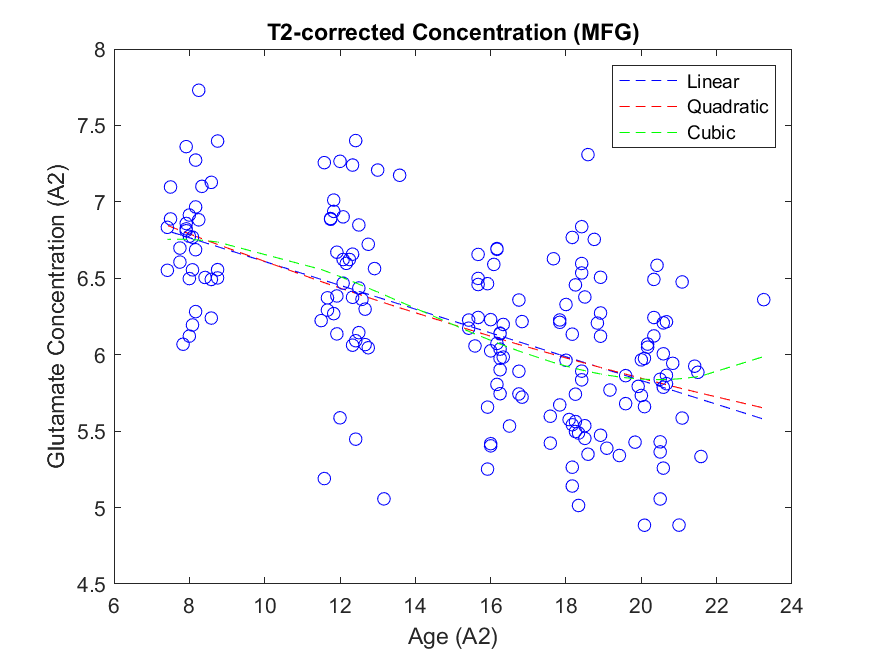

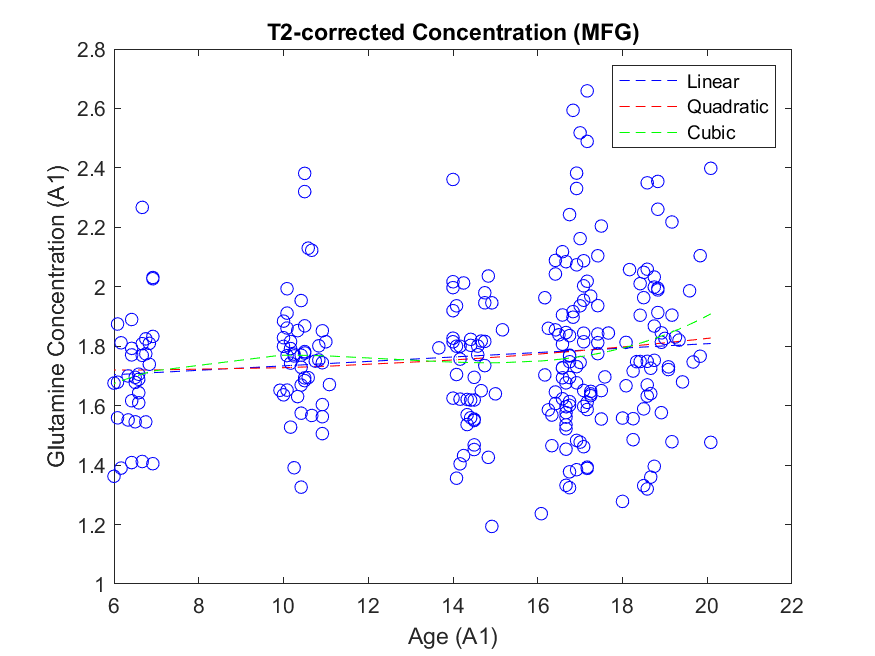

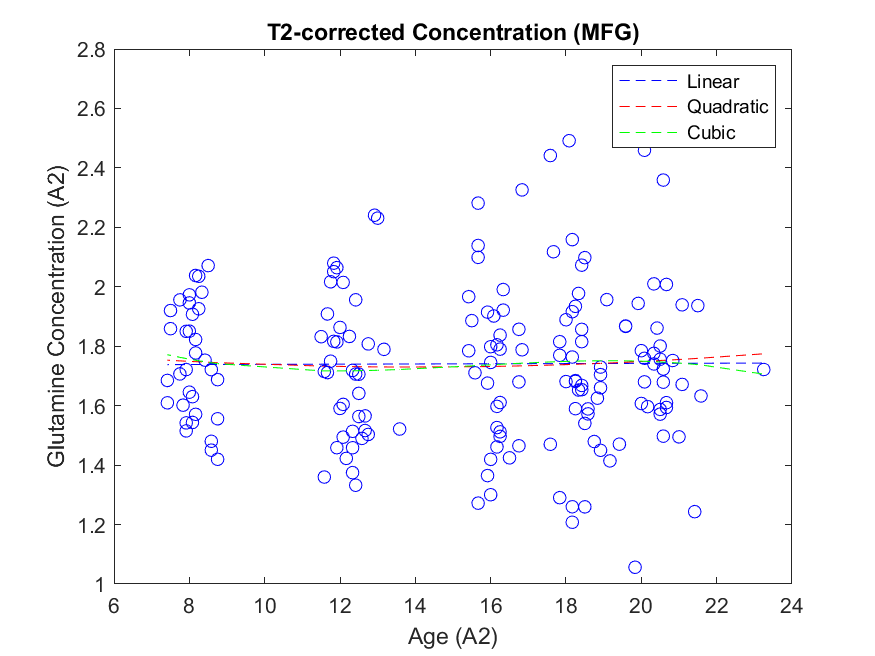

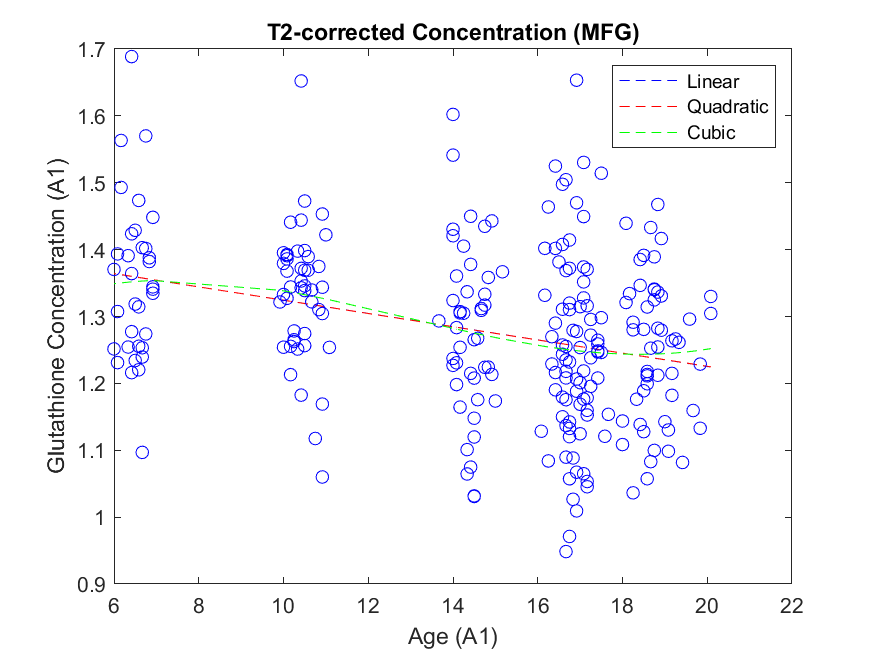

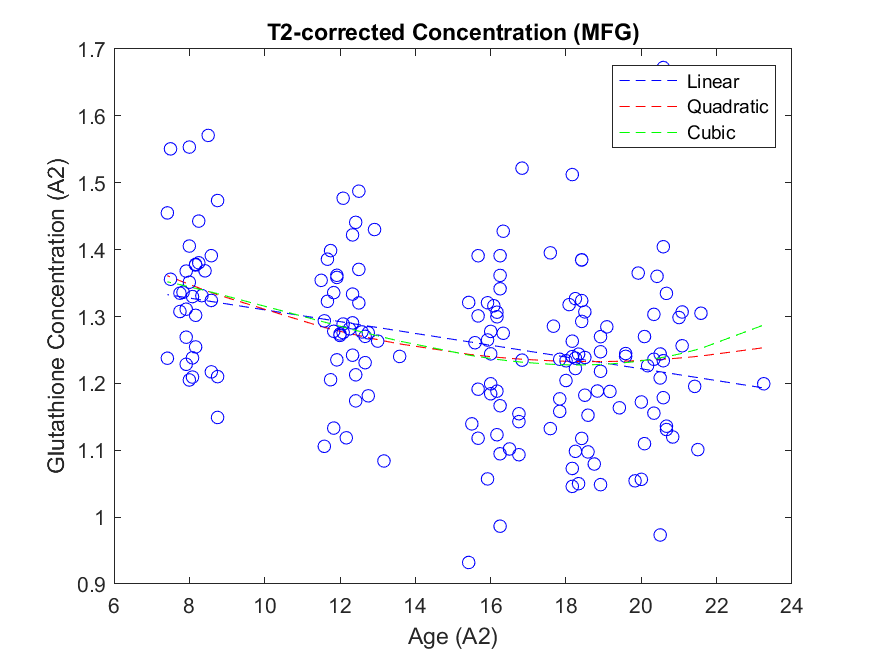

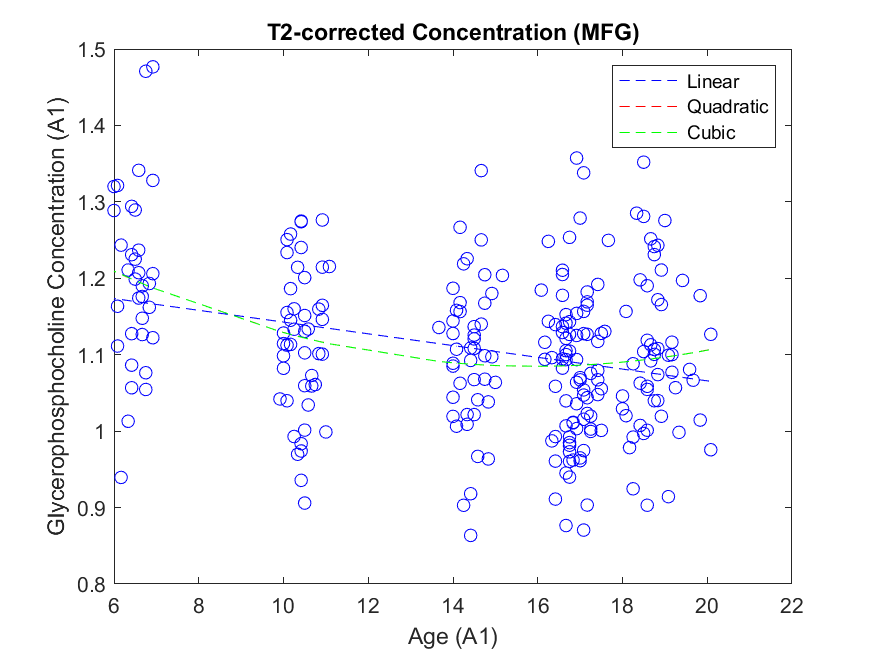

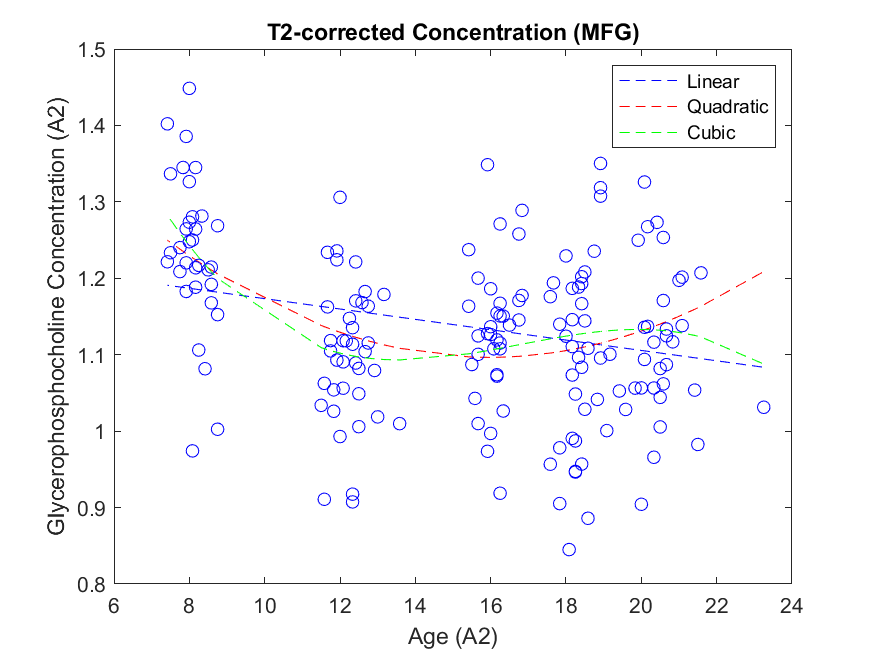

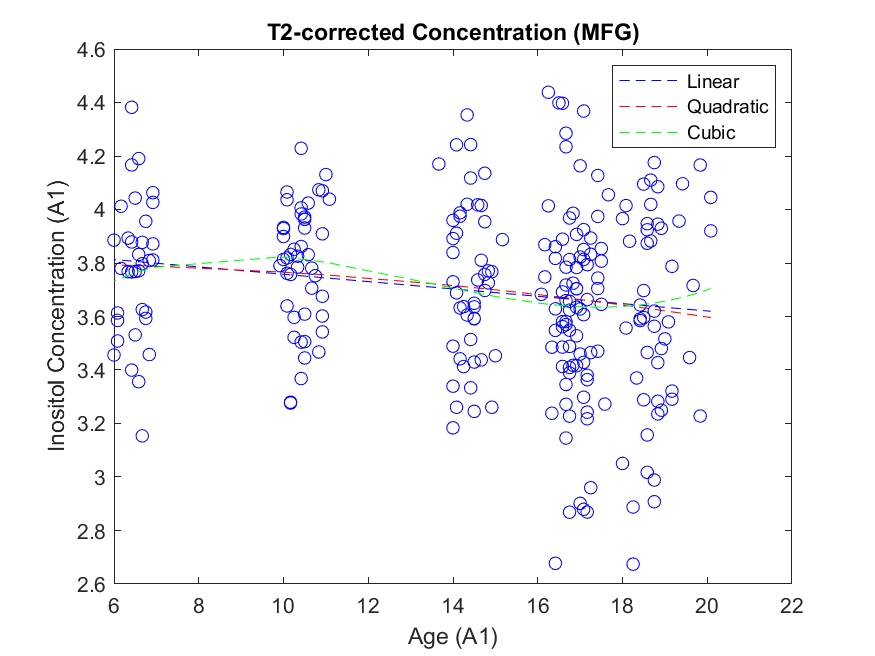

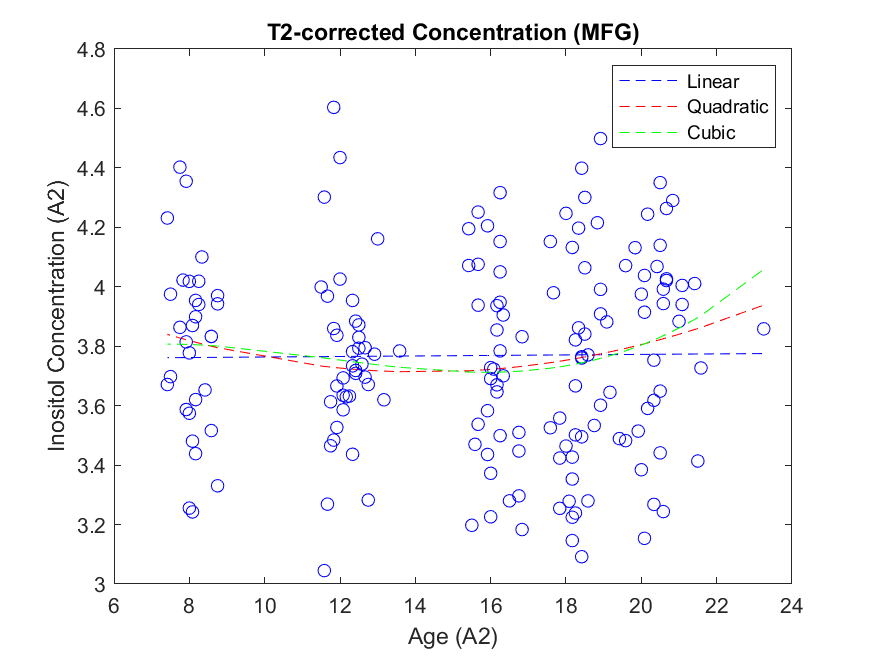

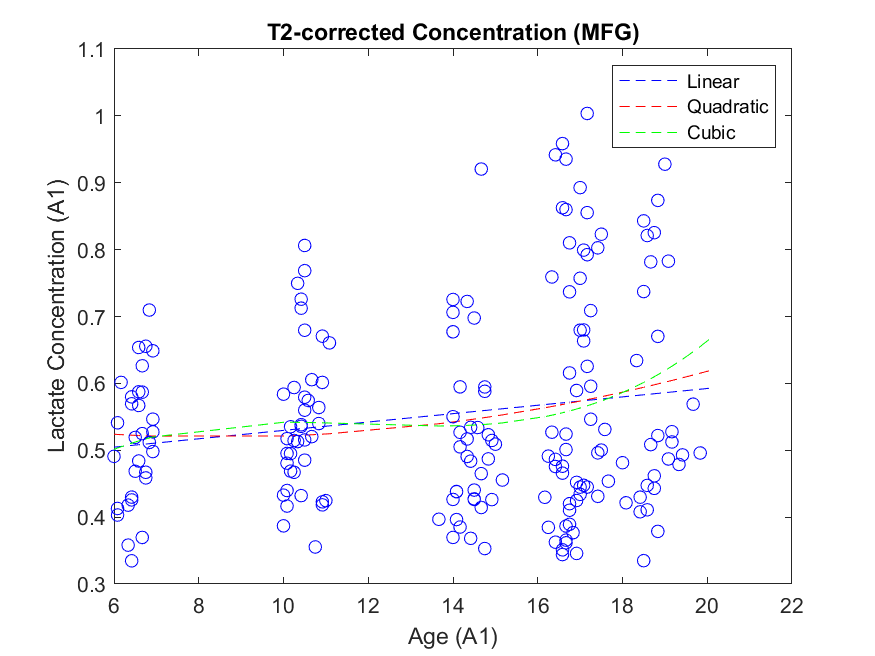

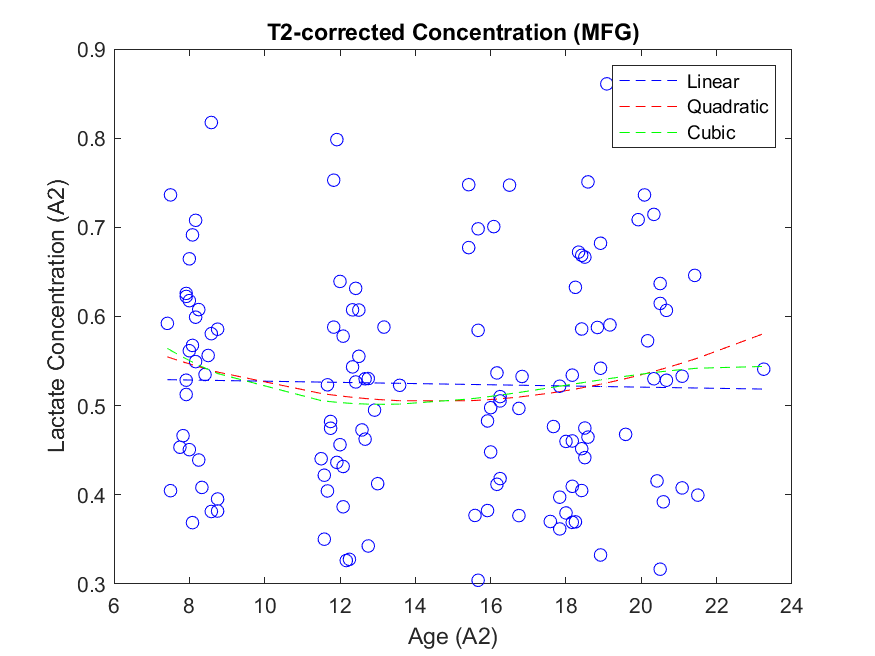

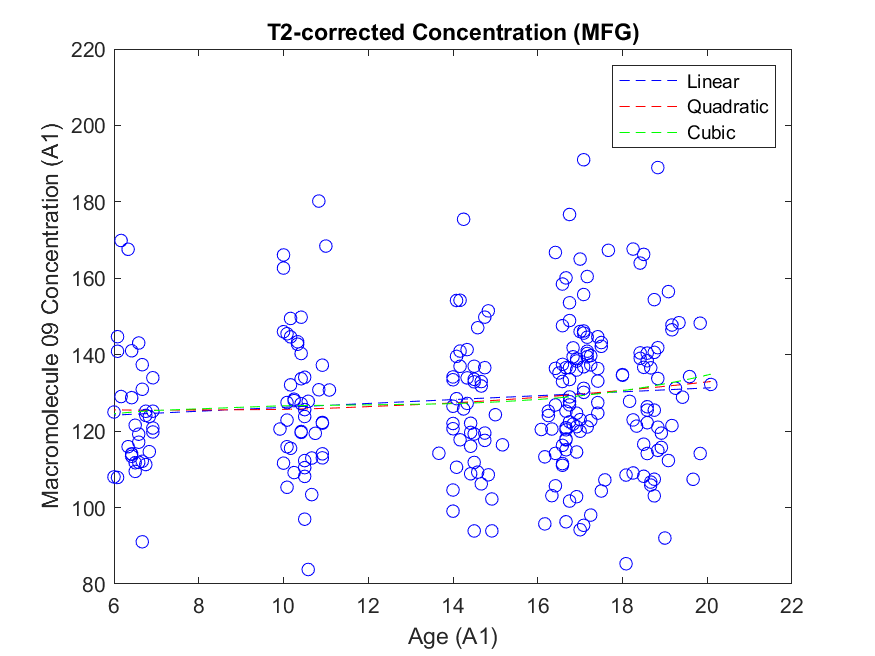

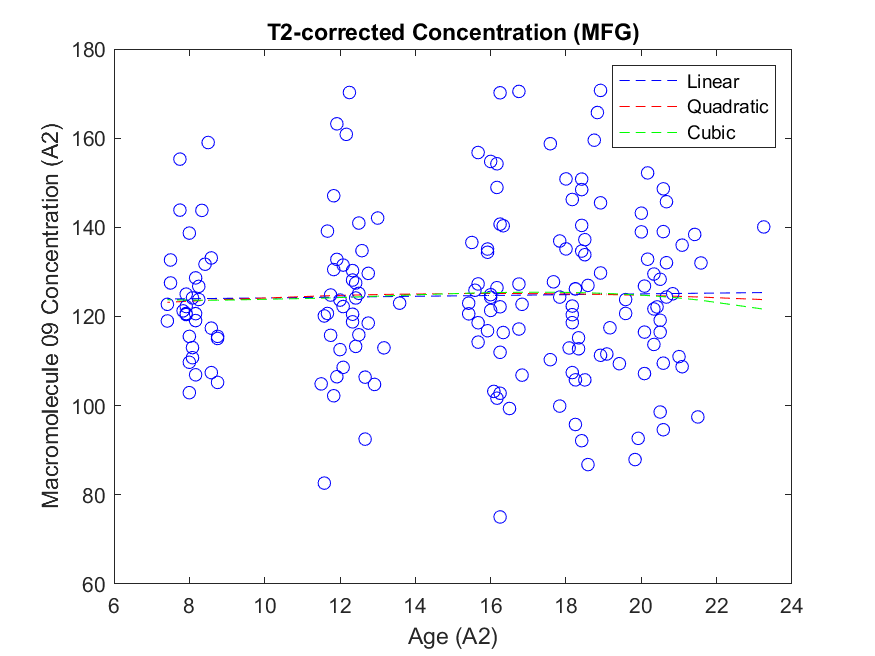

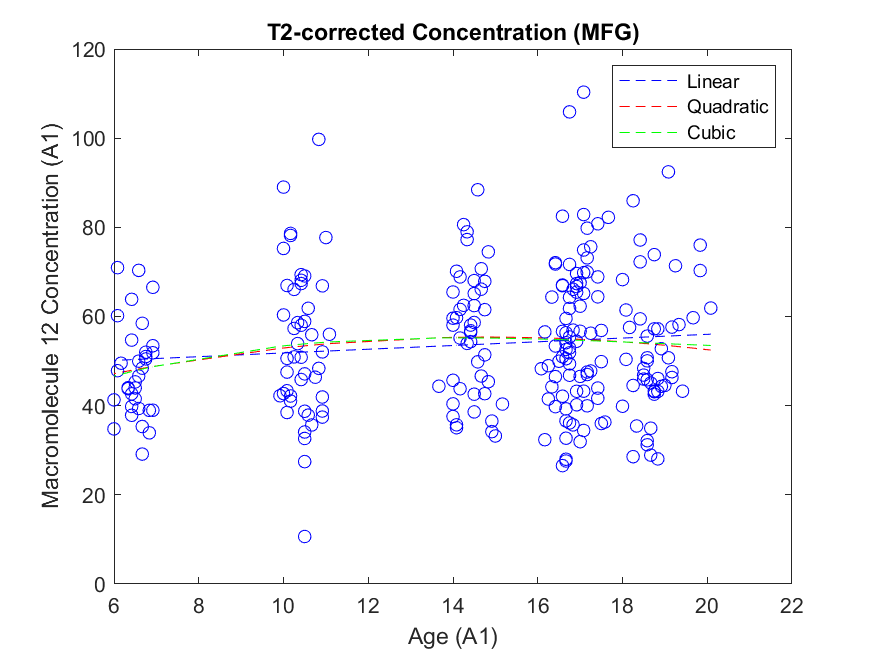

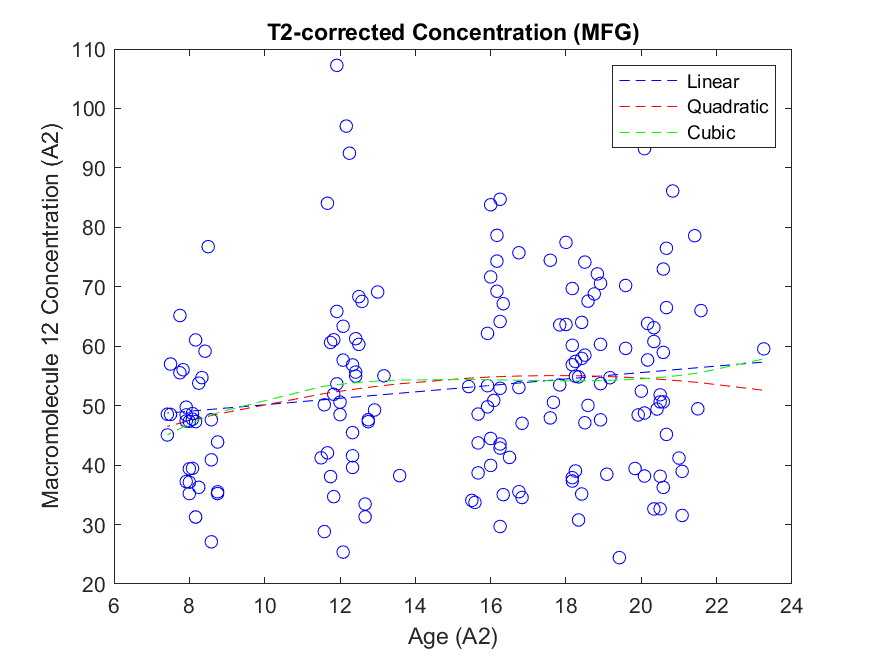

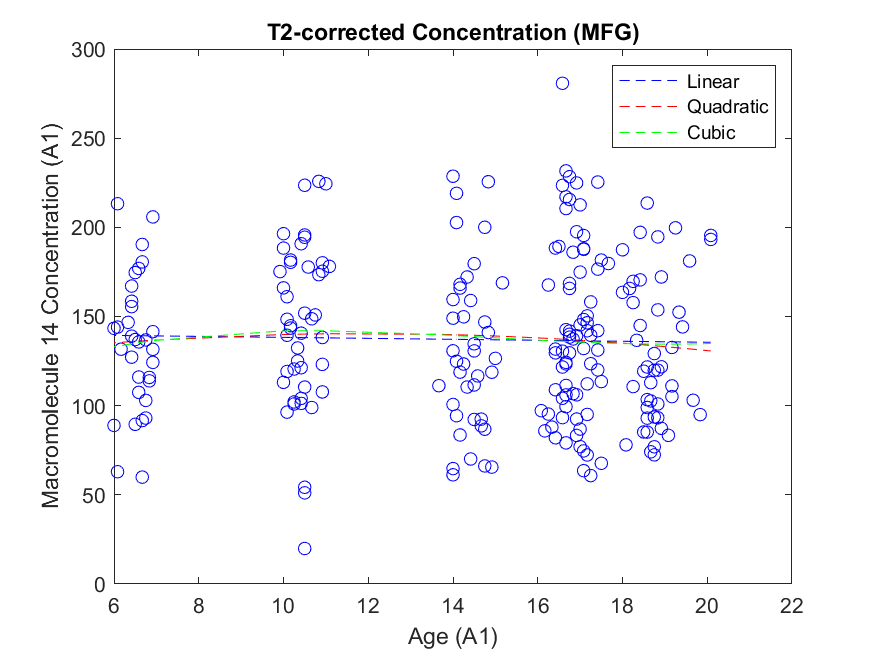

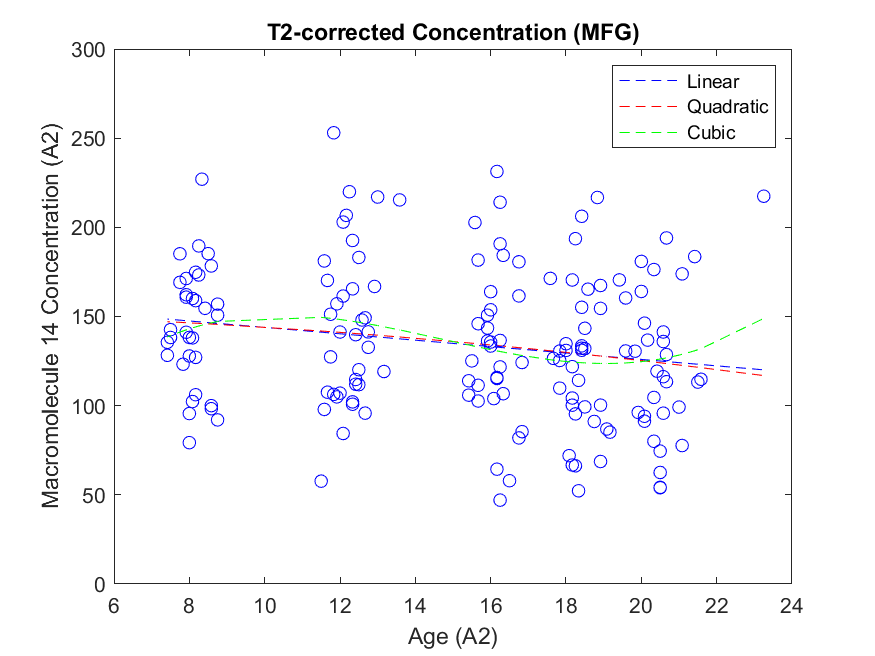

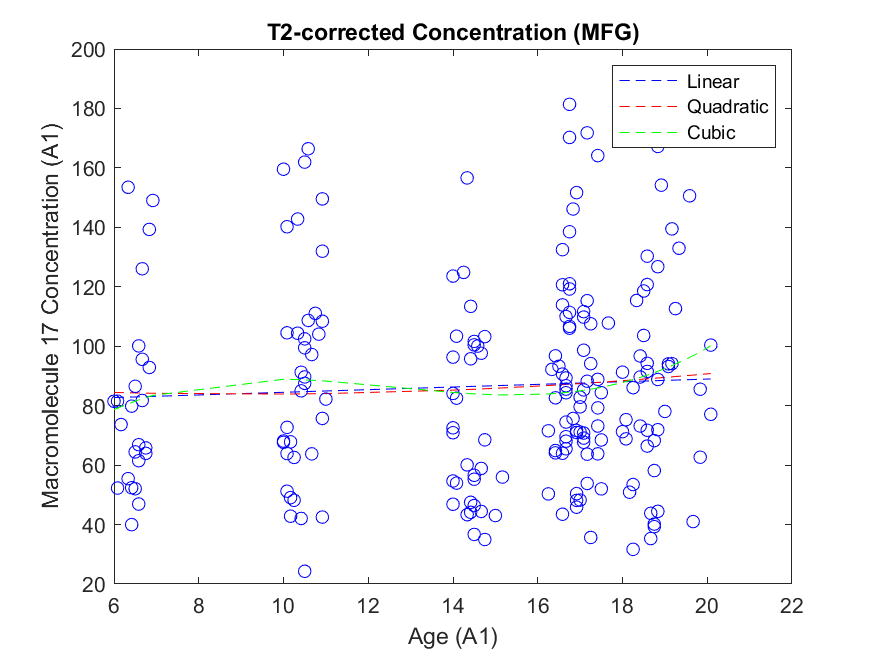

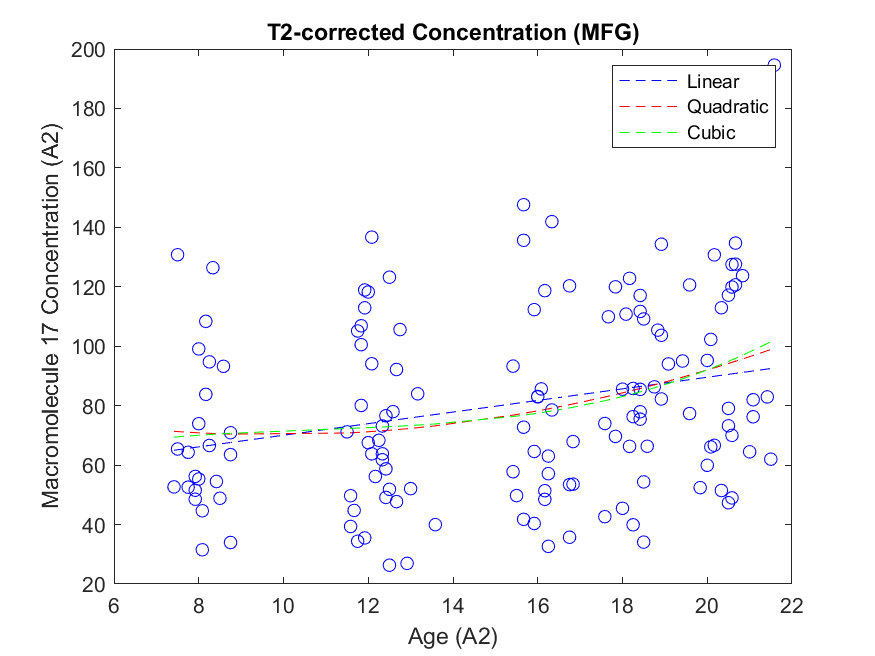

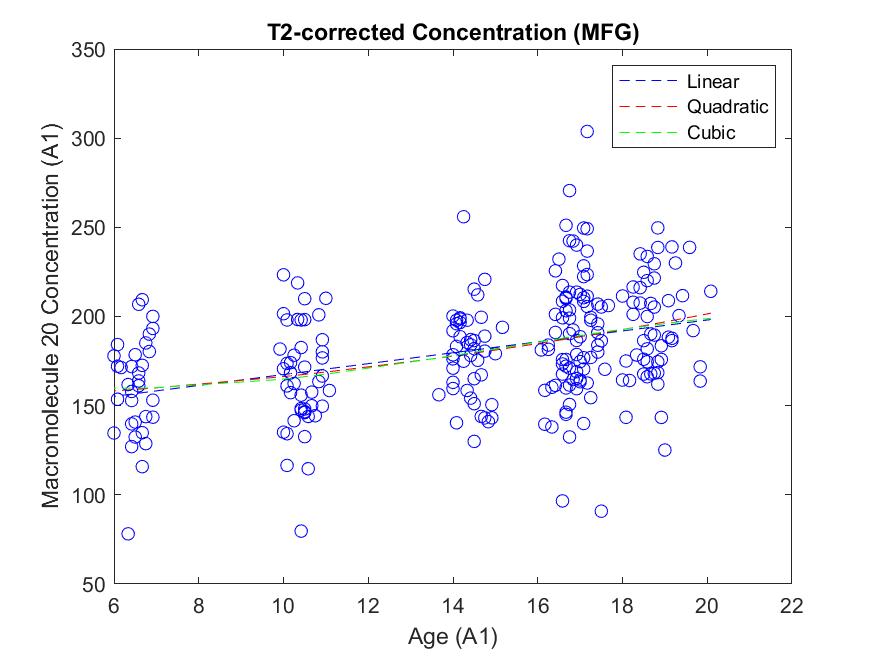

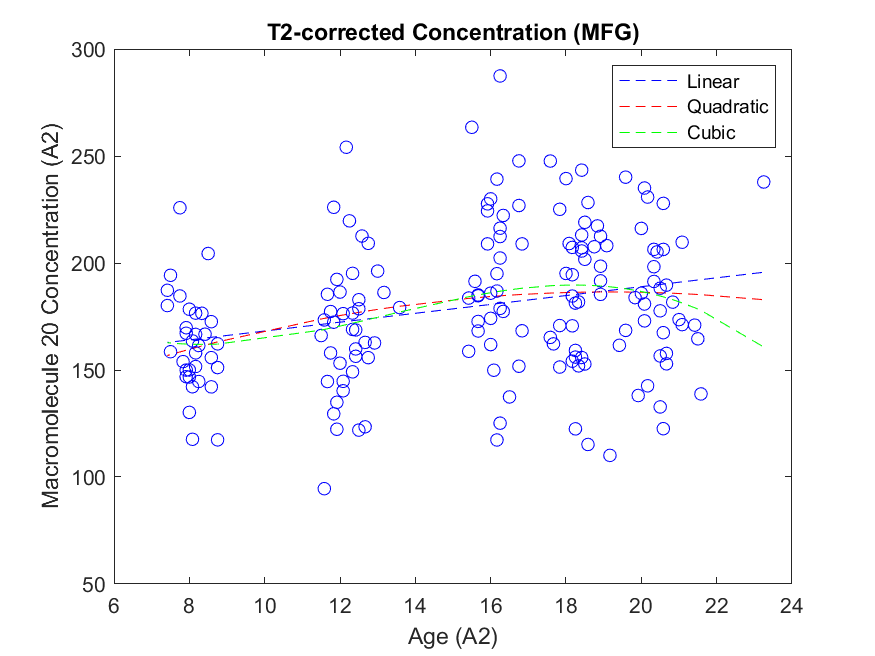

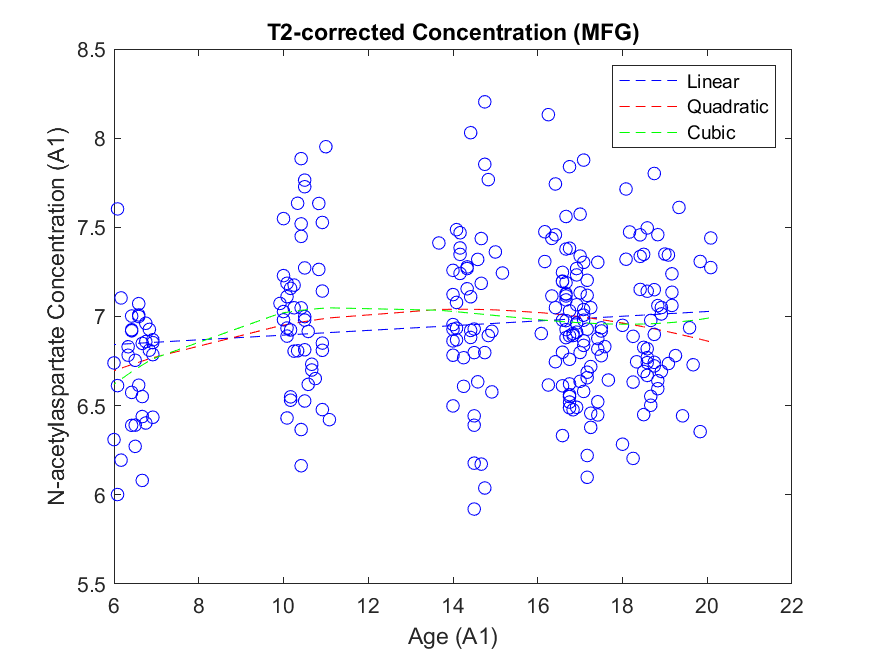

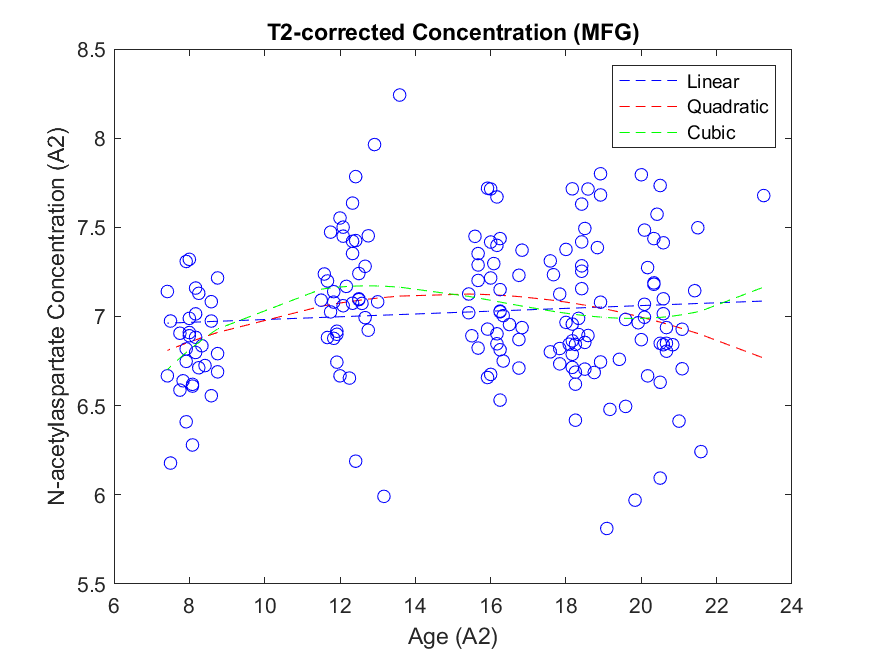

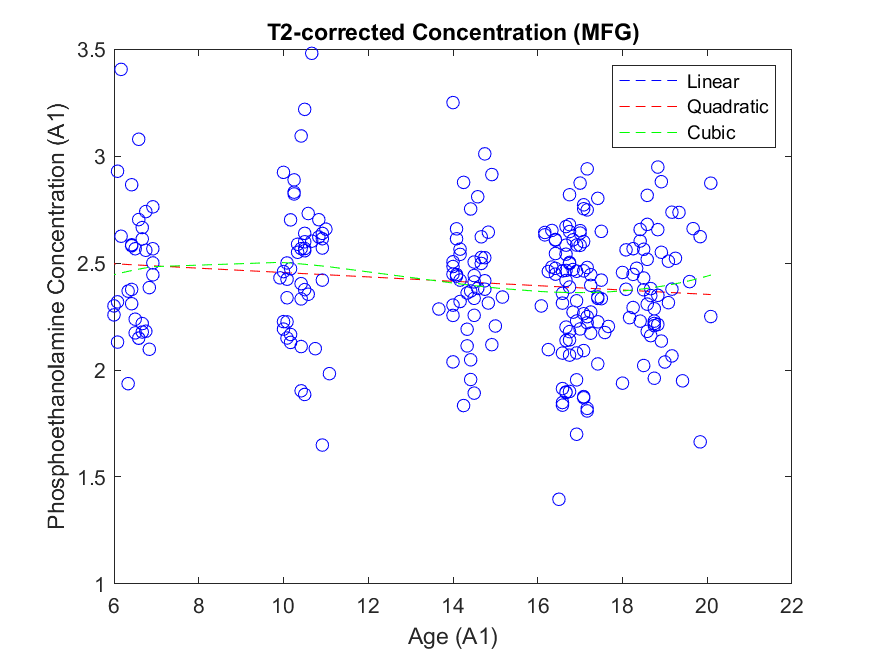

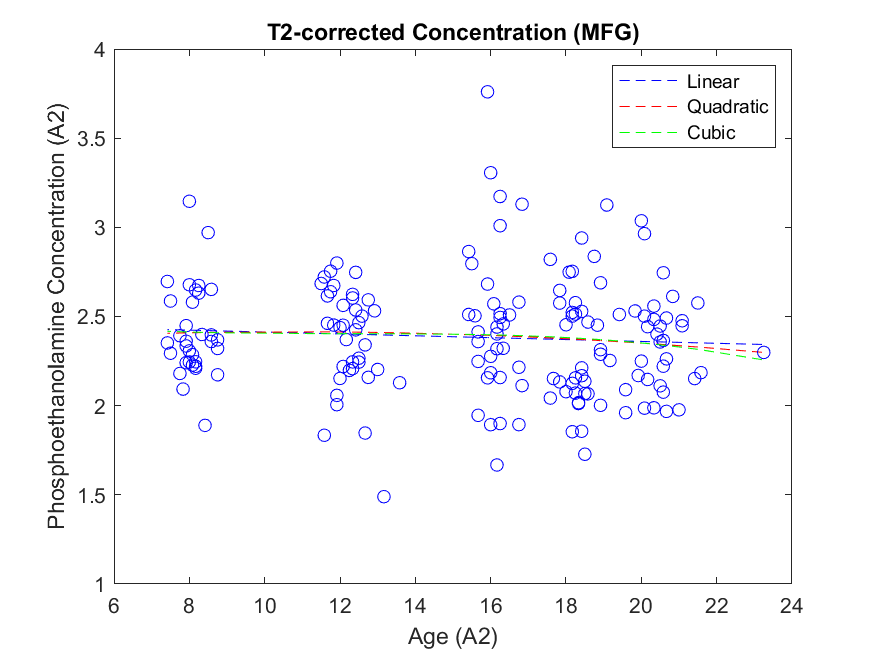

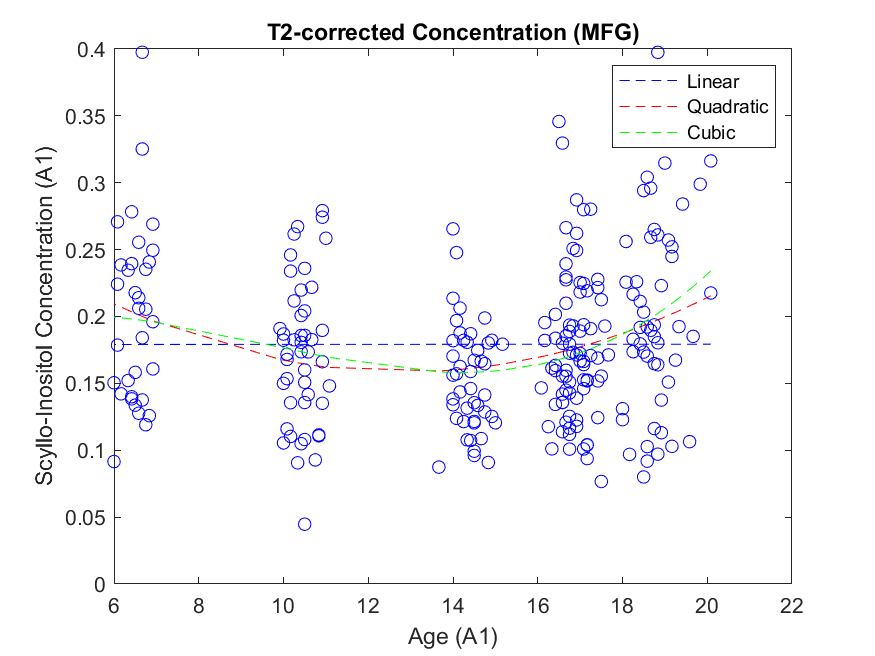

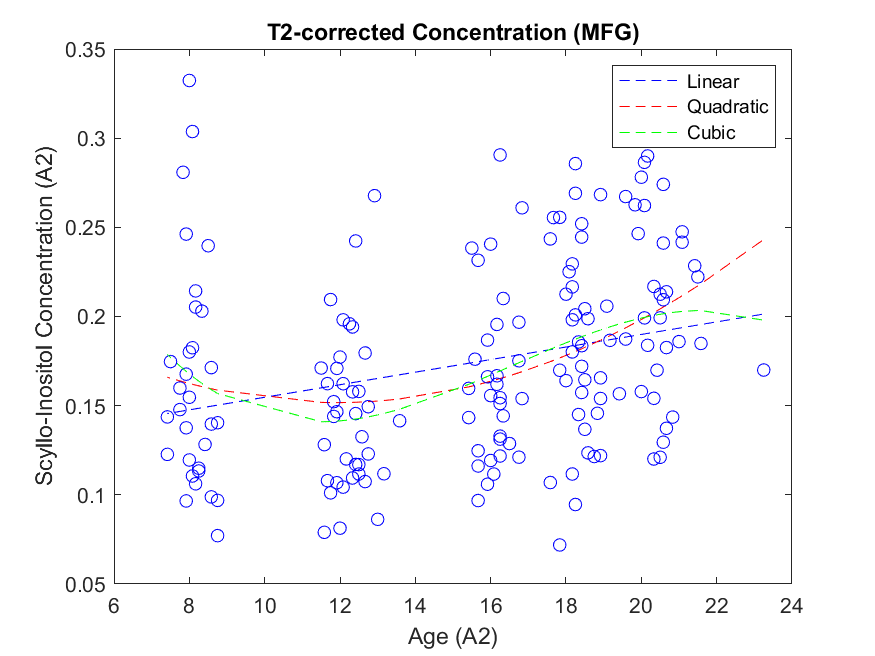

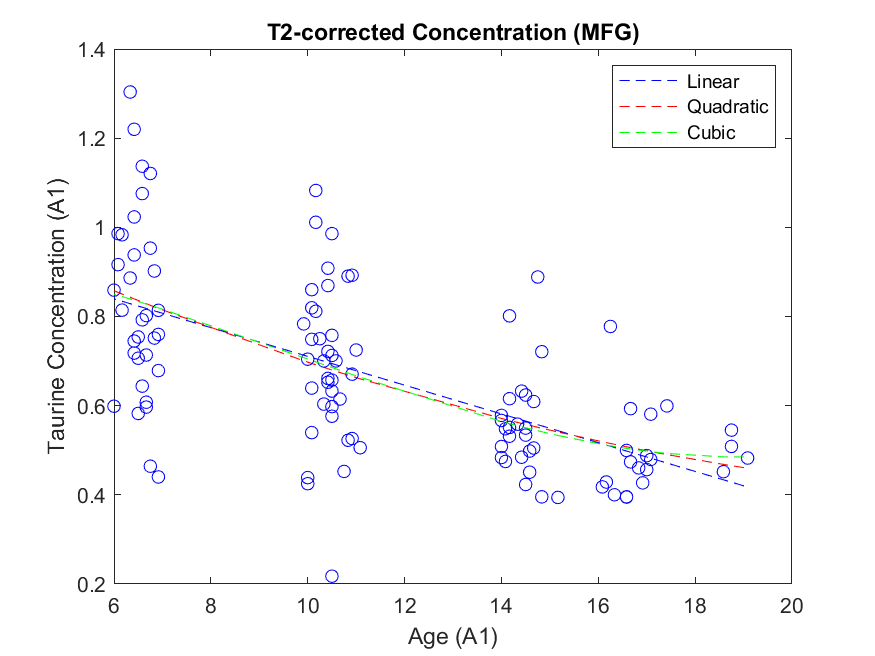

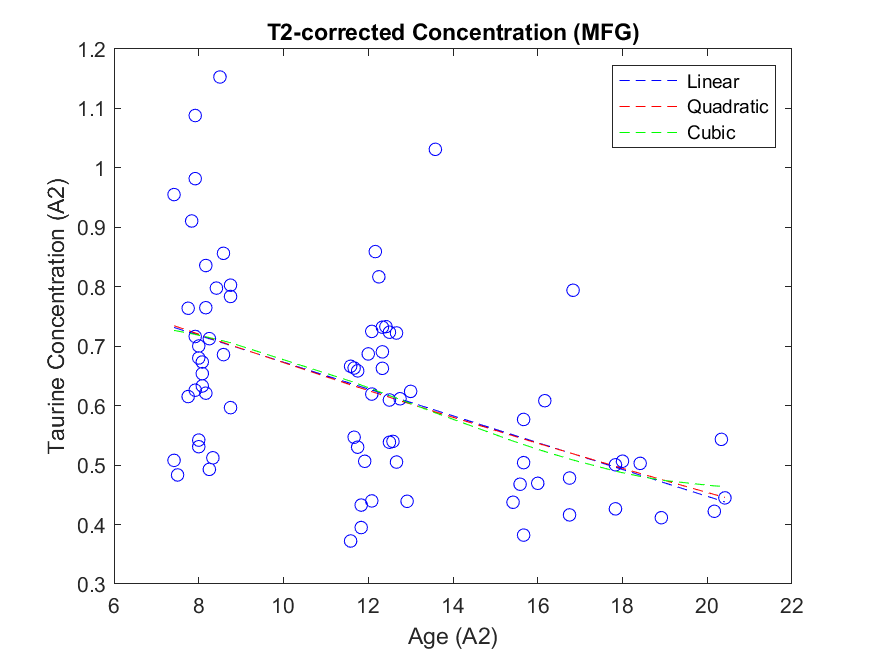

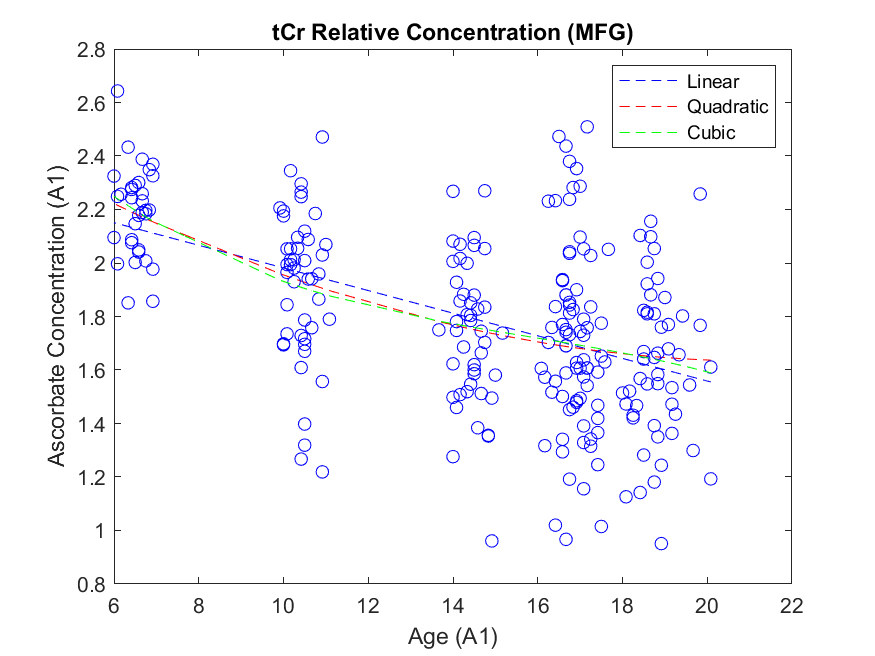

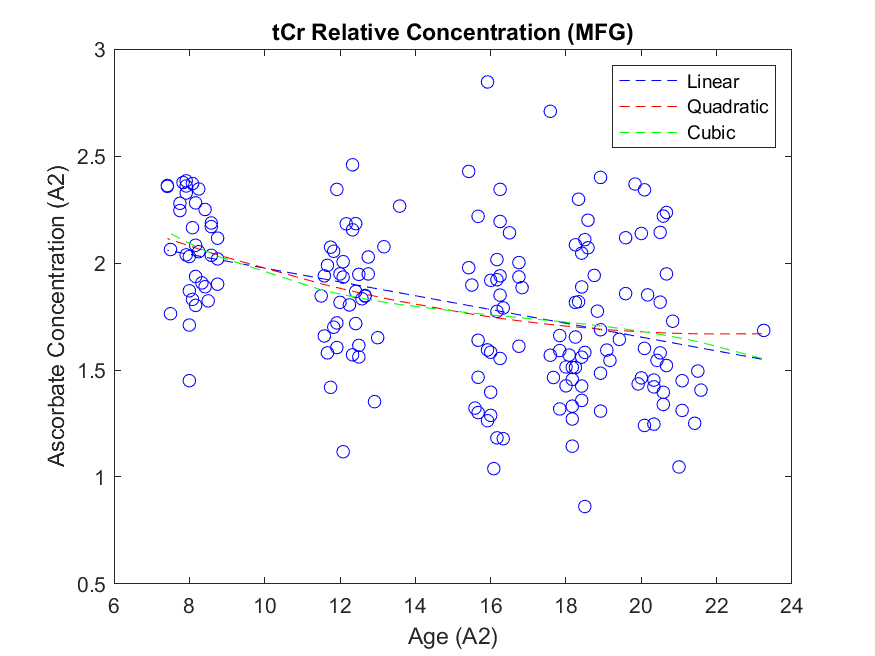

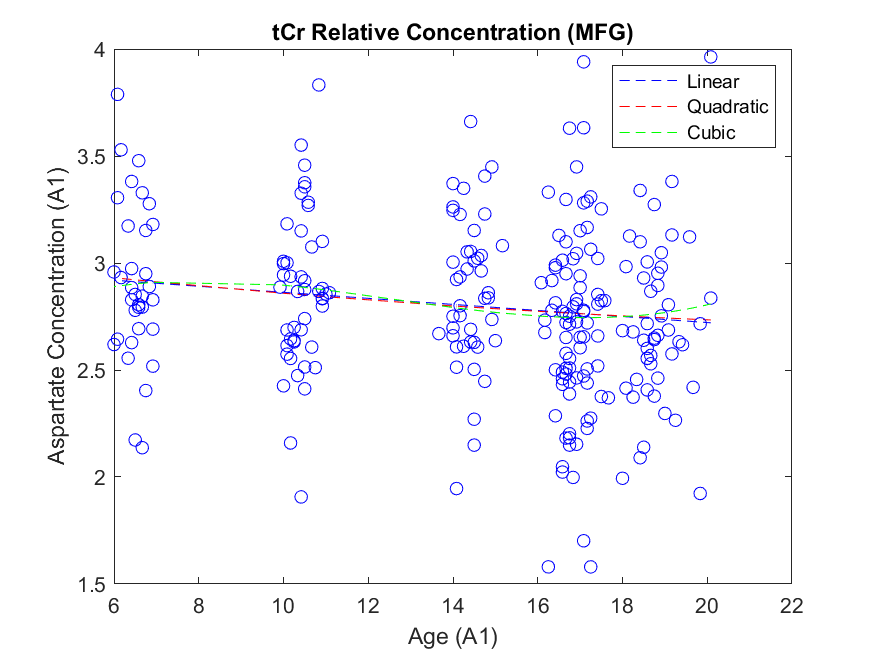

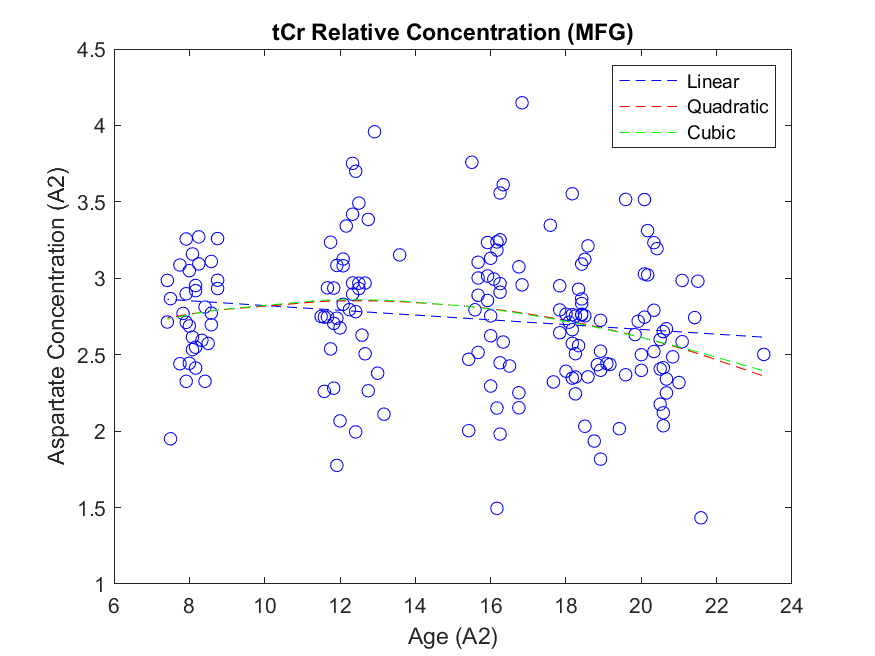

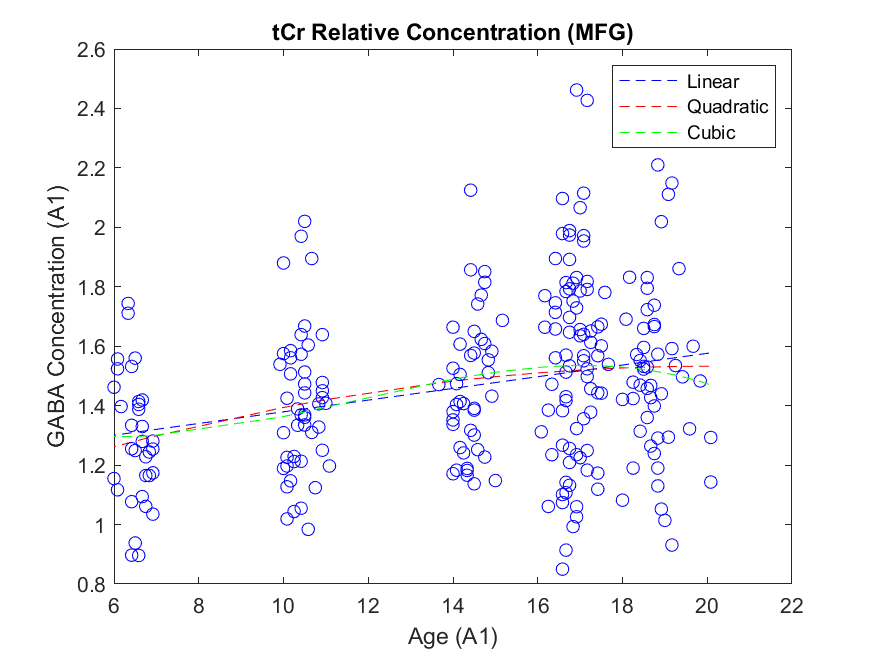

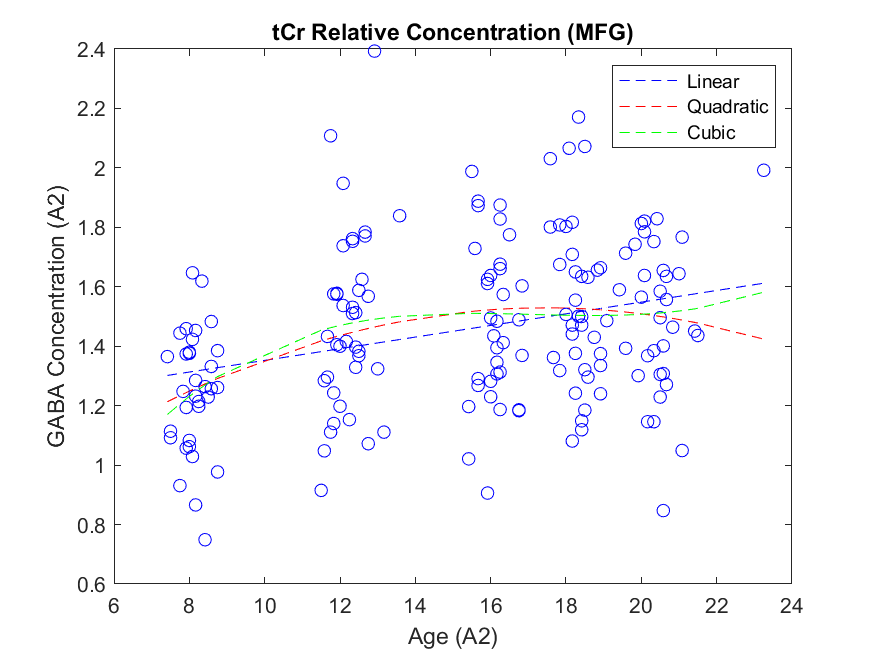

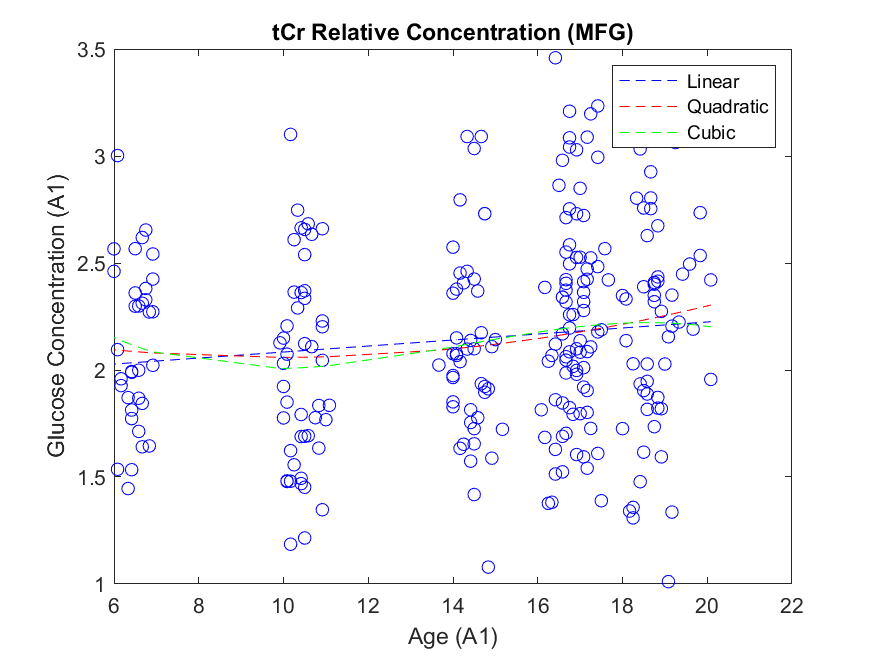

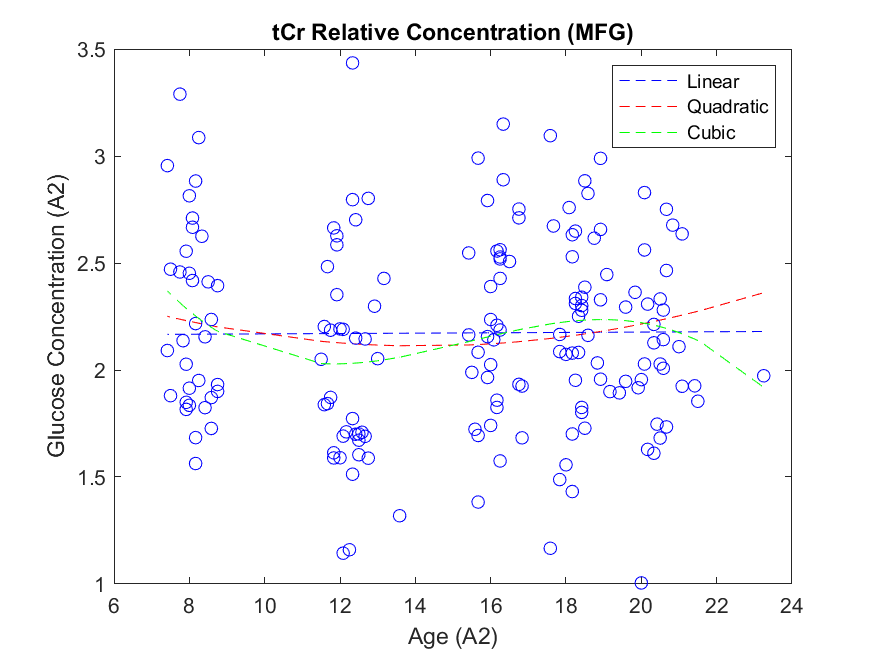

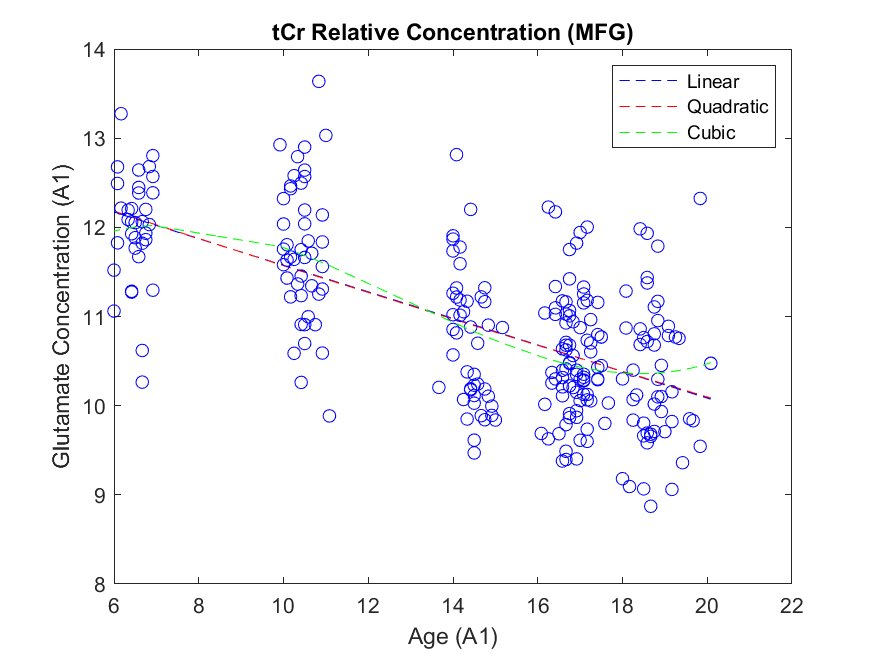

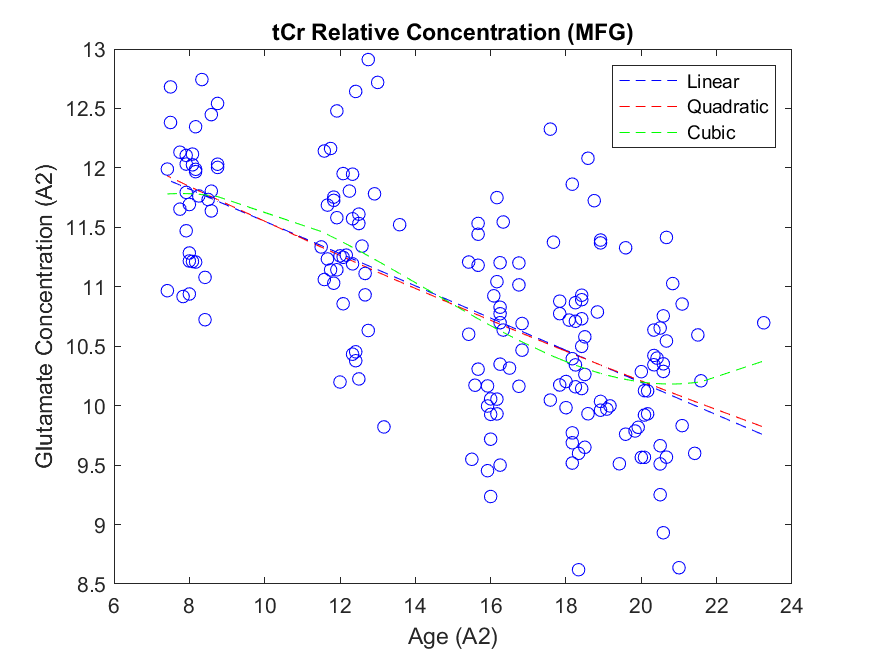

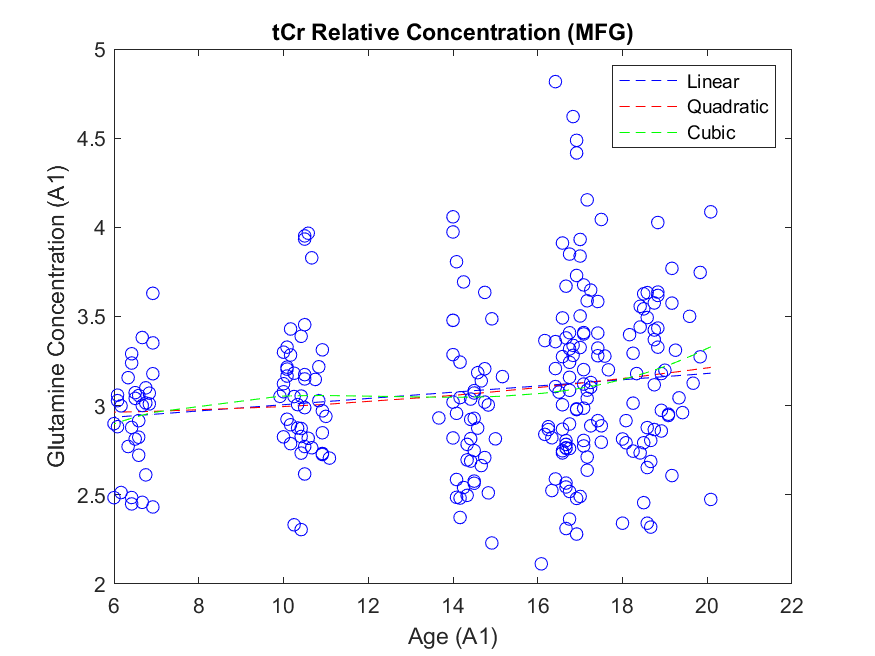

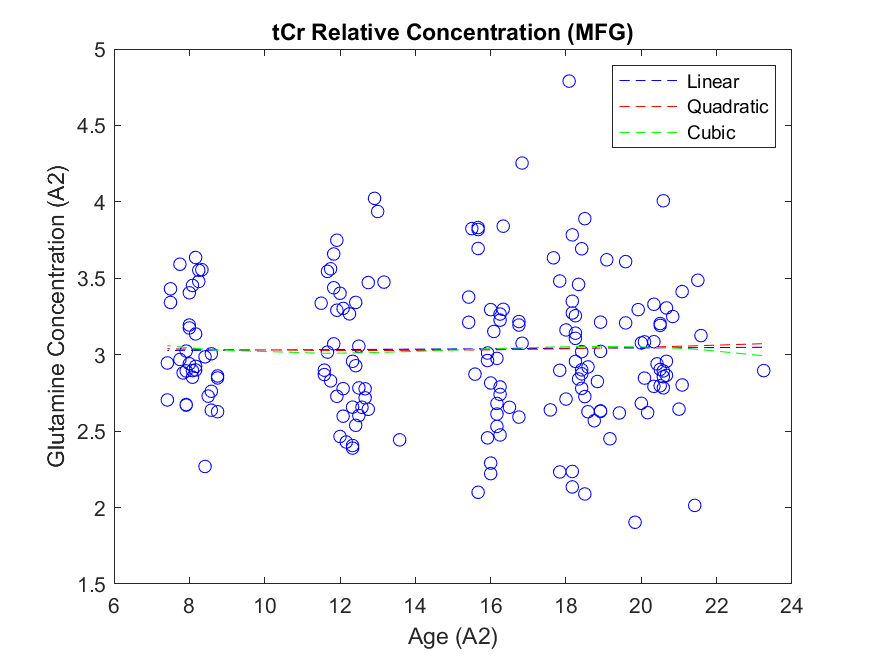

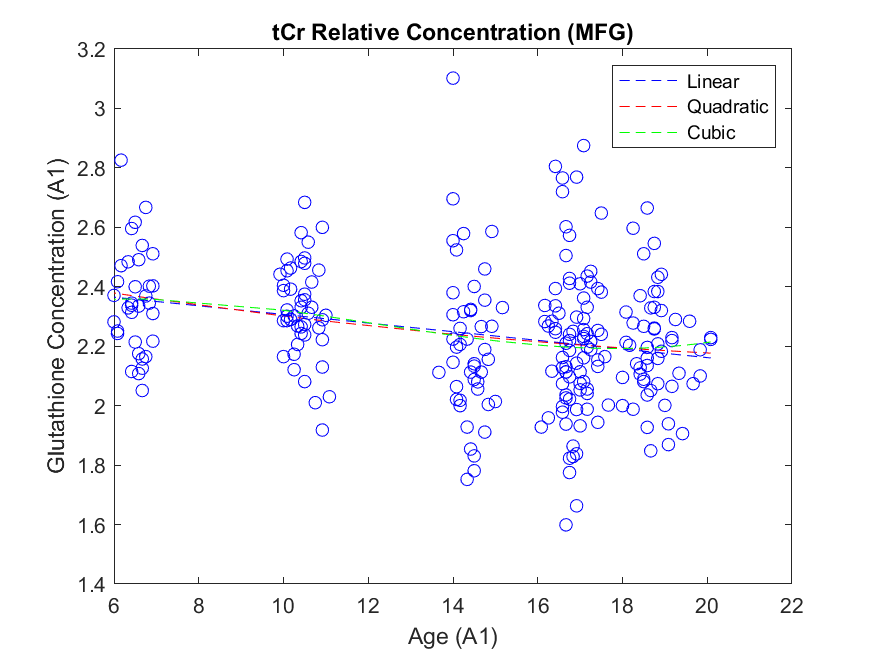

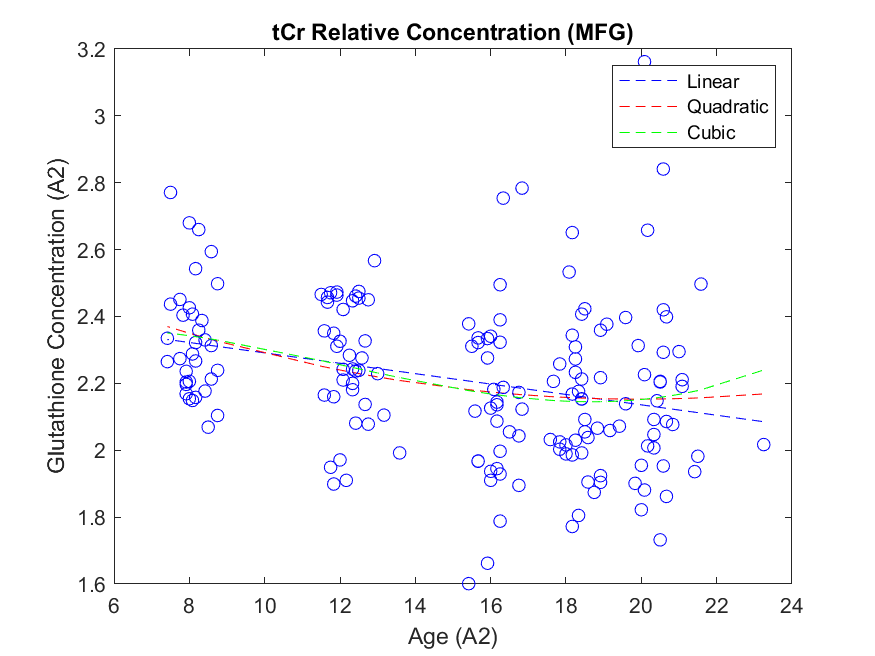

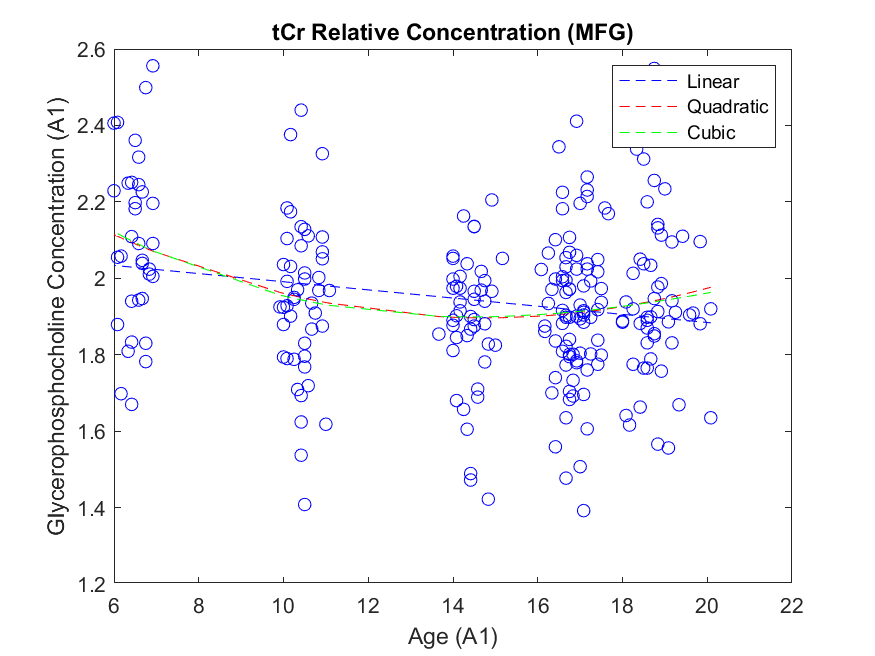

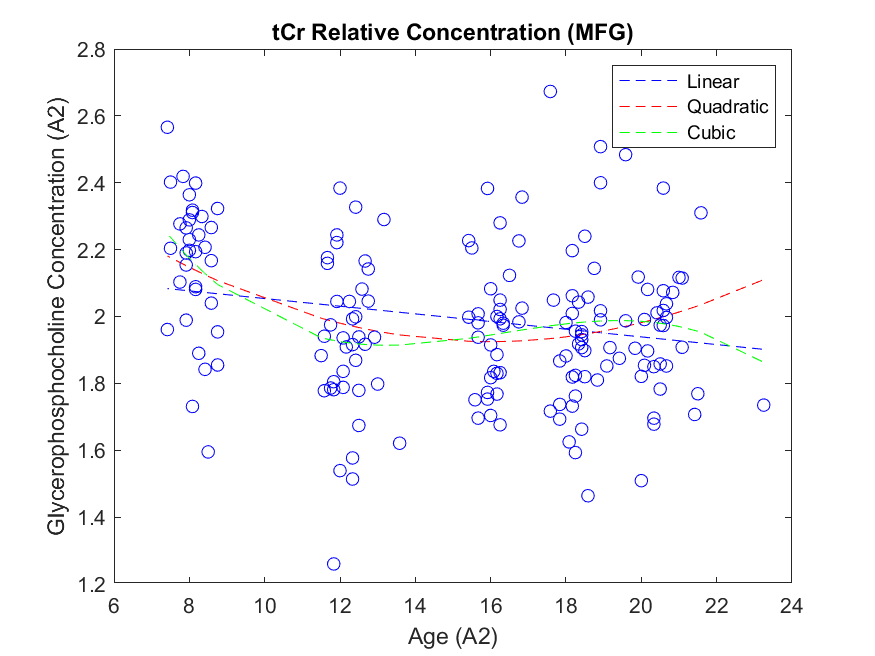

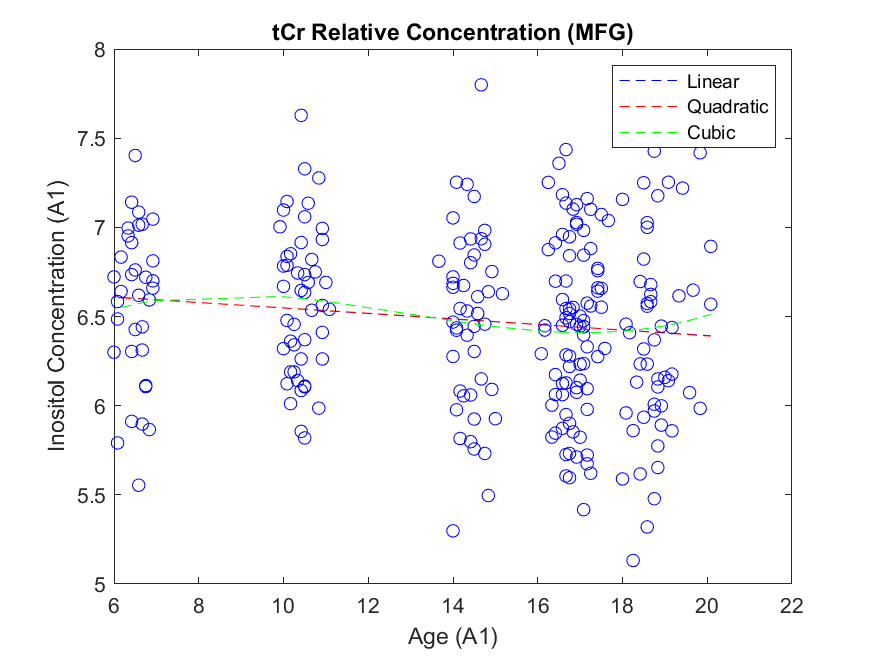

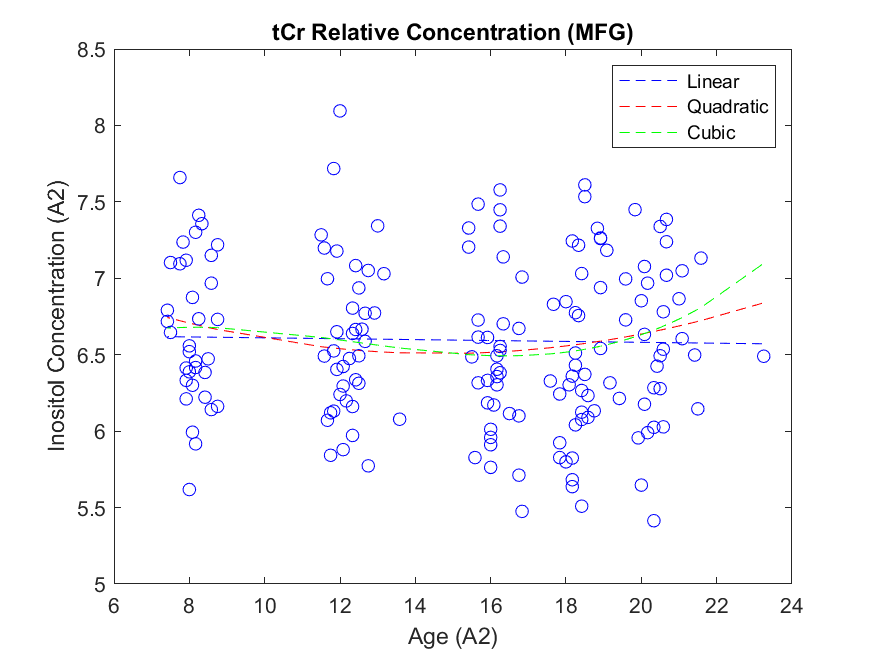
**

**Supporting Information 7.** Correlation matrix depicting the mean cross-correlations of the MFG and neurochemicals as well as neurochemical groups. For completeness, in this table, we did not use the exclusion criteria described in the main text in order to show the correlations of all 30 measures output from lcmodel. Rather, we excluded neurochemical-pair cases equalled to zero (per participants/per brain region), which likely reflects correlation between undetectable concentrations and could bias the mean correlations. Ala=Alanine, Asp=aspartate, Asc=ascorbate, Cr=creatine, GABA= gamma-aminobutyric acid, Glc=glucose, Gln=glutamine, GPC=glycerophosphocholine, GSH=glutathione, Ins=inositol, sIns=scyllo-Inositol, Lac=lactate, PCr= phosphocreatine, PCho=phosphocholine, PE=phosphoethanolamine, NAA=N-acetylaspartate, NAAG=N-acetylaspartylglutamate, Tau=taurine, the macromolecules that resonate at 0.9 ppm (MM09), at 2.0ppm (MM20), at 1.2ppm (MM12), at 1.4ppm (MM14), and at 1.7ppm (MM17), Scyllo=Scyllo, PChoG=phopshocholine+glycerophophocholine, CrPCr=creatine+phosphocreatine, NAANA=N-acetylaspartate+N-acetylaspartylglutamate, GluGl=glutamate+glutamine, GlcTau=glucose+taurine.

|  | Ala | Asp | Asc | Cr | GABA | Glc | Gln | Glu | GPC | GSH | Ins | sIns | Lac | PCr | PCho | PE | NAA | NAAG | Tau | MM09 | MM20 | MM12 | MM14 | MM17 | Scyllo | PChoG | CrPCr | NAANA | GluGl |
| --- | --- | --- | --- | --- | --- | --- | --- | --- | --- | --- | --- | --- | --- | --- | --- | --- | --- | --- | --- | --- | --- | --- | --- | --- | --- | --- | --- | --- | --- |
| Asp | -0.01 |  |  |  |  |  |  |  |  |  |  |  |  |  |  |  |  |  |  |  |  |  |  |  |  |  |  |  |  |
| Asc | 0.01 | -0.05 |  |  |  |  |  |  |  |  |  |  |  |  |  |  |  |  |  |  |  |  |  |  |  |  |  |  |  |
| Cr | 0.01 | -0.04 | 0.02 |  |  |  |  |  |  |  |  |  |  |  |  |  |  |  |  |  |  |  |  |  |  |  |  |  |  |
| GABA | 0.02 | 0.18 | -0.05 | -0.02 |  |  |  |  |  |  |  |  |  |  |  |  |  |  |  |  |  |  |  |  |  |  |  |  |  |
| Glc | -0.01 | -0.12 | -0.02 | 0.10 | 0.10 |  |  |  |  |  |  |  |  |  |  |  |  |  |  |  |  |  |  |  |  |  |  |  |  |
| Gln | 0.01 | 0.24 | -0.17 | 0.02 | 0.28 | -0.08 |  |  |  |  |  |  |  |  |  |  |  |  |  |  |  |  |  |  |  |  |  |  |  |
| Glu | -0.02 | 0.06 | -0.18 | 0.05 | -0.46 | -0.15 | -0.15 |  |  |  |  |  |  |  |  |  |  |  |  |  |  |  |  |  |  |  |  |  |  |
| GPC | 0.01 | 0.08 | 0.13 | 0.02 | 0.10 | 0.12 | -0.06 | -0.07 |  |  |  |  |  |  |  |  |  |  |  |  |  |  |  |  |  |  |  |  |  |
| GSH | -0.02 | 0.18 | -0.50 | 0.01 | 0.09 | -0.22 | 0.08 | 0.17 | -0.05 |  |  |  |  |  |  |  |  |  |  |  |  |  |  |  |  |  |  |  |  |
| Ins | 0.03 | 0.20 | 0.12 | 0.13 | 0.11 | 0.19 | 0.04 | 0.03 | 0.14 | 0.18 |  |  |  |  |  |  |  |  |  |  |  |  |  |  |  |  |  |  |  |
| sIns | 0.01 | 0.06 | 0.03 | 0.02 | 0.07 | 0.19 | 0.05 | -0.01 | 0.16 | 0.03 | 0.16 |  |  |  |  |  |  |  |  |  |  |  |  |  |  |  |  |  |  |
| Lac | 0.12 | 0.02 | 0.02 | 0.00 | 0.02 | 0.02 | 0.00 | -0.01 | 0.04 | 0.01 | 0.04 | 0.02 |  |  |  |  |  |  |  |  |  |  |  |  |  |  |  |  |  |
| PCr | 0.00 | 0.09 | 0.05 | -0.89 | -0.04 | -0.04 | -0.04 | 0.02 | 0.12 | -0.02 | 0.03 | 0.03 | 0.02 |  |  |  |  |  |  |  |  |  |  |  |  |  |  |  |  |
| PCho | 0.00 | 0.03 | -0.02 | 0.14 | -0.04 | -0.02 | -0.01 | 0.01 | -0.84 | -0.10 | -0.26 | 0.01 | -0.02 | -0.03 |  |  |  |  |  |  |  |  |  |  |  |  |  |  |  |
| PE | -0.01 | -0.06 | -0.21 | 0.02 | 0.02 | 0.17 | 0.09 | 0.05 | -0.61 | 0.15 | 0.03 | 0.09 | -0.01 | -0.13 | -0.03 |  |  |  |  |  |  |  |  |  |  |  |  |  |  |
| NAA | 0.00 | 0.04 | 0.01 | -0.01 | 0.22 | 0.00 | 0.06 | -0.04 | 0.13 | 0.06 | 0.11 | 0.04 | 0.02 | 0.10 | 0.16 | -0.05 |  |  |  |  |  |  |  |  |  |  |  |  |  |
| NAAG | 0.00 | -0.05 | -0.09 | 0.06 | -0.05 | -0.04 | 0.09 | 0.16 | -0.14 | 0.03 | -0.09 | -0.09 | -0.05 | -0.07 | -0.07 | 0.08 | -0.58 |  |  |  |  |  |  |  |  |  |  |  |  |
| Tau | 0.03 | 0.24 | 0.21 | -0.02 | 0.04 | -0.47 | 0.10 | 0.07 | 0.00 | 0.17 | 0.19 | -0.20 | 0.01 | 0.12 | -0.13 | -0.30 | 0.06 | -0.02 |  |  |  |  |  |  |  |  |  |  |  |
| MM09 | 0.01 | 0.02 | 0.01 | -0.01 | 0.03 | 0.00 | 0.01 | -0.04 | -0.01 | -0.02 | -0.01 | 0.01 | 0.01 | -0.02 | -0.02 | 0.01 | -0.02 | -0.11 | 0.00 |  |  |  |  |  |  |  |  |  |  |
| MM20 | 0.02 | -0.01 | 0.11 | -0.04 | 0.00 | 0.08 | -0.15 | -0.25 | 0.00 | -0.18 | -0.05 | 0.00 | 0.01 | -0.04 | -0.04 | -0.04 | -0.17 | -0.56 | -0.05 | 0.25 |  |  |  |  |  |  |  |  |  |
| MM12 | 0.04 | 0.02 | -0.01 | 0.00 | 0.04 | 0.00 | 0.03 | -0.02 | -0.01 | 0.00 | 0.00 | 0.01 | -0.06 | -0.02 | -0.01 | 0.01 | -0.01 | -0.03 | 0.01 | 0.41 | 0.15 |  |  |  |  |  |  |  |  |
| MM14 | -0.31 | 0.04 | -0.04 | 0.01 | 0.06 | -0.01 | 0.08 | 0.01 | -0.02 | 0.02 | 0.00 | 0.01 | -0.30 | -0.04 | -0.01 | 0.02 | -0.01 | 0.02 | 0.02 | 0.33 | 0.18 | 0.13 |  |  |  |  |  |  |  |
| MM17 | 0.03 | 0.08 | -0.05 | 0.00 | 0.13 | 0.00 | 0.11 | 0.02 | 0.02 | 0.05 | 0.04 | 0.02 | 0.04 | -0.01 | -0.02 | 0.00 | 0.08 | -0.02 | 0.04 | 0.29 | 0.23 | 0.28 | 0.19 |  |  |  |  |  |  |
| Scyllo | 0.00 | 0.00 | 0.00 | 0.00 | 0.00 | 0.00 | 0.00 | 0.00 | 0.01 | 0.00 | 0.00 | -0.04 | 0.00 | 0.00 | -0.01 | 0.00 | 0.01 | 0.00 | 0.00 | 0.00 | 0.00 | 0.00 | 0.00 | 0.00 |  |  |  |  |  |
| PChoG | 0.01 | 0.09 | 0.13 | 0.02 | 0.10 | 0.12 | -0.06 | -0.07 | 0.98 | -0.06 | 0.13 | 0.17 | 0.04 | 0.12 | 0.31 | -0.62 | 0.14 | -0.14 | -0.01 | -0.01 | 0.00 | -0.01 | -0.02 | 0.02 | 0.01 |  |  |  |  |
| CrPCr | 0.01 | 0.10 | 0.16 | 0.23 | -0.12 | 0.12 | -0.03 | 0.13 | 0.29 | -0.02 | 0.34 | 0.11 | 0.04 | 0.24 | 0.22 | -0.23 | 0.19 | -0.02 | 0.21 | -0.05 | -0.18 | -0.04 | -0.06 | -0.02 | 0.01 | 0.31 |  |  |  |
| NAANA | 0.00 | 0.02 | -0.01 | 0.01 | 0.22 | -0.01 | 0.09 | 0.01 | 0.10 | 0.07 | 0.09 | 0.01 | 0.01 | 0.08 | 0.16 | -0.03 | 0.88 | 0.24 | 0.06 | -0.05 | -0.34 | -0.02 | 0.00 | 0.08 | 0.01 | 0.10 | 0.20 |  |  |
| GluGl | -0.01 | 0.22 | -0.27 | 0.06 | -0.17 | -0.18 | 0.60 | 0.69 | -0.10 | 0.19 | 0.05 | 0.02 | 0.00 | -0.01 | 0.00 | 0.10 | 0.01 | 0.19 | 0.13 | -0.02 | -0.31 | 0.00 | 0.06 | 0.10 | 0.00 | -0.10 | 0.09 | 0.07 |  |
| GlcTau | 0.01 | 0.08 | 0.16 | 0.08 | 0.13 | 0.59 | 0.00 | -0.09 | 0.11 | -0.07 | 0.34 | 0.01 | 0.03 | 0.06 | -0.13 | -0.08 | 0.05 | -0.05 | 0.52 | 0.01 | 0.04 | 0.01 | 0.01 | 0.03 | 0.00 | 0.11 | 0.29 | 0.03 | -0.07 |

**Supporting Information 8.** Correlation matrix depicting the mean cross-correlations of the IPS and neurochemicals as well as neurochemical groups. For completeness, in this table, we did not use the exclusion criteria described in the main text in order to show the correlations of all 30 measures output from lcmodel. Rather, we excluded neurochemical-pair cases equalled to zero (per participants/per brain region), which likely reflects correlation between undetectable concentrations and could bias the mean correlations. Ala=Alanine, Asp=aspartate, Asc=ascorbate, Cr=creatine, GABA= gamma-aminobutyric acid, Glc=glucose, Gln=glutamine, GPC=glycerophosphocholine, GSH=glutathione, Ins=inositol, sIns=scyllo-Inositol, Lac=lactate, PCr= phosphocreatine, PCho=phosphocholine, PE=phosphoethanolamine, NAA=N-acetylaspartate, NAAG=N-acetylaspartylglutamate, Tau=taurine, the macromolecules that resonate at 0.9 ppm (MM09), at 2.0ppm (MM20), at 1.2ppm (MM12), at 1.4ppm (MM14), and at 1.7ppm (MM17), Scyllo=Scyllo, PChoG=phopshocholine+glycerophophocholine, CrPCr=creatine+phosphocreatine, NAANA=N-acetylaspartate+N-acetylaspartylglutamate, GluGl=glutamate+glutamine, GlcTau=glucose+taurine.

|  | Ala | Asp | Asc | Cr | GABA | Glc | Gln | Glu | GPC | GSH | Ins | sIns | Lac | PCr | PCho | PE | NAA | NAAG | Tau | MM09 | MM20 | MM12 | MM14 | MM17 | Scyllo | PChoG | CrPCr | NAANA | GluGl |
| --- | --- | --- | --- | --- | --- | --- | --- | --- | --- | --- | --- | --- | --- | --- | --- | --- | --- | --- | --- | --- | --- | --- | --- | --- | --- | --- | --- | --- | --- |
| Asp | -0.01 |  |  |  |  |  |  |  |  |  |  |  |  |  |  |  |  |  |  |  |  |  |  |  |  |  |  |  |  |
| Asc | 0.02 | -0.03 |  |  |  |  |  |  |  |  |  |  |  |  |  |  |  |  |  |  |  |  |  |  |  |  |  |  |  |
| Cr | 0.01 | -0.06 | 0.00 |  |  |  |  |  |  |  |  |  |  |  |  |  |  |  |  |  |  |  |  |  |  |  |  |  |  |
| GABA | 0.02 | 0.18 | -0.07 | -0.04 |  |  |  |  |  |  |  |  |  |  |  |  |  |  |  |  |  |  |  |  |  |  |  |  |  |
| Glc | -0.01 | -0.11 | 0.01 | 0.10 | 0.07 |  |  |  |  |  |  |  |  |  |  |  |  |  |  |  |  |  |  |  |  |  |  |  |  |
| Gln | 0.01 | 0.22 | -0.20 | 0.02 | 0.29 | -0.08 |  |  |  |  |  |  |  |  |  |  |  |  |  |  |  |  |  |  |  |  |  |  |  |
| Glu | -0.02 | 0.04 | -0.18 | 0.07 | -0.43 | -0.12 | -0.11 |  |  |  |  |  |  |  |  |  |  |  |  |  |  |  |  |  |  |  |  |  |  |
| GPC | 0.01 | 0.08 | 0.14 | 0.02 | 0.09 | 0.12 | -0.06 | -0.07 |  |  |  |  |  |  |  |  |  |  |  |  |  |  |  |  |  |  |  |  |  |
| GSH | -0.02 | 0.19 | -0.49 | 0.02 | 0.13 | -0.21 | 0.12 | 0.16 | -0.04 |  |  |  |  |  |  |  |  |  |  |  |  |  |  |  |  |  |  |  |  |
| Ins | 0.03 | 0.19 | 0.15 | 0.10 | 0.11 | 0.24 | 0.04 | 0.04 | 0.15 | 0.17 |  |  |  |  |  |  |  |  |  |  |  |  |  |  |  |  |  |  |  |
| sIns | 0.01 | 0.05 | 0.04 | 0.01 | 0.07 | 0.22 | 0.03 | -0.02 | 0.16 | 0.02 | 0.19 |  |  |  |  |  |  |  |  |  |  |  |  |  |  |  |  |  |  |
| Lac | 0.08 | 0.02 | 0.02 | 0.00 | 0.02 | 0.02 | 0.00 | -0.01 | 0.03 | 0.01 | 0.03 | 0.02 |  |  |  |  |  |  |  |  |  |  |  |  |  |  |  |  |  |
| PCr | 0.00 | 0.12 | 0.09 | -0.88 | -0.02 | -0.03 | -0.04 | -0.01 | 0.10 | -0.02 | 0.07 | 0.05 | 0.02 |  |  |  |  |  |  |  |  |  |  |  |  |  |  |  |  |
| PCho | -0.01 | 0.02 | -0.04 | 0.13 | -0.03 | -0.05 | -0.03 | 0.00 | -0.85 | -0.12 | -0.29 | 0.01 | -0.02 | -0.03 |  |  |  |  |  |  |  |  |  |  |  |  |  |  |  |
| PE | -0.01 | -0.07 | -0.20 | 0.00 | 0.03 | 0.20 | 0.09 | 0.05 | -0.56 | 0.14 | 0.04 | 0.10 | -0.01 | -0.09 | -0.04 |  |  |  |  |  |  |  |  |  |  |  |  |  |  |
| NAA | 0.00 | 0.05 | 0.04 | -0.04 | 0.20 | 0.00 | 0.04 | -0.07 | 0.11 | 0.05 | 0.11 | 0.06 | 0.02 | 0.13 | 0.14 | -0.04 |  |  |  |  |  |  |  |  |  |  |  |  |  |
| NAAG | 0.00 | -0.08 | -0.11 | 0.11 | -0.07 | -0.04 | 0.08 | 0.21 | -0.11 | 0.03 | -0.10 | -0.11 | -0.05 | -0.12 | -0.06 | 0.05 | -0.60 |  |  |  |  |  |  |  |  |  |  |  |  |
| Tau | 0.02 | 0.20 | 0.20 | -0.03 | 0.06 | -0.42 | 0.08 | 0.04 | 0.03 | 0.15 | 0.20 | -0.15 | 0.01 | 0.13 | -0.13 | -0.30 | 0.06 | -0.05 |  |  |  |  |  |  |  |  |  |  |  |
| MM09 | 0.02 | 0.00 | 0.02 | -0.01 | 0.02 | 0.01 | -0.01 | -0.05 | 0.00 | -0.03 | -0.01 | 0.01 | 0.01 | -0.01 | -0.01 | 0.01 | -0.01 | -0.09 | 0.01 |  |  |  |  |  |  |  |  |  |  |
| MM20 | 0.02 | -0.01 | 0.12 | -0.06 | 0.02 | 0.07 | -0.15 | -0.26 | 0.02 | -0.17 | -0.03 | 0.03 | 0.01 | -0.02 | -0.01 | -0.03 | -0.02 | -0.51 | -0.01 | 0.25 |  |  |  |  |  |  |  |  |  |
| MM12 | 0.03 | 0.00 | 0.00 | 0.00 | 0.02 | 0.00 | 0.01 | -0.03 | -0.01 | -0.02 | -0.01 | 0.01 | -0.03 | -0.02 | -0.01 | 0.01 | -0.01 | -0.03 | 0.01 | 0.44 | 0.14 |  |  |  |  |  |  |  |  |
| MM14 | -0.22 | 0.02 | -0.04 | 0.02 | 0.04 | -0.01 | 0.07 | 0.01 | -0.02 | 0.01 | -0.02 | 0.01 | -0.25 | -0.05 | -0.01 | 0.03 | -0.04 | 0.03 | 0.01 | 0.35 | 0.17 | 0.13 |  |  |  |  |  |  |  |
| MM17 | 0.05 | 0.05 | -0.04 | 0.01 | 0.13 | 0.00 | 0.11 | 0.02 | 0.01 | 0.03 | 0.03 | 0.03 | 0.02 | -0.03 | -0.02 | 0.02 | 0.04 | 0.01 | 0.04 | 0.30 | 0.26 | 0.29 | 0.28 |  |  |  |  |  |  |
| Scyllo | 0.00 | 0.00 | 0.00 | 0.00 | 0.00 | 0.00 | 0.00 | 0.00 | 0.01 | 0.00 | 0.00 | -0.05 | 0.00 | 0.00 | -0.01 | 0.00 | 0.01 | 0.00 | 0.00 | 0.00 | 0.00 | 0.00 | 0.00 | 0.00 |  |  |  |  |  |
| PChoG | 0.01 | 0.08 | 0.14 | 0.03 | 0.09 | 0.12 | -0.07 | -0.07 | 0.92 | -0.05 | 0.12 | 0.17 | 0.03 | 0.10 | 0.34 | -0.59 | 0.14 | -0.13 | 0.02 | 0.00 | 0.02 | -0.01 | -0.02 | 0.00 | 0.01 |  |  |  |  |
| CrPCr | 0.01 | 0.11 | 0.18 | 0.25 | -0.12 | 0.13 | -0.04 | 0.13 | 0.24 | -0.02 | 0.34 | 0.12 | 0.04 | 0.24 | 0.21 | -0.18 | 0.18 | -0.03 | 0.20 | -0.05 | -0.16 | -0.03 | -0.06 | -0.05 | 0.01 | 0.27 |  |  |  |
| NAANA | 0.00 | 0.02 | -0.03 | 0.02 | 0.19 | -0.02 | 0.10 | 0.05 | 0.06 | 0.07 | 0.07 | -0.01 | 0.00 | 0.07 | 0.13 | -0.02 | 0.77 | 0.19 | 0.04 | -0.07 | -0.34 | -0.03 | -0.03 | 0.05 | 0.01 | 0.08 | 0.18 |  |  |
| GluGl | -0.01 | 0.19 | -0.28 | 0.08 | -0.14 | -0.15 | 0.61 | 0.72 | -0.09 | 0.21 | 0.06 | 0.00 | 0.00 | -0.04 | -0.02 | 0.10 | -0.03 | 0.22 | 0.08 | -0.05 | -0.31 | -0.02 | 0.06 | 0.09 | 0.00 | -0.10 | 0.08 | 0.10 |  |
| GlcTau | 0.01 | 0.07 | 0.19 | 0.07 | 0.13 | 0.57 | -0.01 | -0.08 | 0.14 | -0.07 | 0.40 | 0.07 | 0.03 | 0.08 | -0.15 | -0.07 | 0.06 | -0.08 | 0.52 | 0.01 | 0.05 | 0.01 | 0.00 | 0.03 | 0.00 | 0.12 | 0.30 | 0.01 | -0.07 |

**Supporting Information 9.** Table depicting the results of tests that assess multiple regression assumptions. We assessed the presence of multicollinearity using the variance inflation factor (VIF), the presence of heteroscedasticity using the Breusch-Pagan test (BP), and the normality of residuals using the Shapiro-Wilk test (SW). We additionally show the standardized coefficient of the interaction term age* IPS glutamate, the conventional p-value (P) and the p-value derived from bootstrapping (PBO) using 5000 samples (95% confidence intervals). Please note that here we do not assess whether these models are significant as this would constitute reverse inference since these data were already deemed significant in the seed-to-voxel connectivity analyses described in the **Methods** section. The reason we performed these test is that the CONN toolbox do not necessarily assess these assumption when running the statistical models.

|  |  | **β** | **P** | **PBO** | **VIF** | **BP** | **SW** |
| --- | --- | --- | --- | --- | --- | --- | --- |
|  | cingulate gyrus | -0.35 | <.001 | <.001 | 1.72 | 0.53 | 0.03 |
|  | right inferior/middle temporal gyrus | -0.34 | <.001 | <.001 | 1.72 | 0.68 | 0.69 |
|  | left middle temporal gyrus | -0.34 | <.001 | <.001 | 1.72 | 0.87 | 0.26 |
|  | right angular gyrus | -0.32 | <.001 | <.001 | 1.72 | 0.58 | 0.95 |
|  | right middle temporal gyrus | -0.33 | <.001 | <.001 | 1.72 | 0.17 | 0.12 |
|  | superior frontal gyrus | -0.31 | <.001 | <.001 | 1.72 | 0.1 | 0.73 |
|  | left occipital/angular gyrus | -0.29 | <.001 | <.001 | 1.72 | 0.5 | 0.71 |

**Supporting Information 10.** Additional information about conversion of units for neurochemicals, specifically from the water reference quantitation approach. The mass of 1000 mL of water is 1000 g, and the molecular weight of water is around 18 gmol−1, therefore the calculation gives us an answer of 55556 mM.

**Metabolite concentration** = (absolute concentration) * (metabolite area / water area)

**Relaxation and partial Volume corrected water concentration** = (1 mol / 18.015 g * White matter Tissue content * 0.65 g/ml * exp(-TE ms/T2 of Water ms) + 1 mol/18.015 g * Gray matter tissue content *0.78g/ml * exp(-TE ms/T2 of Water ms) +1 mol/18.015 g * CSF content *exp(-TE ms/T2 of Water ms) )/ (1-CSF content)  * metabolite concentration

**ALL REFERENCES**

Button, Katherine S, Ioannidis, John PA, Mokrysz, Claire, Nosek, Brian A, Flint, Jonathan, Robinson, Emma SJ, & Munafò, Marcus R. (2013). Power failure: why small sample size undermines the reliability of neuroscience. *Nature reviews neuroscience, 14*(5), 365.

Charles, H Cecil, Lazeyras, Francois, Krishnan, KR, Boyko, Orest B, Patterson, Linda J, Doraiswamy, P Murali, & Mcdonald, William M. (1994). Proton spectroscopy of human brain: effects of age and sex. *Progress in neuro-psychopharmacology & biological psychiatry, 18*(6), 995-1004.

Cohen-Gilbert, Julia E, Jensen, J Eric, & Silveri, Marisa M. (2014). Contributions of magnetic resonance spectroscopy to understanding development: Potential applications in the study of adolescent alcohol use and abuse. *Development and psychopathology, 26*(2), 405-423.

Delvenne, Véronique, Goldman, Serge, De Maertelaer, Viviane, Simon, Yves, Luxen, André, & Lotstra, Françoise. (1996). Brain hypometabolism of glucose in anorexia nervosa: normalization after weight gain. *Biological Psychiatry, 40*(8), 761-768.

Nagae‐Poetscher, Lidia M, Bonekamp, David, Barker, Peter B, Brant, Larry J, Kaufmann, Walter E, & Horská, Alena. (2004). Asymmetry and gender effect in functionally lateralized cortical regions: a proton MRS imaging study. *Journal of Magnetic Resonance Imaging: An Official Journal of the International Society for Magnetic Resonance in Medicine, 19*(1), 27-33.

O'Gorman, Ruth L, Michels, Lars, Edden, Richard A, Murdoch, James B, & Martin, Ernst. (2011). In vivo detection of GABA and glutamate with MEGA‐PRESS: reproducibility and gender effects. *Journal of Magnetic Resonance Imaging, 33*(5), 1262-1267.

Pardridge, William M, Boado, Ruben J, & Farrell, Christine R. (1990). Brain-type glucose transporter (GLUT-1) is selectively localized to the blood-brain barrier. Studies with quantitative western blotting and in situ hybridization. *Journal of Biological Chemistry, 265*(29), 18035-18040.

Rist, Manuela J, Roth, Alexander, Frommherz, Lara, Weinert, Christoph H, Krüger, Ralf, Merz, Benedikt, . . . Bub, Achim. (2017). Metabolite patterns predicting sex and age in participants of the Karlsruhe Metabolomics and Nutrition (KarMeN) study. *PloS one, 12*(8), e0183228.

Tayoshi, Shin'Ya, Sumitani, Satsuki, Taniguchi, Kyoko, Shibuya-Tayoshi, Sumiko, Numata, Shusuke, Iga, Jun-ichi, . . . Ohmori, Tetsuro. (2009). Metabolite changes and gender differences in schizophrenia using 3-Tesla proton magnetic resonance spectroscopy (1H-MRS). *Schizophrenia research, 108*(1-3), 69-77.
